# Supplementary material for: Magnetospheric Control of Ionospheric TEC Perturbations via Whistler‐Mode and ULF Waves
Source: AGU Adv. 2024 Nov 25;5(6):e2024AV001302. doi: 10.1029/2024AV001302 (PMC11600791; doi:10.1029/2024AV001302)
Supplement: Supplementary file 1 — Supporting Information S1 [file AGA2-5-0-s001.pdf]

AGU Advances

Supporting Information for "Magnetospheric control of ionospheric TEC perturbations via whistler-mode and ULF waves"

Yangyang Shen[1], Olga P. Verkhoglyadova[2], Anton Artemyev[1], Michael D. Hartinger[3,1], Vassilis Angelopoulos[1], Xueling Shi[4], Ying Zou[5]

[1]Department of Earth, Planetary, and Space Sciences, University of California, Los Angeles, CA, USA

[2]Jet Propulsion Laboratory, California Institute of Technology, Pasadena, CA, USA

[3]Space Science Institute, Center for Space Plasma Physics, Boulder, CO, USA

[4]Department of Electrical and Computer Engineering, Virginia Tech, Blacksburg, VA, USA

[5]Johns Hopkins University Applied Physics Laboratory, Laurel, MD, USA

Contents of this file

1. Figure S1
2. Figure S2
3. Figure S3
4. Figure S4

Introduction

In this Supporting Information, we include Figure S1, displaying the ground-based ionosonde measurements from the Eielson station (64.66ºN, 212.03Eº). The ionograms were available every 15 min and we show here the example from 15:45 UT on 3 July 2013, relevant to the dTEC measured in Figure 2i in the main text. There was an ionosphere F2 layer located at ∼300 km altitude during this event, indicated by the hmF2 parameter

in Figure S1. This F2 layer were there for a long time, varying from 309.7 km at 15:30 UT to 307.9 km at 15:45 UT, and to 281.1 km at 16:00 UT (not shown here). Based on these ground-based ionogram observations, we set the GPS satellites ionosphere pierce point to be at 300 km altitude.

We include Figure S2 to illustrate the configuration of THEMIS E footpaths and GPS satellite raypaths with the ionospheric pierce point adjusted to 150 km altitude, compared with the pierce point of 300 km depicted in Figure 2a of the main text. This adjustment retains the good longitudinal alignment between THEMIS E footpaths and GPS43 pierce points, bringing the GPS satellite pierce points closer to FAIR than shown in Figure 2a. However, unlike in Figure 2a where THEMIS-E footprints appear south of FAIR, they are now positioned to the north. The observed latitudinal shifts of less than 0.5° fall within the mapping uncertainties of the Tsyganenko T96 model.

We include one additional Figure (S3) displaying whistler-mode wave spectra and the electric to magnetic field ratio (E/cB), where c is the speed of light. The E/cB corresponds to $1/N$, where N is the wave refractive index. Small E/cB <1 implies that the waves have small wave normal angles, therefore mostly propagating parallel to the background magnetic field.

We also include one Figure (S4) illustrating the modulation of whistler-mode wave amplitudes by ULF waves in the Pc3–4 band (∼6.7–100 mHz). The observed ULF poloidal mode waves seem to modulate the whistler-mode wave growth in our event. The modulation may be associated with the enhancements of thermal plasma density variations (Panels d–f). The density variations may be associated with nonlinear effects of field-line resonances due to ponderomotive effects (e.g., Streltsov & Lotko, 2008).

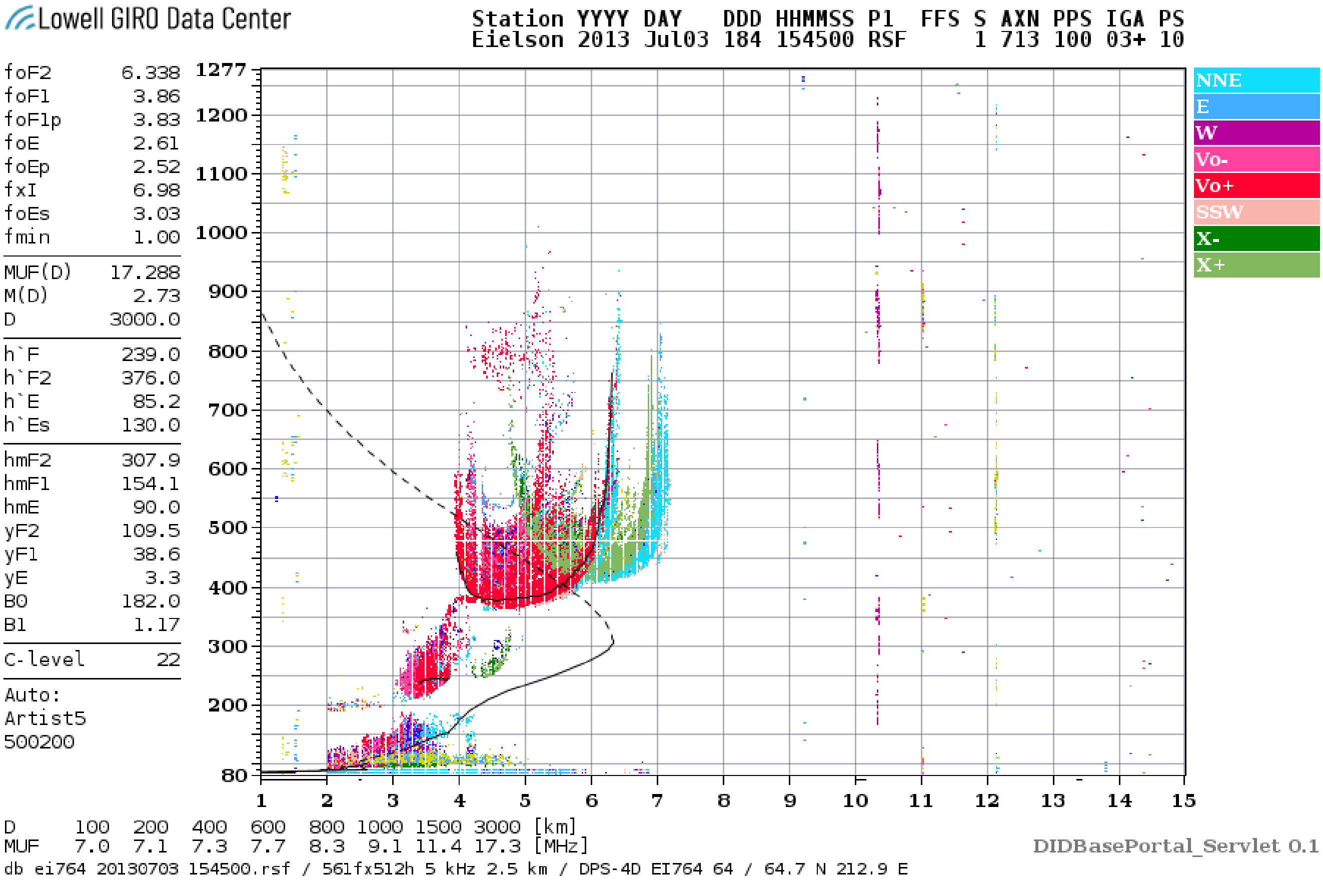

Figure S1. Ionogram measured near 15:45 UT showing the fitted F2-region peak density height hmF2 of ∼300 km, the F1-region peak density height of 154 km, and the E-region peak density height of 90 km. The inferred background density profile is indicated by the black and dashed lines.

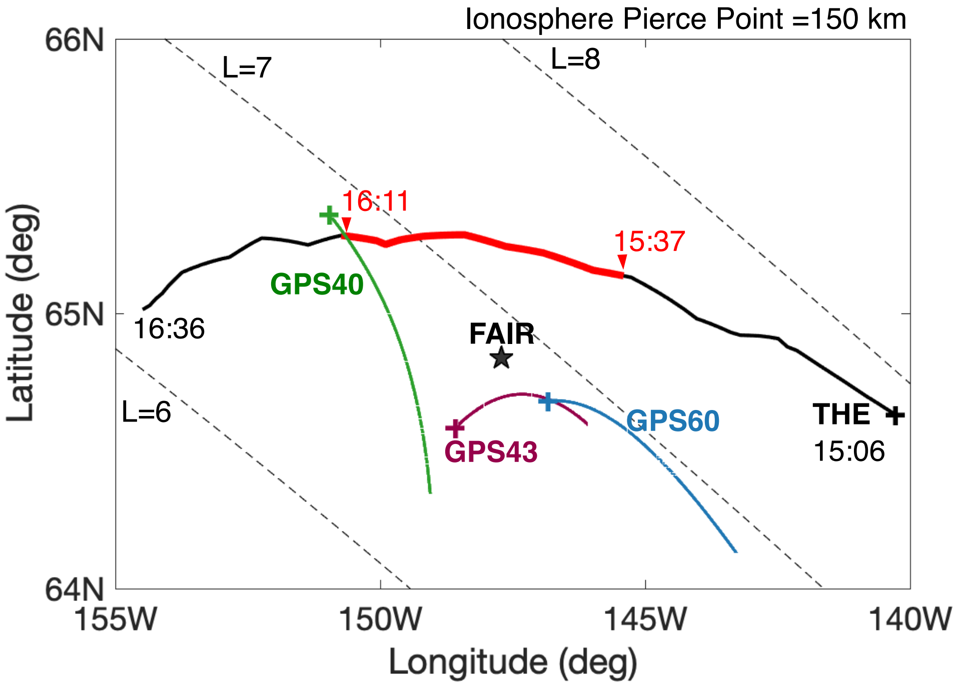

Figure S2. Configuration of THEMIS-E (THE, black curve), GPS40, GPS43, and GPS60 satellites (green, purple, and blue curves), and the FAIR receiver (black star) in geographic coordinates, with THEMIS and GPS mapped onto 150 km altitude using T96 field tracing (THEMIS) or line of sight (GPS). The plus symbol indicates the start of the footpath.

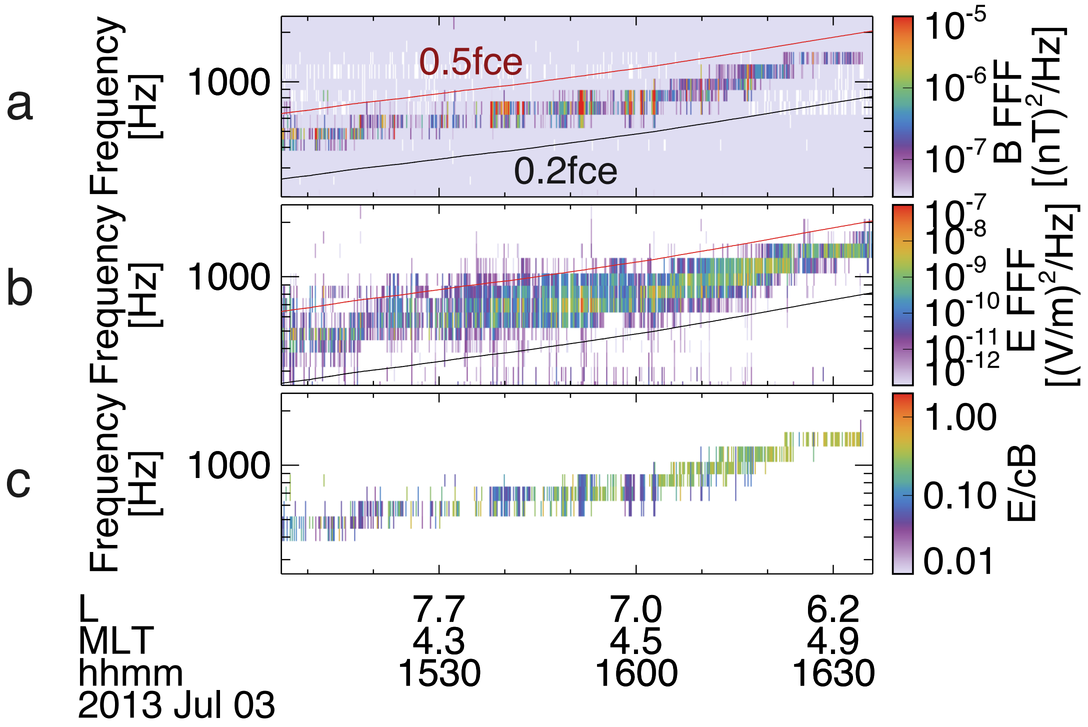

Figure S3. (a) THEMIS-E wave magnetic field spectrogram. (b) THEMIS-E wave electric field spectrogram. (c) Whistler-mode wave E/cB ratio spectrogram. The waves with B_w^2 less than 5×10^{-8} (nT)^2/Hz have been excluded in this calculation.

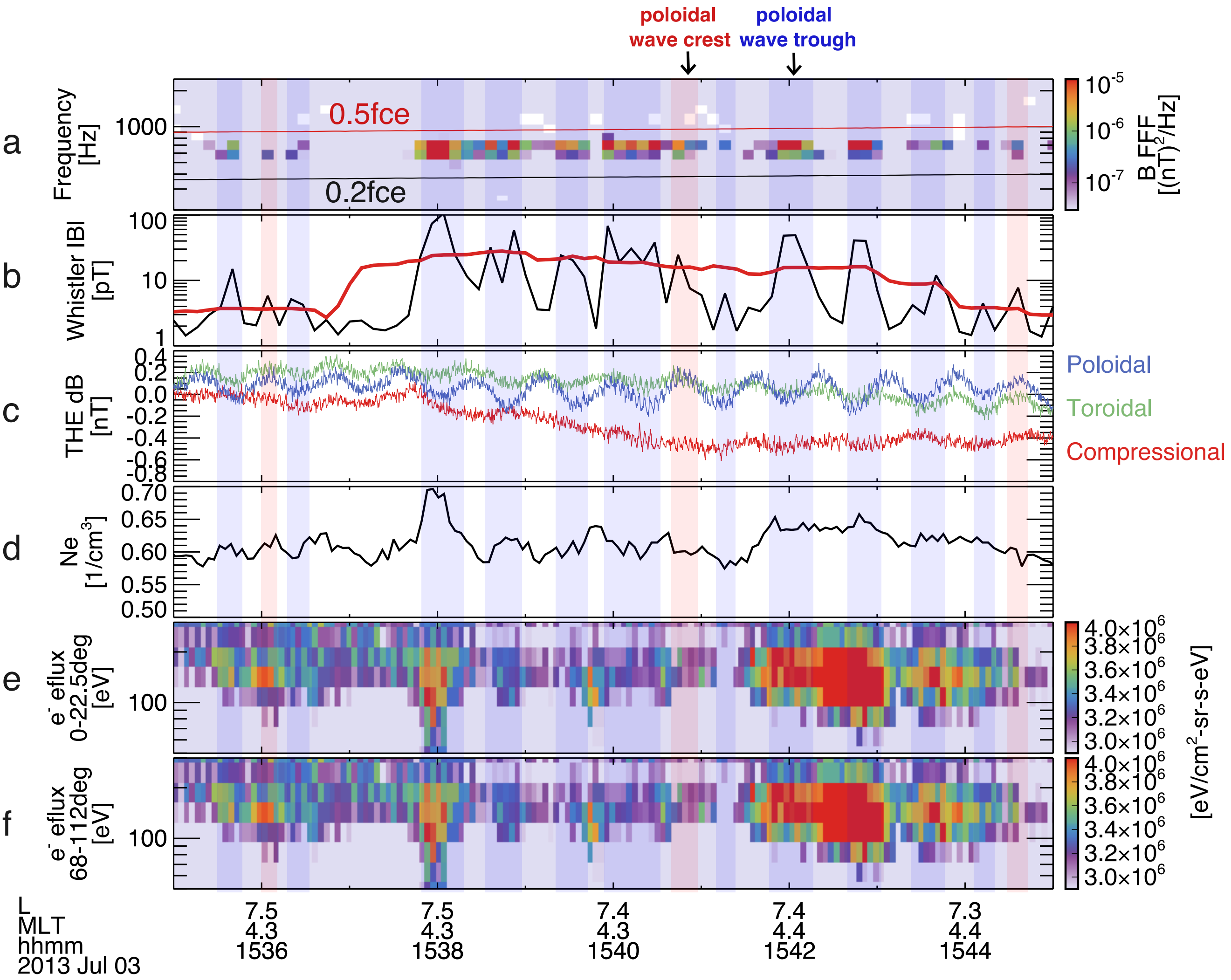

Figure S4. Zoomed-in view of small-scale whistler-mode wave amplitudes modulated by Pc3–4 poloidal ULF waves during 15:35–15:45 UT on July 3, 2013. Most B_w peaks are associated with poloidal mode wave troughs. (a) THEMIS-E wave magnetic field spectrogram. (b) Whistler-mode wave amplitude variations. The red curve shows amplitudes smoothed by applying 2-min sliding averaging. (c) Magnetic field perturbations in the poloidal, toroidal, and compressional components (see main text). (d) Electron density variations. (e) Parallel electron energy spectrogram from 50 eV up to 500 eV. (f) Perpendicular electron energy spectrogram from 50 ev up to 500 eV.

References

Streltsov, A. V., & Lotko, W. (2008, May). Coupling between density structures, electromagnetic waves and ionospheric feedback in the auroral zone. *Journal of Geophysical Research (Space Physics)*, *113*(A5), A05212. doi: 10.1029/2007JA012594
