# Supplementary material for: Magnetospheric Control of Ionospheric TEC Perturbations via Whistler‐Mode and ULF Waves
Source: AGU Adv. 2024 Nov 25;5(6):e2024AV001302. doi: 10.1029/2024AV001302 (PMC11600791; doi:10.1029/2024AV001302)
Supplement: Supplementary file 2 — Original Version of Manuscript [file AGA2-5-0-s008.pdf]

Magnetospheric control of ionospheric TEC perturbations via whistler-mode and ULF waves

Yangyang Shen[1], Olga P. Verkhoglyadova[2], Anton Artemyev[1], Michael D. Hartinger[3,1], Vassilis Angelopoulos[1], Xueling Shi[4], Ying Zou[5]

[1]Department of Earth, Planetary, and Space Sciences, University of California, Los Angeles, CA, USA
[2]Jet Propulsion Laboratory, California Institute of Technology, Pasadena, CA, USA
[3]Space Science Institute, Center for Space Plasma Physics, Boulder, CO, USA
[4]Department of Electrical and Computer Engineering, Virginia Tech, Blacksburg, VA, USA
[5]Johns Hopkins University Applied Physics Laboratory, Laurel, MD, USA

Key Points:

- Space-ground conjugate observations point to magnetospheric whistler-mode waves as the driver of ionospheric TEC perturbations (dTEC)
- The amplitude spectra of dTEC and whistlers are consistent and the cross-correlation between modeled and observed dTEC reaches 0.8
- Whistler-mode wave amplitudes and dTEC are modulated by ULF waves, which exhibit concurrent compressional and poloidal mode variations

Corresponding author: Yangyang Shen, `yshen@epss.ucla.edu`

Abstract

Total electron content (TEC) in the ionosphere is vital for space weather applications, including monitoring Global Navigation Satellite Systems (GNSS) scintillations and detecting natural hazards, making comprehensive understanding, modeling, and forecasting of TEC perturbations critical for modern society. While modulations of TEC perturbations (dTEC) in the auroral region have been observed to be associated with ultra-low-frequency (ULF) waves, their driving mechanisms remain unclear. Using fortuitously timed and positioned conjugate observations from the THEMIS spacecraft and a GPS receiver at Fairbanks, Alaska, we provide direct evidence that dTEC modulations are driven by magnetospheric precipitation due to electron scattering into the loss cone by ULF-modulated whistler-mode waves. Peak-to-peak dTEC amplitudes reached ∼0.5 TECU with modulations spanning scales of ∼5–80 km. The cross-correlation between modeled and observed dTEC reached ∼0.8 during the conjugacy period but decreased outside of it. The amplitude spectra of whistler-mode waves and dTEC also matched closely from 1 mHz to tens of mHz during the conjugacy period but diverged outside of it. Our findings offer crucial insights that could improve physics-based TEC modeling and ultimately enhance TEC forecast capabilities.

Plain Language Summary

Radio signals experience group delay and phase advance as they transit the ionosphere filled with free electrons. The total number of these electrons along the raypath from the satellite to a receiver defines the total electron content (TEC) of the ionosphere. Small-scale number density irregularities or TEC perturbations may be associated with rapid phase and amplitude fluctuations that cause GNSS signal flickering or scintillations. Scintillations degrade positioning accuracy, disrupt navigation and communication, and lead to signal reception failures. Thus, precise understanding, modeling, and forecasting of TEC perturbations (dTEC) is vital for space weather monitoring and GNSS scintillation prediction. In this study, we analyze fortuitously conjugate observations of ionospheric dTEC from a ground-based GPS receiver and magnetospheric very-low-frequency (VLF) whistler-mode waves from the THEMIS spacecraft, which were optimally aligned in time and space. We find consistent amplitude spectra of dTEC and whistler-mode waves during conjunction, as well as a good cross-correlation (∼0.8) between observed and modeled dTEC, pointing to whistler-mode waves as the driver of the observed ionospheric

dTEC. The dTEC and whistler-mode wave amplitudes were modulated by ultra-low-frequency (ULF) waves. Our findings offer crucial insights that could ultimately enhance forecast capabilities of auroral TEC models.

1 Introduction

One of the first observed space weather phenomena was phase and amplitude fluctuations of radio signals traversing the inhomogeneous ionosphere (Hey et al., 1946). Rapid or small-scale fluctuations can cause signal scintillations of the Global Navigation Satellite Systems (GNSS), degrading positioning accuracy, disrupting navigation and communication, and leading to signal reception failures or loss of lock (Yeh & Liu, 1982; Kintner et al., 2007). The ionospheric signal errors, if caused by refractive effects, can be mitigated by using dual-frequency reception to navigate the dispersive ionosphere and compensating for differential phase delays in the calculated total electron content (TEC) (Mannucci et al., 1998; Ciraolo et al., 2007; McCaffrey & Jayachandran, 2017). The rate of TEC changes serves as an indicator for predicting GNSS scintillations (Pi et al., 1997; Makarevich et al., 2021). Furthermore, relative TEC perturbations have been used to monitor natural hazards, such as tsunamis, earthquakes, explosions, and volcanic eruptions (Komjathy et al., 2016; Astafyeva, 2019). Consequently, precise understanding, modeling, and forecasting of ionospheric TEC perturbations is critical for modern society (Kintner et al., 2007; Jakowski et al., 2007, 2011).

Strong small-scale ($<\sim$100 km) TEC perturbations have been observed across both low and high latitudes due to a multitude of mechanisms and drivers involved (Pi et al., 1997; Basu et al., 2002; Kintner et al., 2007; Moen et al., 2013; Pilipenko et al., 2014; Jin et al., 2015; Prikryl et al., 2015; Watson, Jayachandran, Singer, et al., 2016; Fæhn Follestad et al., 2020). Near equatorial latitudes, the most intense ionospheric TEC perturbations and GPS scintillations result from irregularities of plasma bubbles or equatorial spread F, driven by the Rayleigh-Taylor instability due to lower atmosphere-ionosphere coupling processes (C.-S. Huang & Kelley, 1996; Kelley, 2009; Xiong et al., 2010; Aa et al., 2020; Jin et al., 2020). At higher latitudes, TEC perturbations are caused by plasma irregularities in the cusp and polar cap regions, ranging from a few meters to hundreds of kilometers and associated with solar wind-magnetosphere-ionosphere coupling processes (e.g., Basu et al., 1990; Moen et al., 2013; Spicher et al., 2017). These polar cap irregularities, or density patches, emerge from complex dynamics involving EUV radi-

ation, convection, cusp precipitation, field-aligned currents, and various plasma instabilities (Kintner et al., 2007; Moen et al., 2013; Fæhn Follestad et al., 2020).

TEC perturbations have also been frequently observed in the auroral region, where structured particle precipitation prevails (Basu et al., 1983; Coker et al., 1995; Newell et al., 2009; Nishimura et al., 2010; Chaston et al., 2003; Watson et al., 2011; Watson, Jayachandran, Singer, et al., 2016; Kasahara et al., 2018; Liang et al., 2019; Shen et al., 2020; Nishimura et al., 2023). This auroral precipitation, often accompanied by plasma flow shears, can induce plasma instabilities and density gradients on scales down to the first Fresnel (diffractive) zone of hundreds of meters (Fejer & Kelley, 1980; Keskinen & Ossakow, 1983; Tsunoda, 1988; Semeter et al., 2017). Statistical studies have showed that strong TEC perturbations and GPS phase scintillations were closely collocated in the auroral region (e.g., Spogli et al., 2009; Jin et al., 2015; Prikryl et al., 2015; Makarevich et al., 2021).

Previous studies have identified modulations of TEC perturbations near the auroral region associated with ultra-low-frequency (ULF) waves (Davies & Hartmann, 1976; Okuzawa & Davies, 1981; Skone, 2009; Pilipenko et al., 2014; Watson et al., 2015; Watson, Jayachandran, Singer, et al., 2016; Zhai et al., 2021). Skone (2009) noted that average power of magnetic and TEC perturbations in the Pc3 band ($\sim$0.02–0.1 Hz) displayed similar temporal variations. Pilipenko et al. (2014) observed a high coherence ($\sim$0.9) between TEC perturbations and global Pc5 pulsations in a few millihertz during a geomagnetic storm. High coherence and significant common power between TEC perturbations and ULF radial magnetic field variations in the Pc4 band were also reported by Watson, Jayachandran, Singer, et al. (2016). Despite proposals for various direct TEC modulation mechanisms by ULF waves (Pilipenko et al., 2014), no mechanism has yet been conclusively established.

Combining spacecraft and ground-based radar observations, Wang et al. (2020) reported a storm-time event where duskside ionospheric density was modulated by ULF waves in the Pc5 range. Density inversion suggests that the Pc5 pulsations modulated precipitating electrons over an energy range of $\sim$1–500 keV and an altitude range of $\sim$80–200 km. The authors postulated that these precipitation and density perturbations are likely due to electron scattered into the loss cone by ULF-modulated very-low-frequency (VLF) whistler-mode waves.

Numerous observations and models demonstrate that ULF waves coexist with and modulate whistler-mode chorus waves (Coroniti & Kennel, 1970; W. Li, Thorne, et al., 2011; W. Li, Bortnik, Thorne, Nishimura, et al., 2011; Watt et al., 2011; Jaynes et al., 2015; Xia et al., 2016, 2020; Zhang et al., 2019, 2020; X. Shi et al., 2022; L. Li et al., 2022, 2023). The modulation of the whistler-mode wave growth is potentially caused by compression-induced ambient thermal or resonant hot electron density variations (W. Li, Bortnik, Thorne, Nishimura, et al., 2011; Xia et al., 2016, 2020; Zhang et al., 2019, 2020), resonant electron anisotropy variations (W. Li, Thorne, et al., 2011; Watt et al., 2011), and nonlinear effects from periodic magnetic field configuration variations (L. Li et al., 2022, 2023). The periodic excitation of whistler-mode waves at the ULF wave frequency leads to periodic electron precipitation, which drives pulsating auroras (e.g., Miyoshi et al., 2010; Nishimura et al., 2010; Jaynes et al., 2015) and may account for many previously reported dTEC modulations at ULF frequencies (Pilipenko et al., 2014; Watson, Jayachandran, Singer, et al., 2016; Zhai et al., 2021).

However, it is challenging to establish a direct link between magnetospheric drivers and ionospheric TEC perturbations during ULF modulation events due to several factors: (1) the path-integrated nature of TEC perturbations, which strongly depend on the satellite-to-receiver raypath elevation (e.g., Jakowski et al., 1996; Komjathy, 1997), (2) inherent phase shifts due to complex propagation and modulation effects (Watson et al., 2015), particularly when conjugate observations are misaligned or not synchronized, and (3) the dynamic and turbulent nature of the auroral ionosphere (Kelley, 2009). Direct evidence linking TEC perturbations to magnetospheric drivers is yet to be discovered.

In this study, fortuitously conjugate observations from the THEMIS spacecraft and the GPS receiver at Fairbanks, Alaska (FAIR) allow us to identify the driver of GPS TEC perturbations as magnetospheric electron precipitation induced by ULF-modulated whistler-mode waves. Figure 1 illustrates the physical picture emerging from our conjugate magnetospheric and ionospheric observations of ULF waves, modulated whistler-mode waves, electron precipitation, and TEC perturbations. These results provide better specifications of high-latitude drivers for physics-based TEC modeling (Ridley et al., 2006; Zettergren & Snively, 2015; Meng et al., 2016, 2020; Sheng et al., 2020; Verkhoglyadova et al., 2020; Huba & Drob, 2017) and enhance forecasting capabilities by incorporating the effects of magnetospheric wave-driven electron precipitation.

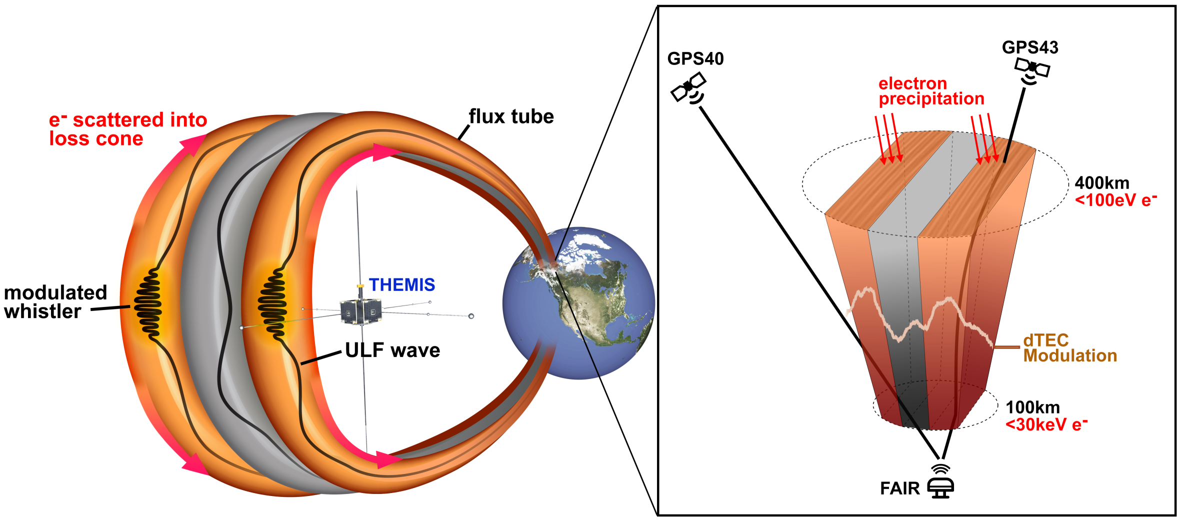

Figure 1. Schematic from THEMIS and FAIR observations of (a) modulation of magnetospheric precipitation due to electron scattering into the loss cone by ULF-modulated whistler-mode waves near the magnetic equator; and (b) the modulated electron precipitation has energies of ∼0.1–30 keV, depositing their energies at altitudes between ∼100–400 km, and inducing modulated impact ionization and TEC perturbations (dTEC) having amplitudes as large as ∼0.5 TECU and spanning scales of ∼5–80 km.

In what follows, Section 2 describes datasets and models employed to study whistler-driven precipitation and resulting TEC perturbations. Section 3 presents a detailed analysis and cross-correlation between observed and modeled TEC perturbations. Section 4 discusses the geophysical implications and applications of our results, which are followed by our main conclusions.

2 Data and Methodology

We derive 1-s vertical TEC (VTEC) measurements from phase and pseudorange data collected by the GPS receiver at Fairbanks, Alaska (FAIR) during 15:06–16:36 UT on July 3, 2013, processed at Jet Propulsion Laboratory using the GipsyX and Global Ionospheric Mapping (GIM) software (Komjathy et al., 2005; Bertiger et al., 2020). Phase-based TEC measurements are leveled using pseudorange delays for each phase-connected data collection. We focus on links between FAIR and GPS satellites with pseudo random noise (PRN) numbers 40, 43, and 60, referred to as GPS40, GPS43, and GPS60, whose ionospheric pierce points (IPP) at 450 km altitude are within 300 km proximity to FAIR, or IPPs at 150 km are within 100 km proximity to FAIR. The obtained TEC is expressed in TEC units (TECU), i.e., 10^{16} electrons/m^2. The slant TEC is converted to VTEC using the standard mapping function (e.g., Mannucci et al., 1998). Measurements with elevation angles less than 30° are not considered here. The VTEC data are then detrended to get TEC perturbations (dTEC) using a fourth-order Butterworth low-pass filter. We focus on dTEC with wave periods smaller than 25 min. The accuracy of dTEC based on phase measurements is ∼0.01–0.02 TECU (e.g., Coster et al., 2013).

We use the following datasets from THEMIS-E (Angelopoulos, 2008): electron energy and pitch-angle distributions measured by the Electrostatic Analyzers (ESA) instrument in the energy range of several eV up to 30 keV (McFadden et al., 2008), DC vector magnetic field at spin resolution (∼3 s) measured by the Fluxgate Magnetometers (FGM) (Auster et al., 2008), electric and magnetic field wave spectra within 1 Hz–4 kHz, measured every ∼8 s by the Digital Fields Board (DFB), the Electric Field Instrument (EFI), and the search coil magnetometer (SCM) (Le Contel et al., 2008; Bonnell et al., 2008; Cully, Ergun, et al., 2008). Background electron densities are inferred from spacecraft potentials (Bonnell et al., 2008; Nishimura et al., 2013). We also use ground-based magnetometer measurements every 1 s from the College (CMO) site operated by

the United States Geological Survey Geomagnetism Program and from the Fort Yukon (FYKN) site operated by the Geophysical Institute at the University of Alaska.

THEMIS observations of electron distributions and wave spectra allow us to calculate the precipitating flux of electrons scattered into the loss cone by whistler-mode waves using quasilinear diffusion theory (Kennel & Engelmann, 1966; Lyons, 1974). For whistler-mode wave normals θ <45°, we use a validated analytical formula of bounce-averaged electron diffusion coefficients from Artemyev et al. (2013). For small pitch angle α_{eq} approaching the loss cone α_{LC}, the first-order cyclotron resonance provides the main contribution to the bounce-averaged diffusion rate:

$$\langle D_{\alpha_{eq}\alpha_{eq}}\rangle \simeq \frac{\pi B_w^2 \Omega_{ceq}\omega_m}{4\gamma B_{eq}^2 \Delta\omega (p\epsilon_{meq})^{13/9} T(\alpha_{LC})\cos^2_{\alpha_{LC}}} \times \frac{\Delta\lambda_{R,N}(1+3\sin^2\lambda_R)^{7/12}(1-\bar{\omega})}{|\gamma\bar{\omega}-2\gamma\bar{\omega}^2+1||1-\gamma\bar{\omega}|^{4/9}}, \quad (1)$$

with B_w indicating the wave amplitude, ω_m the mean wave frequency, $\Delta\omega$ the frequency width, $\bar{\omega} = \omega_m/\Omega_{ce}$ the normalized frequency, Ω_{ce} and Ω_{ceq} the local and equatorial electron cyclotron frequency, γ the relativistic factor, p the electron momentum, $\epsilon_{meq} = \Omega_{pe}/\Omega_{ceq}\sqrt{\omega_m/\Omega_{ceq}}$ where Ω_{pe} is the plasma frequency, $T(\alpha_{eq})$ the bounce period, λ_R the latitude of resonance, and $\Delta\lambda_{R,N}$ the latitudinal range of resonance (see details in Artemyev et al. (2013)). The precipitating differential energy flux within the loss cone can be estimated as $x(E)J(E,\alpha_{LC})$, where

$$x(E) = 2\int_0^1 I_0(Z_0\tau)\tau d\tau / I_0(Z_0), \quad (2)$$

being the index of loss cone filling, $J(E,\alpha_{LC})$ is the electron differential energy flux near the loss cone, I_0 is the modified Bessel function with an argument $Z_0 \simeq \alpha_{LC}/\sqrt{\langle D_{\alpha_{eq}\alpha_{eq}}\rangle \cdot \tau_{loss}}$ (Kennel & Petschek, 1966), and τ_{loss} is assumed to be half of the bounce period.

With an energy distribution of precipitating electrons within 0.1–30 keV, we estimate the impact ionization rate altitude profile using the parameterization model developed by Fang et al. (2010), covering isotropic electron precipitation from 100 eV up to 1 MeV. This model, derived through fits to first-principle model results, allows efficient ionization computation for arbitrary energy spectra. Atmospheric density and scale height data were obtained from the NRLMSISE-00 model (Picone et al., 2002). We model TEC perturbations resulting from whistler-induced electron precipitation by integrating ionization rates over altitude and time, adopting an 8-s integration period to align with the temporal resolution of THEMIS wave spectra data. Although our analysis does not concern equilibrium densities and omits recombination and convective effects, this

neglect has little impact because we focus on relative TEC perturbations due to short-time precipitation. It takes nearly 60 s for the background ionosphere to relax to an equilibrium density solution for 10-keV precipitation and longer for lower energies (e.g., Kaeppler et al., 2022). Our estimated dTEC also closely match observed dTEC values, underscoring the effectiveness of our modeling approach despite its approximation.

3 Results

On July 3, 2013, from 15:06 to 16:36 UT, the THEMIS-E spacecraft flew westward over the FAIR GPS receiver station, coming within ∼20 km relative to FAIR when mapped to 450 km altitude. This optimally timed and positioned space-ground conjunction offers a unique opportunity to link between magnetospheric and ionospheric processes along the field line. The event occurred at $L \sim 7$, outside the plasmapause of $L_{pp} \sim 5.4$ (based on THEMIS-E densities near 17:00 UT), near the magnetic local time (MLT) of 4.5 hr, and during a geomagnetic quiet time with $Kp \sim 1$ and $AE \sim 200$ nT. Figure 2a illustrates the trajectories of THEMIS-E and the ionosphere pierce points (IPPs) of GPS40, GPS43, and GPS60 near FAIR, mapped to 450 km altitude. The footprints of THEMIS-E are field-line traced using the Tsyganenko T96 model (Tsyganenko, 1995) but the GPS satellites are mapped using line of sight. Of these GPS satellites, the GPS43 IPPs, moving eastward, were nearest to both the FAIR and THEMIS-E footprints, exhibiting close longitudinal alignment. A notable conjugacy, marked by the bright red segment from 15:37 to 16:11 UT, occurred when the footprints of THEMIS-E and GPS43 IPPs were within ∼100 km to each other and FAIR (Figure 2j). In Supporting Information, we also present the configuration when the satellites and their IPPs are mapped to an altitude of 150 km. This adjustment does not significantly alter the geometry of our conjunction event, but it does slightly reduce the scale of the satellite footpaths near FAIR.

Figures 2b–2d present THEMIS observations of whistler-mode waves. The observed wave frequencies were in the whistler lower band, spanning ~ 0.2–$0.5\Omega_{ce}$, with a mean frequency $\omega_m \sim 0.35\Omega_{ce}$, and $\Delta\omega \sim 0.15\Omega_{ce}$, where the electron cyclotron frequency $f_{ce} \sim \Omega_{ce}/2\pi \sim 2.15$ kHz. Figure 2d shows that whistler-mode wave amplitudes B_w range from several pT to over 100 pT, measured at 8-s cadence (black curve) and smoothed with 2-min moving averages (red curve). Short-term oscillations in B_w on the order of tens of seconds were observed atop more gradual variations of several minutes. We mainly use smoothed or average B_w to estimate electron precipitation in the following. Absent

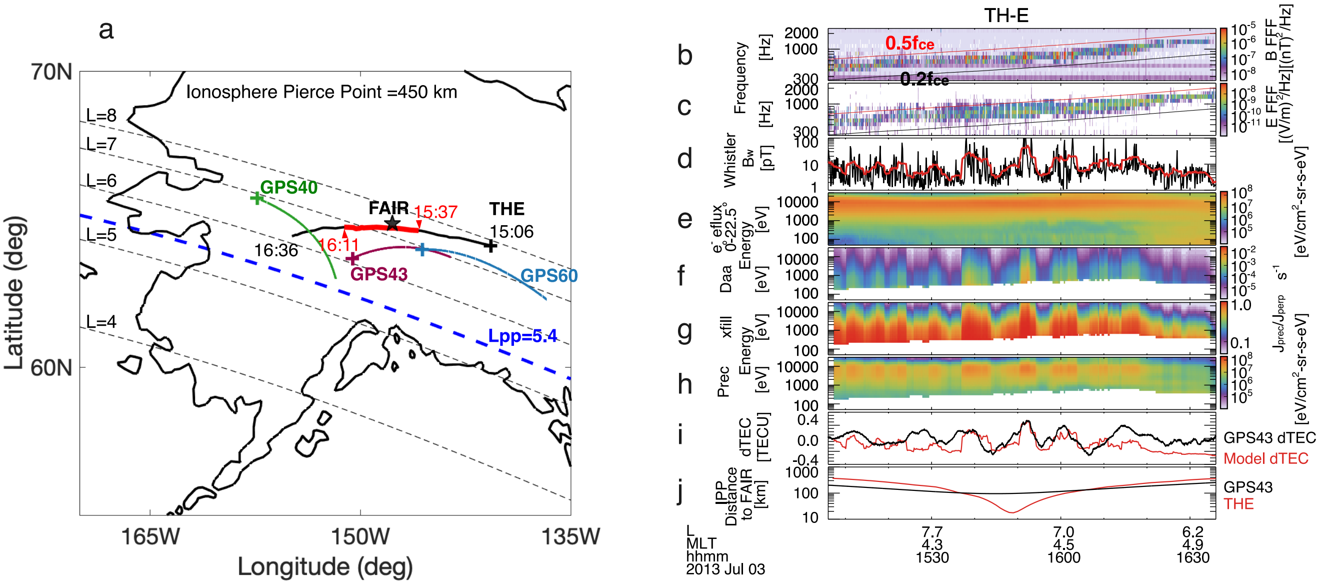

Figure 2. (a) Configuration of THEMIS-E (black curve), GPS40, GPS43, and GPS60 satellites (green, purple, and blue curves), and the FAIR receiver (black star) in geographic coordinates, with THEMIS and GPS mapped onto 450 km altitude using T96 field tracing (THEMIS) or line of sight (GPS). The plus symbol indicates the start of the footpath. (b–e) THEMIS-E magnetic field spectrogram, electric field spectrogram, whistler-mode wave amplitudes, and field-aligned (0°–22.5°) electron energy spectrogram. (f) Bounce-averaged electron diffusion rates. (g) Index of loss cone filling. (h) Whistler-driven precipitating electron energy spectrogram. (i) Comparison of whistler-driven model dTEC (red curve) and GPS43-observed dTEC (black curve). (j) Great-circle distances between THEMIS-E footpath (red curve) and GPS43 raypath (black curve) at IPP of 450 km relative to the FAIR station.

direct waveform data on whistler-mode wave normals for our event, we infer, based on the measured E/cB spectra ($E/cB \ll 1$, see Supporting Information) and statistical night-side equatorial plasma sheet observations (W. Li, Bortnik, Thorne, & Angelopoulos, 2011; Agapitov et al., 2013; Meredith et al., 2021), the presence of quasi-parallel whistlers with an assumed Gaussian wave normal width of $\Delta\theta \sim 30°$ confined within $\pm 30°$ in latitude.

Figures 2e–2h display the measured plasma sheet field-aligned ($\alpha \sim [0°, 22.5°]$) electrons from 50 eV up to 25 keV, calculated diffusion rates $\langle D_{\alpha_{eq}\alpha_{eq}} \rangle$, estimated loss cone filling $x(E)$, and precipitating electron energy fluxes. Although $\langle D_{\alpha_{eq}\alpha_{eq}} \rangle$ and $x(E)$ increase at lower energies, the precipitating energy fluxes peak between 1-10 keV, exhibiting similar modulations as seen in the smoothed whistler-mode wave amplitude B_w. Electron precipitation fluxes below ~200 eV are absent due to an energy threshold for electron cyclotron resonance interaction, with the lower limit primarily determined by the ratio Ω_{pe}/Ω_{ce} (~3 in our case).

Figure 2i compares modeled (red) and directly measured dTEC (black) from the GPS43 signal, revealing a nearly one-to-one phase correlation from 15:37 to 16:11 UT. This period of close correlation coincides with the conjunction of THEMIS-E, GPS43, and FAIR, where their relative distances were within ~100 km (Figure 2j). Outside this conjugacy period and further away from the FAIR station, the correlation decreases. Observed peak-to-peak amplitudes of dTEC reached ~0.5 TECU. Note that this particular event occurred during quiet conditions; other events during storms may have much larger TEC modulation amplitudes (e.g., Watson et al., 2015), though more challenging to have such reliable conjunction, especially given uncertainties in magnetic field mapping during storms (e.g., C.-L. Huang et al., 2008).

Figure 3 underscores the critical role of observation geometry and timing in detecting phase correlations between modeled and measured dTEC across three GPS satellites. Despite all three satellites having raypath elevation angles >40°—reducing the likelihood of multi-path effects (e.g., Kintner et al., 2007)—only the GPS43 elevation reached 80° above the FAIR station zenith (Figure 3a). During the conjugacy period, the IPPs of GPS40 and GPS60 were distanced from FAIR by more than 200 km, while GPS43's IPPs remained within 100 km, coming within 20 km at its closest point (Figure 3b). Figures 3c and 3d reveal that the modeled dTEC (red curve) aligns poorly with GPS40 and GPS60 dTEC (blue and magenta curves), but a significant cross-correlation (~0.8) emerges with

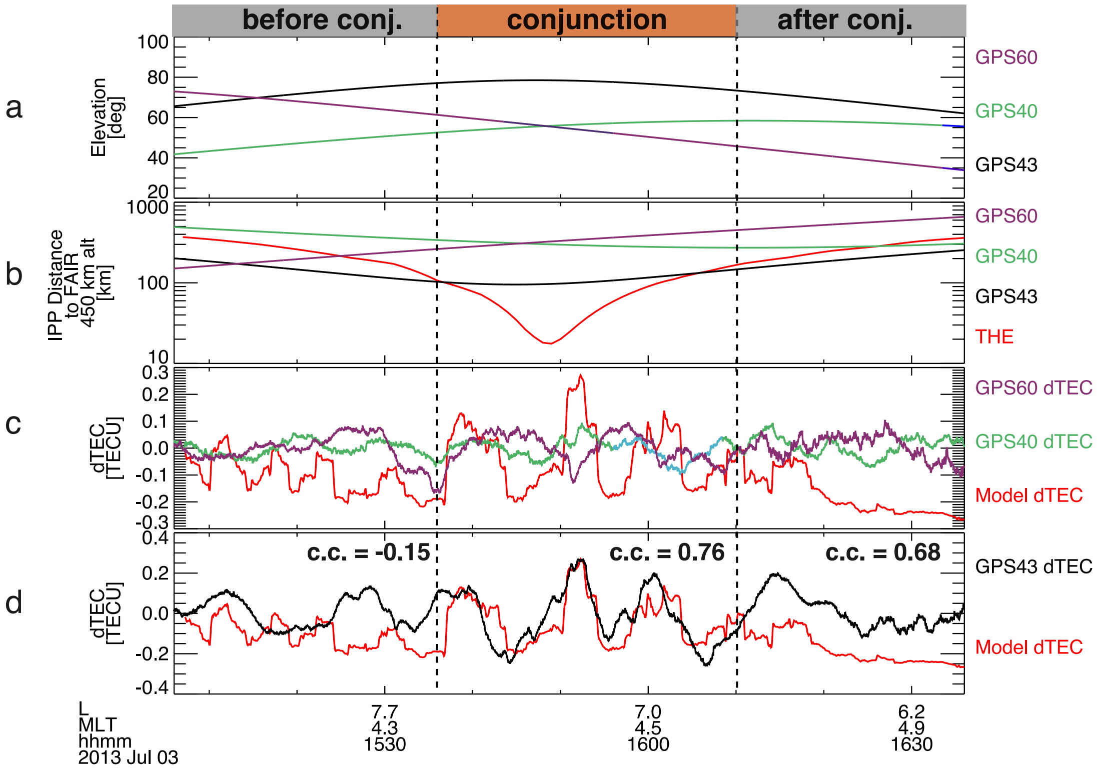

Figure 3. (a) Raypath elevation angles of GPS40 (green curve), GPS43 (black curve), and GPS60 (magenta curve). (b) Distances between THEMIS-E footpath and GPS satellite IPPs relative to FAIR, displayed in the same format as Figure 1j. (c) Comparison between whistler-driven model dTEC and observed dTEC from GPS40 and GPS60, which were not in good conjunction with THEMIS or FAIR. (d) Comparison between whistler-driven model dTEC and GPS43-observed dTEC. The cross-correlation coefficients are -0.15, 0.76, and 0.68 during intervals before, during, and after conjunction, respectively.

GPS43 dTEC (black) during the conjugacy period. Before and after the conjunction, TEC phase shifts reduce the cross-correlation to -0.15 and 0.68, respectively. Given the near-parallel longitudinal alignment of GPS43 IPPs and THEMIS-E footprints (Figure 2a), the measured dTEC (black) potentially reflects both temporal and spatial/longitudinal modulations. These findings suggest that to reliably identify the electron precipitation responsible for TEC perturbations requires precise spacecraft spatial alignment, optimal timing, and high raypath elevations.

The modulation of TEC perturbations, electron precipitation, and whistler-mode wave amplitudes was linked to ULF wave activities in the Pc3-5 band (1.7 mHz to 100 mHz). Figure 4a display the magnetic field perturbations measured by THEMIS-E in the mean field-aligned (MFA) coordinates, in which the parallel direction (||, the compressional component) is determined by 15-minute sliding averages of the magnetic field, the azimuthal direction (ϕ, the toroidal component) is along the cross product of z and the spacecraft geocentric position vector, and the radial direction (r, the poloidal component) completes the triad. Magnetic perturbations are obtained by subtracting the 15-minute mean field. During the conjunction, THEMIS-E detected both compressional Pc5 waves (red curve) and poloidal Pc3-4 waves (blue curve). Figure 4b indicates that peaks in whistler-mode wave amplitudes approximately align with troughs of compressional ULF waves, with fine-scale whistler amplitudes primarily modulated by poloidal Pc3-4 waves (See Supporting Information). Strong Pc5 ULF waves were also recorded in the H-component magnetic field perturbations from magnetometers located at CMO and FYKN (Figures 4g–4h), displaying a similar pattern but with greater amplitudes at FYKN, located slightly north of FAIR. The discrepancy between ground- and space-measured Pc5 waves potentially results from the localized nature of THEMIS-E observations (X. Shi et al., 2022) and the screening/modification effects of ULF waves traversing the ionosphere (Hughes & Southwood, 1976; Lysak, 1991; Lessard & Knudsen, 2001; X. Shi et al., 2018). These observations imply that the ionospheric TEC perturbations were linked to ULF-modulated whistler-mode waves and the associated electron precipitation (e.g., Coroniti & Kennel, 1970; W. Li, Thorne, et al., 2011; Xia et al., 2016; Zhang et al., 2020; L. Li et al., 2023).

Figures 4b–4c compare small-scale/high-frequency fluctuations of whistler-mode wave amplitudes B_w and dTEC, which was bandpass-filtered within the frequency range of 5–200 mHz. The small-scale dTEC fluctuations exhibit similar wave periods to B_w

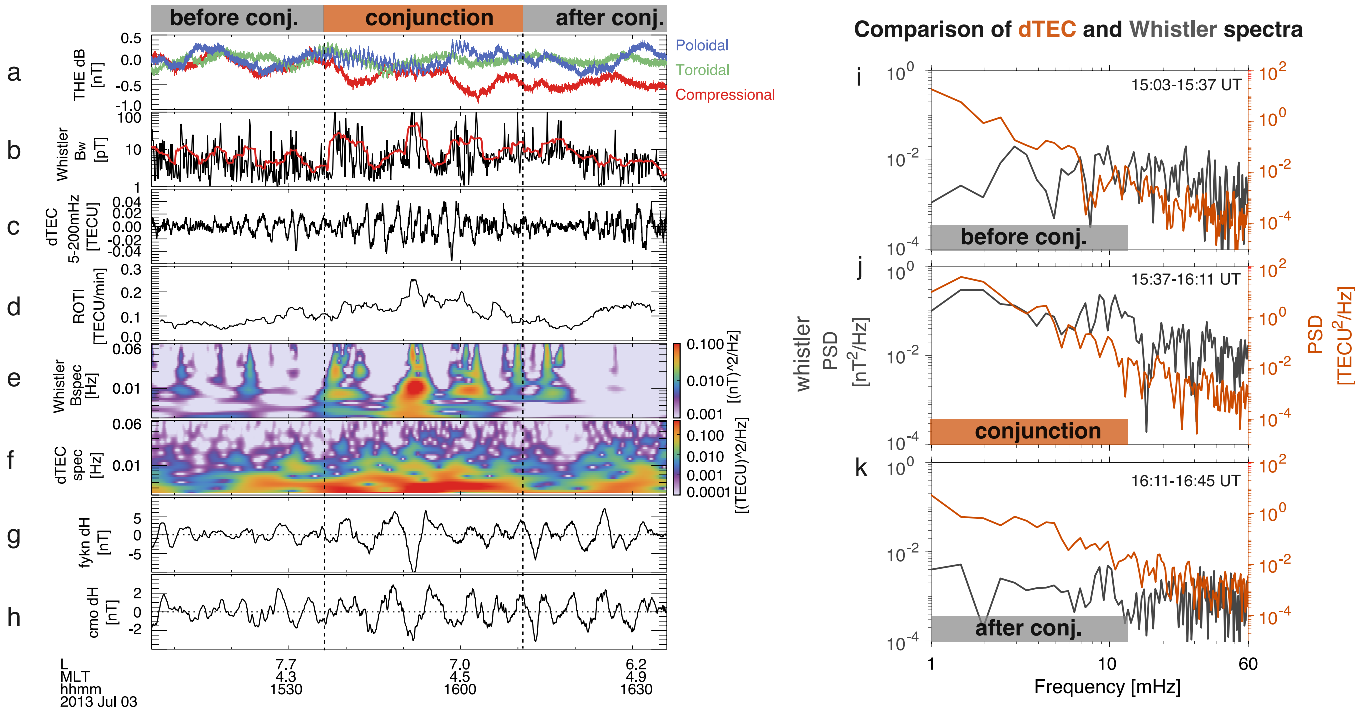

Figure 4. (a) THEMIS-E magnetic field perturbations in the mean-field-aligned (MFA) coordinates, exhibiting primarily compressional- (red) and poloidal-mode (blue) variations. (b) THEMIS-E whistler-mode wave amplitudes. The measured amplitudes are shown in black and smoothed in red. (c) dTEC bandpass filtered within 5–200 mHz. (d) The rate of dTEC changes ($ROTI$) from 200-s sliding window ensemble averaging. (e) Wavelet spectrogram of whistler-mode waves. (f) Wavelet spectrogram of GPS43 dTEC. (g) Ground-based magnetic field H component perturbations in 1.7–100 mHz from the Fort Yukon station (FYKN). (h) Ground-based magnetic H component perturbations in 1.7–100 mHz from the College station (CMO). (i–k) Comparisons of dTEC (orange curves) and whistler-mode wave amplitude fluctuation spectra (gray curves) in 1–60 mHz measured before (k), during (j), and after (k) the conjugacy period.

fluctuations, evidently intensifying during the conjugacy period, yet lacking a clear phase correlation seen with larger scale perturbations in Figure 3d. Assumed purely spatial, small-scale dTEC variations have wavelengths of ∼15–30 km at IPPs of 450 km altitude (or ∼5–10 km at IPPs of 150 km altitude), compared with the larger-scale dTEC wavelengths of ∼80 km (or ∼25 km at IPPs of 150 km altitude). When mapped to the magnetosphere, these small-scale dTEC modulations correspond to a magnetospheric source region of ∼100–700 km, while large-scale dTEC modulations suggest a source region of ∼500–2000 km. These scales align with prior observations of the transverse scale sizes of chorus elements and their source regions (SantolíK et al., 2003; Agapitov et al., 2017, 2018), and with the azimuthal wavelengths of high-m poloidal ULF waves (Yeoman et al., 2012; X. Shi et al., 2018; Zong et al., 2017). Figure 4d shows the rate of TEC index ($ROTI$), i.e., the standard deviation of the rate of TEC (ROT) (Pi et al., 1997), where $ROT = (dTEC(t+\tau) - dTEC(t))/\tau$ with τ =10 s, $ROTI = \sqrt{\langle ROT^2 \rangle - \langle ROT \rangle^2}$ using 200-s sliding averages. Significant increases in $ROTI$ were observed within the region of whistler-driven TEC perturbations. However, in our case the GPS signal fluctuations were predominantly refractive, as negligible fluctuations were detected at frequencies above 0.1 Hz (McCaffrey & Jayachandran, 2017, 2019; Nishimura et al., 2023).

Figures 4e–4f compare the wavelet spectrograms of whistler-mode wave B_w and dTEC, displaying concurrent increases in wave power for both in the frequency range of ∼3 mHz up to tens of mHz. Figures 4i–4k present a more detailed amplitude spectra comparison before, during, and after conjunction. Notably, only during the conjunction, whistler-mode wave amplitudes and dTEC share similar power spectral density (PSD) distributions in the 1–∼30 mHz range. The peaks in whistler spectra were slightly and consistently larger than those in dTEC spectra within 3–20 mHz by factors of 1.05–1.2, aligning with expected Doppler shift effects on ionospheric TEC measurements. The Doppler shift results from relative motion of GPS raypath (or IPPs with velocities of ∼160 m/s in our case) and propagating TEC structures (typically with velocities of several hundred m/s) (Watson, Jayachandran, & MacDougall, 2016): $f_{cor} = f_{obs}(1+\frac{\mathbf{v_{ipp}} \cdot \mathbf{v_{struct}}}{|\mathbf{v_{struct}}|^2})$, where f_{cor} is the frequency corrected for relative motion. Watson, Jayachandran, and MacDougall (2016) found that 89% of their statistical events required a correction factor of 1.2 or less for the Doppler shift, consistent with our observations. The agreement between dTEC and whistler amplitude spectra supports that the observed dTEC resulted from electron precipitation induced by whistler-mode waves.

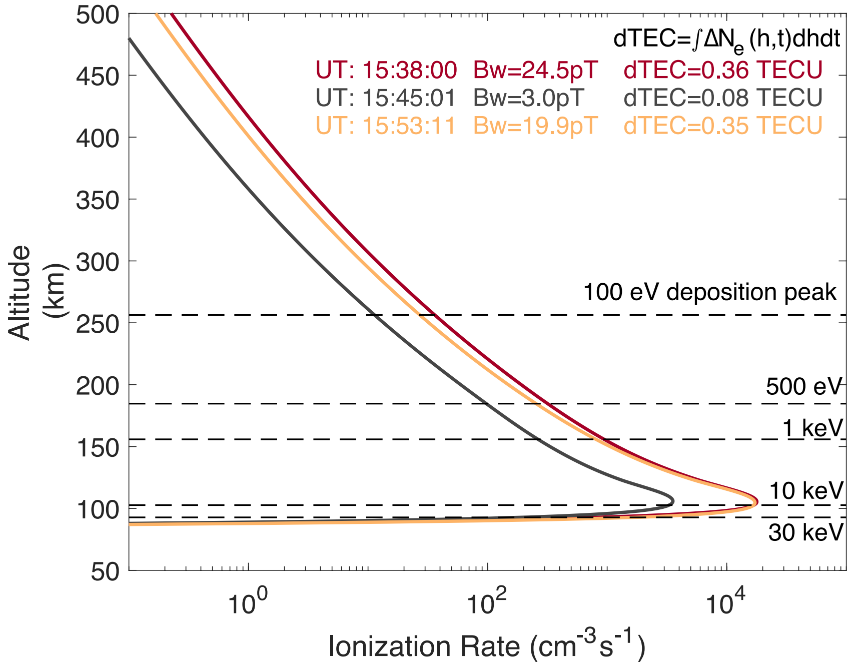

Figure 5. Ionization rate altitude profiles calculated at three time stamps of 15:38:00, 15:45:01, and 15:53:11 UT, corresponding to whistler-mode wave amplitudes of B_w= 24.5 pT (red curve), 3.0 pT (gray curve), and 19.9 pT (orange curve). The dTEC were calculated by integrating ionization rates over altitude and time (8s). The dashed lines mark the peak deposition altitudes of 100 eV, 500 eV, 1 keV, 10 keV, and 30 keV precipitating monoenergetic electrons.

Figure 5 indicates that the electron precipitation, induced by ULF-modulated whistler-mode waves, can cause significant increases in ionospheric ionization rate or column density, leading to TEC perturbations of ∼0.36 TECU with a moderate whistler amplitude of B_w ∼25 pT. Given that large-amplitude whistler-mode waves exceeding several hundred pT frequently occur in the inner magnetosphere (Cattell et al., 2008; Cully, Bonnell, & Ergun, 2008; Agapitov et al., 2014; Hartley et al., 2016; R. Shi et al., 2019), we anticipate even larger TEC perturbations from such whistler activities. We defer a statistical study including storm time events and the potential connection with scintillation (e.g., McCaffrey & Jayachandran, 2019; Nishimura et al., 2023) for the future. In addition, the primary energy range of precipitation spans from ∼100 eV to ∼30 keV, contributing to density variations between ∼90–∼400 km (Fang et al., 2010; Katoh et al., 2023; Berland et al., 2023).

4 Discussion

Various mechanisms have been proposed that link ULF waves to TEC perturbations and ionospheric disturbances in general (Pilipenko et al., 2014). Although TEC perturbations might arise from direct ULF wave effects through convective and divergent flows, MHD Alfvén-mode waves do not directly alter plasma density. Furthermore, mode-converted compressional waves, if present due to Hall currents, are evanescent in the ionosphere (Lessard & Knudsen, 2001), resulting in minimal TEC perturbations (Pilipenko et al., 2014). A non-linear ”feedback instability” mechanism may modify ULF wave dynamics, causing field-aligned current striations and significant bottom-side ionospheric density cavities and gradients (Lysak, 1991; Streltsov & Lotko, 2008). Additionally, electron precipitation and Joule heating are important factors to consider in the auroral region (e.g., Deng & Ridley, 2007; Sheng et al., 2020; Meng et al., 2022).

Detecting one-to-one phase correlation between ground-based ULF waves and TEC perturbations may be challenging, largely due to ionospheric screening effects on ULF waves (Hughes & Southwood, 1976), with only a few exceptions noted during storm times (Pilipenko et al., 2014; Wang et al., 2020). However, this correlation has been frequently observed with spacecraft measurements of ULF waves (Watson et al., 2015; Watson, Jayachandran, Singer, et al., 2016; Zhai et al., 2021), indicating that magnetospheric processes may play an important role in driving ionospheric TEC perturbations. Our findings support that magnetospheric whistler-mode waves, modulated by ULF waves in the

Pc3–5 band, are responsible for these periodic TEC perturbations through associated electron precipitation.

These results enhance our understanding of TEC modulation by ULF waves, a topic widely discussed in the literature (Skone, 2009; Pilipenko et al., 2014; Watson et al., 2015; Watson, Jayachandran, Singer, et al., 2016; Wang et al., 2020; Zhai et al., 2021), and facilitates the integration of effects of magnetospheric whistler-mode waves into auroral TEC models. Statistical modeling of whistler-mode and ULF waves has been improving for several decades (e.g., Tsurutani & Smith, 1974; McPherron, 1972; Takahashi & Anderson, 1992; W. Li, Bortnik, Thorne, & Angelopoulos, 2011; Agapitov et al., 2013; Artemyev et al., 2016; Tyler et al., 2019; Zong et al., 2017; Ma et al., 2020; Zhang et al., 2020; Sandhu et al., 2021; Hartinger et al., 2015, 2022, 2023). Leveraging these wave effects and the associated electron precipitation can enhance understanding and physics-based modeling of high-latitude TEC (Ridley et al., 2006; Zettergren & Snively, 2015; Meng et al., 2016, 2020; Sheng et al., 2020; Verkhoglyadova et al., 2020; Huba & Drob, 2017). Incorporating these magnetospheric phenomena is vital for improving the accuracy of ionospheric TEC models during periods of elevated geomagnetic activities, potentially benefiting ionospheric science and GNSS-based applications.

5 Conclusions

We present a detailed case study of ionospheric TEC perturbations (dTEC), using fortuitously timed and positioned conjugate observations from the THEMIS spacecraft and the GPS receiver at Fairbanks, Alaska (FAIR). This conjunction setup allows us to identify the magnetospheric driver of the observed dTEC. Our key findings are summarized below:

- Combining in-situ wave and electron observations and quasilinear theory, we have modeled the electron precipitation induced by observed whistler-mode waves and deduced ionospheric dTEC based on impact ionization predictions. The cross-correlation between the modeled and observed dTEC reached ∼0.8 during the conjugacy period of ∼30 min but decreased outside of it.
- Observed peak-to-peak dTEC amplitudes reached ∼0.5 TECU, exhibiting modulations spanning scales of ∼5–80 km. Within the modulated dTEC, enhancements in the rate of TEC index (ROTI) were measured to be ∼0.2 TECU/min.

- The whistler-mode waves and dTEC modulations were linked to ULF waves in the Pc3-5 band, featuring concurrent compressional and poloidal mode fluctuations. The amplitude spectra of whistler-mode waves and dTEC also agreed from 1 mHz to tens of mHz during the conjugacy period but diverged outside of it.

Thus, our results provide direct evidence that ULF-modulated whistler-mode waves in the magnetosphere drive electron precipitation leading to ionospheric TEC perturbation modulations. Our observations also indicate that to reliably identify the electron precipitation responsible for TEC perturbations requires precise spacecraft spatial alignment, optimal timing, and high raypath elevations. Our findings may help to augment physics-based high-latitude TEC modeling and forecasting by incorporating the effects of magnetospheric wave-driven precipitation.

Acknowledgments

This work has been supported by NASA projects 80NSSC23K0413, 80NSSC24K0138, and 80NSSC23K1038. Portions of the research were carried out at the Jet Propulsion Laboratory, California Institute of Technology, under a contract with NASA. O.P.V., M.D.H., and X.S. were supported by NASA project 80NSSC21K1683. X.S. is supported by NASA award 80NSSC21K1677. O.P.V would like to thank A.W. Moore (JPL) for data processing discussions. We are indebted to Emmanuel Masongsong for help with the schematic figure.

Open Research

THEMIS data are available at `http://themis.ssl.berkeley.edu/data/themis/`. GPS RINEX data are publicly available from the NASA CDDIS archive of space geodesy data (`https://cddis.nasa.gov/Data_and_Derived_Products/index.html`). TEC data derived for this study is available at `https://dataverse.jpl.nasa.gov/dataset.xhtml?persistentId=doi:10.48577/jpl.KXY5BW`. Original CMO data are provided by the USGS Geomagnetism Program (`http://geomag.usgs.gov`). FYKN data are part of the Geophysical Institute Magnetometer Array operated by the Geophysical Institute, University of Alaska (`http://magnet.asf.alaska.edu/`). Data access and processing was done using SPEDAS V4.1, see Angelopoulos et al. (2019).

References

Aa, E., Zou, S., Eastes, R., Karan, D. K., Zhang, S.-R., Erickson, P. J., & Coster, A. J. (2020, January). Coordinated Ground-Based and Space-Based Observations of Equatorial Plasma Bubbles. *Journal of Geophysical Research (Space Physics)*, *125*(1), e27569. doi: 10.1029/2019JA027569

Agapitov, O. V., Artemyev, A., Krasnoselskikh, V., Khotyaintsev, Y. V., Mourenas, D., Breuillard, H., ... Rolland, G. (2013, June). Statistics of whistler mode waves in the outer radiation belt: Cluster STAFF-SA measurements. *J. Geophys. Res.*, *118*, 3407-3420. doi: 10.1002/jgra.50312

Agapitov, O. V., Artemyev, A., Mourenas, D., Krasnoselskikh, V., Bonnell, J., Le Contel, O., ... Angelopoulos, V. (2014). The quasi-electrostatic mode of chorus waves and electron nonlinear acceleration. *J. Geophys. Res.*, *119*, 1606–1626. doi: 10.1002/2013JA019223

Agapitov, O. V., Blum, L. W., Mozer, F. S., Bonnell, J. W., & Wygant, J. (2017, March). Chorus whistler wave source scales as determined from multipoint Van Allen Probe measurements. *Geophys. Res. Lett.*, *44*, 2634-2642. doi: 10.1002/2017GL072701

Agapitov, O. V., Mourenas, D., Artemyev, A., Mozer, F. S., Bonnell, J. W., Angelopoulos, V., ... Krasnoselskikh, V. (2018, October). Spatial Extent and Temporal Correlation of Chorus and Hiss: Statistical Results From Multipoint THEMIS Observations. *Journal of Geophysical Research (Space Physics)*, *123*(10), 8317-8330. doi: 10.1029/2018JA025725

Angelopoulos, V. (2008, December). The THEMIS Mission. *Space Sci. Rev.*, *141*, 5-34. doi: 10.1007/s11214-008-9336-1

Angelopoulos, V., Cruce, P., Drozdov, A., Grimes, E. W., Hatzigeorgiu, N., King, D. A., ... Schroeder, P. (2019, January). The Space Physics Environment Data Analysis System (SPEDAS). *Space Sci. Rev.*, *215*, 9. doi: 10.1007/s11214-018-0576-4

Artemyev, A. V., Agapitov, O., Mourenas, D., Krasnoselskikh, V., Shastun, V., & Mozer, F. (2016, April). Oblique Whistler-Mode Waves in the Earth's Inner Magnetosphere: Energy Distribution, Origins, and Role in Radiation Belt Dynamics. *Space Sci. Rev.*, *200*(1-4), 261-355. doi: 10.1007/s11214-016-0252-5

Artemyev, A. V., Mourenas, D., Agapitov, O. V., & Krasnoselskikh, V. V. (2013,

April). Parametric validations of analytical lifetime estimates for radiation belt electron diffusion by whistler waves. *Annales Geophysicae*, *31*, 599-624. doi: 10.5194/angeo-31-599-2013

Astafyeva, E. (2019, December). Ionospheric Detection of Natural Hazards. *Reviews of Geophysics*, *57*(4), 1265-1288. doi: 10.1029/2019RG000668

Auster, H. U., Glassmeier, K. H., Magnes, W., Aydogar, O., Baumjohann, W., Constantinescu, D., ... Wiedemann, M. (2008, December). The THEMIS Fluxgate Magnetometer. *Space Sci. Rev.*, *141*, 235-264. doi: 10.1007/s11214-008-9365-9

Basu, S., Groves, K. M., Basu, S., & Sultan, P. J. (2002, November). Specification and forecasting of scintillations in communication/navigation links: current status and future plans. *Journal of Atmospheric and Solar-Terrestrial Physics*, *64*(16), 1745-1754. doi: 10.1016/S1364-6826(02)00124-4

Basu, S., MacKenzie, E., Basu, S., Carlson, H. C., Hardy, D. A., Rich, F. J., & Livingston, R. C. (1983, December). Coordinated measurements of low-energy electron precipitation and scintillations/TEC in the auroral oval. *Radio Science*, *18*(6), 1151-1165. doi: 10.1029/RS018i006p01151

Basu, S., MacKenzie, E., Basu, S., Coley, W. R., Sharber, J. R., & Hoegy, W. R. (1990, June). Plasma structuring by the gradient drift instability at high latitudes and comparison with velocity shear driven processes. *J. Geophys. Res.*, *95*(A6), 7799-7818. doi: 10.1029/JA095iA06p07799

Berland, G. D., Marshall, R. A., Capannolo, L., McCarthy, M. P., & Zheng, L. (2023, November). Kinetic Modeling of Radiation Belt Electrons With Geant4 to Study Energetic Particle Precipitation in Earth's Atmosphere. *Earth and Space Science*, *10*(11), e2023EA002987. doi: 10.1029/2023EA002987

Bertiger, W., Bar-Sever, Y., Dorsey, A., Haines, B., Harvey, N., Hemberger, D., ... Willis, P. (2020, August). GipsyX/RTGx, a new tool set for space geodetic operations and research. *Advances in Space Research*, *66*(3), 469-489. doi: 10.1016/j.asr.2020.04.015

Bonnell, J. W., Mozer, F. S., Delory, G. T., Hull, A. J., Ergun, R. E., Cully, C. M., ... Harvey, P. R. (2008, December). The Electric Field Instrument (EFI) for THEMIS. *Space Sci. Rev.*, *141*, 303-341. doi: 10.1007/s11214-008-9469-2

Cattell, C., Wygant, J. R., Goetz, K., Kersten, K., Kellogg, P. J., von Rosenvinge,

T., ... Russell, C. T. (2008, January). Discovery of very large amplitude whistler-mode waves in Earth's radiation belts. *Geophys. Res. Lett.*, *35*, 1105. doi: 10.1029/2007GL032009

Chaston, C. C., Peticolas, L. M., Bonnell, J. W., Carlson, C. W., Ergun, R. E., McFadden, J. P., & Strangeway, R. J. (2003, February). Width and brightness of auroral arcs driven by inertial Alfven waves. *Journal of Geophysical Research (Space Physics)*, *108*(A2), 1091. doi: 10.1029/2001JA007537

Ciraolo, L., Azpilicueta, F., Brunini, C., Meza, A., & Radicella, S. M. (2007). Calibration errors on experimental slant total electron content (TEC) determined with GPS. *Journal of Geodesy*, *81*, 111–120.

Coker, C., Hunsucker, R., & Lott, G. (1995, December). Detection of auroral activity using GPS satellites. *Geophys. Res. Lett.*, *22*(23), 3259-3262. doi: 10.1029/95GL03091

Coroniti, F. V., & Kennel, C. F. (1970, March). Electron precipitation pulsations. *J. Geophys. Res.*, *75*(7), 1279-1289. doi: 10.1029/JA075i007p01279

Coster, A., Williams, J., Weatherwax, A., Rideout, W., & Herne, D. (2013, March). Accuracy of GPS total electron content: GPS receiver bias temperature dependence. *Radio Science*, *48*(2), 190-196. doi: 10.1002/rds.20011

Cully, C. M., Bonnell, J. W., & Ergun, R. E. (2008, June). THEMIS observations of long-lived regions of large-amplitude whistler waves in the inner magnetosphere. *Geophys. Res. Lett.*, *35*, 17. doi: 10.1029/2008GL033643

Cully, C. M., Ergun, R. E., Stevens, K., Nammari, A., & Westfall, J. (2008, December). The THEMIS Digital Fields Board. *Space Sci. Rev.*, *141*, 343-355. doi: 10.1007/s11214-008-9417-1

Davies, K., & Hartmann, G. K. (1976, July). Short-period fluctuations in total columnar electron content. *J. Geophys. Res.*, *81*(19), 3431. doi: 10.1029/JA081i019p03431

Deng, Y., & Ridley, A. J. (2007, September). Possible reasons for underestimating Joule heating in global models: E field variability, spatial resolution, and vertical velocity. *Journal of Geophysical Research (Space Physics)*, *112*(A9), A09308. doi: 10.1029/2006JA012006

Fæhn Follestad, A., Herlingshaw, K., Ghadjari, H., Knudsen, D. J., McWilliams, K. A., Moen, J. I., ... Oksavik, K. (2020, June). Dayside Field-Aligned

Current Impacts on Ionospheric Irregularities. *Geophys. Res. Lett.*, *47*(11), e86722. doi: 10.1029/2019GL086722

Fang, X., Randall, C. E., Lummerzheim, D., Wang, W., Lu, G., Solomon, S. C., & Frahm, R. A. (2010, November). Parameterization of monoenergetic electron impact ionization. *Geophys. Res. Lett.*, *37*(22), L22106. doi: 10.1029/2010GL045406

Fejer, B. G., & Kelley, M. C. (1980, May). Ionospheric irregularities. *Reviews of Geophysics and Space Physics*, *18*, 401-454. doi: 10.1029/RG018i002p00401

Hartinger, M. D., Elsden, T., Archer, M. O., Takahashi, K., Wright, A. N., Artemyev, A., ... Angelopoulos, V. (2023, December). Properties of Magnetohydrodynamic Normal Modes in the Earth's Magnetosphere. *Journal of Geophysical Research (Space Physics)*, *128*(12), e2023JA031987. doi: 10.1029/2023JA031987

Hartinger, M. D., Moldwin, M. B., Zou, S., Bonnell, J. W., & Angelopoulos, V. (2015, January). ULF wave electromagnetic energy flux into the ionosphere: Joule heating implications. *Journal of Geophysical Research (Space Physics)*, *120*(1), 494-510. doi: 10.1002/2014JA020129

Hartinger, M. D., Takahashi, K., Drozdov, A. Y., Shi, X., Usanova, M. E., & Kress, B. (2022, April). ULF Wave Modeling, Effects, and Applications: Accomplishments, Recent Advances, and Future. *Frontiers in Astronomy and Space Sciences*, *9*, 867394. doi: 10.3389/fspas.2022.867394

Hartley, D. P., Kletzing, C. A., Kurth, W. S., Bounds, S. R., Averkamp, T. F., Hospodarsky, G. B., ... Watt, C. E. J. (2016, May). Using the cold plasma dispersion relation and whistler mode waves to quantify the antenna sheath impedance of the Van Allen Probes EFW instrument. *Journal of Geophysical Research (Space Physics)*, *121*(5), 4590-4606. doi: 10.1002/2016JA022501

Hey, J. S., Parsons, S. J., & Phillips, J. W. (1946, August). Fluctuations in Cosmic Radiation at Radio-Frequencies. *Nature*, *158*(4007), 234. doi: 10.1038/158234a0

Huang, C.-L., Spence, H. E., Singer, H. J., & Tsyganenko, N. A. (2008, April). A quantitative assessment of empirical magnetic field models at geosynchronous orbit during magnetic storms. *Journal of Geophysical Research (Space Physics)*, *113*(A4), A04208. doi: 10.1029/2007JA012623

Huang, C.-S., & Kelley, M. C. (1996, January). Nonlinear evolution of equatorial spread F. 2. Gravity wave seeding of Rayleigh-Taylor instability. *J. Geophys. Res.*, *101*(A1), 293-302. doi: 10.1029/95JA02210

Huba, J. D., & Drob, D. (2017, June). SAMI3 prediction of the impact of the 21 August 2017 total solar eclipse on the ionosphere/plasmasphere system. *Geophys. Res. Lett.*, *44*(12), 5928-5935. doi: 10.1002/2017GL073549

Hughes, W. J., & Southwood, D. J. (1976, July). The screening of micropulsation signals by the atmosphere and ionosphere. *J. Geophys. Res.*, *81*(19), 3234. doi: 10.1029/JA081i019p03234

Jakowski, N., Mayer, C., Hoque, M., & Wilken, V. (2011). Total electron content models and their use in ionosphere monitoring. *Radio Science*, *46*(06), 1–11.

Jakowski, N., Sardon, E., Engler, E., Jungstand, A., & Klähn, D. (1996, December). Relationships between GPS-signal propagation errors and EISCAT observations. *Annales Geophysicae*, *14*(12), 1429-1436. doi: 10.1007/s00585-996-1429-0

Jakowski, N., Wilken, V., & Mayer, C. (2007, August). Space weather monitoring by GPS measurements on board CHAMP. *Space Weather*, *5*(8), 08006. doi: 10.1029/2006SW000271

Jaynes, A. N., Lessard, M. R., Takahashi, K., Ali, A. F., Malaspina, D. M., Michell, R. G., . . . Wygant, J. R. (2015, October). Correlated Pc4-5 ULF waves, whistler-mode chorus, and pulsating aurora observed by the Van Allen Probes and ground-based systems. *J. Geophys. Res.*, *120*, 8749-8761. doi: 10.1002/2015JA021380

Jin, Y., Moen, J. I., & Miloch, W. J. (2015). On the collocation of the cusp aurora and the GPS phase scintillation: A statistical study. *Journal of Geophysical Research: Space Physics*, *120*(10), 9176–9191.

Jin, Y., Xiong, C., Clausen, L., Spicher, A., Kotova, D., Brask, S., . . . Miloch, W. (2020, July). Ionospheric Plasma Irregularities Based on In Situ Measurements From the Swarm Satellites. *Journal of Geophysical Research (Space Physics)*, *125*(7), e28103. doi: 10.1029/2020JA028103

Kaeppler, S. R., Marshall, R., Sanchez, E. R., Juarez Madera, D. H., Troyer, R., & Jaynes, A. N. (2022, December). pyGPI5: A python D- and E-region chemistry and ionization model. *Frontiers in Astronomy and Space Sciences*, *9*,

338. doi: 10.3389/fspas.2022.1028042

Kasahara, S., Miyoshi, Y., Yokota, S., Kasahara, Y., Matsuda, S., Kumamoto, A., ... Shinohara, I. (2018). Pulsating aurora from electron scattering by chorus waves. *Nature*, *554*, 337-340. doi: 10.1038/nature25505

Katoh, Y., Rosendahl, P. S., Ogawa, Y., Hiraki, Y., & Tadokoro, H. (2023, December). Effect of the mirror force on the collision rate due to energetic electron precipitation: Monte Carlo simulations. *Earth, Planets and Space*, *75*(1), 117. doi: 10.1186/s40623-023-01871-y

Kelley, M. C. (2009). *The Earth's ionosphere: Plasma physics and electrodynamics.* Academic press.

Kennel, C. F., & Engelmann, F. (1966, November). Velocity Space Diffusion from Weak Plasma Turbulence in a Magnetic Field. *Physics of Fluids*, *9*, 2377-2388. doi: 10.1063/1.1761629

Kennel, C. F., & Petschek, H. E. (1966, January). Limit on Stably Trapped Particle Fluxes. *J. Geophys. Res.*, *71*, 1-28.

Keskinen, M. J., & Ossakow, S. L. (1983, January). Nonlinear evolution of convecting plasma enhancements in the auroral ionosphere, 2. Small scale irregularities. *J. Geophys. Res.*, *88*(A1), 474-482. doi: 10.1029/JA088iA01p00474

Kintner, P. M., Ledvina, B. M., & de Paula, E. R. (2007, September). GPS and ionospheric scintillations. *Space Weather*, *5*(9), 09003. doi: 10.1029/2006SW000260

Komjathy, A. (1997). *Global ionospheric total electron content mapping using the global positioning system* (Unpublished doctoral dissertation). University of New Brunswick, Canada.

Komjathy, A., Sparks, L., Wilson, B. D., & Mannucci, A. J. (2005, December). Automated daily processing of more than 1000 ground-based GPS receivers for studying intense ionospheric storms. *Radio Science*, *40*(6), RS6006. doi: 10.1029/2005RS003279

Komjathy, A., Yang, Y.-M., Meng, X., Verkhoglyadova, O., Mannucci, A. J., & Langley, R. B. (2016, July). Review and perspectives: Understanding natural-hazards-generated ionospheric perturbations using GPS measurements and coupled modeling. *Radio Science*, *51*(7), 951-961. doi: 10.1002/2015RS005910

Le Contel, O., Roux, A., Robert, P., Coillot, C., Bouabdellah, A., de La Porte, B., ... Larson, D. (2008, December). First Results of the THEMIS Search Coil Magnetometers. *Space Sci. Rev.*, *141*, 509-534. doi: 10.1007/s11214-008-9371-y

Lessard, M. R., & Knudsen, D. J. (2001, October). Ionospheric reflection of small-scale Alfvén waves. *Geophys. Res. Lett.*, *28*(18), 3573-3576. doi: 10.1029/2000GL012529

Li, L., Omura, Y., Zhou, X.-Z., Zong, Q.-G., Rankin, R., Yue, C., & Fu, S.-Y. (2022, May). Nonlinear Wave Growth Analysis of Chorus Emissions Modulated by ULF Waves. *Geophys. Res. Lett.*, *49*(10), e97978. doi: 10.1029/2022GL097978

Li, L., Omura, Y., Zhou, X.-Z., Zong, Q.-G., Rankin, R., Yue, C., ... Ren, J. (2023, February). Chorus Wave Generation Modulated by Field Line Resonance and Mirror-Mode ULF Waves. *Journal of Geophysical Research (Space Physics)*, *128*(2), e2022JA031127. doi: 10.1029/2022JA031127

Li, W., Bortnik, J., Thorne, R. M., & Angelopoulos, V. (2011, December). Global distribution of wave amplitudes and wave normal angles of chorus waves using THEMIS wave observations. *J. Geophys. Res.*, *116*, 12205. doi: 10.1029/2011JA017035

Li, W., Bortnik, J., Thorne, R. M., Nishimura, Y., Angelopoulos, V., & Chen, L. (2011, June). Modulation of whistler mode chorus waves: 2. Role of density variations. *J. Geophys. Res.*, *116*, A06206. doi: 10.1029/2010JA016313

Li, W., Thorne, R. M., Bortnik, J., Nishimura, Y., & Angelopoulos, V. (2011, June). Modulation of whistler mode chorus waves: 1. Role of compressional Pc4-5 pulsations. *J. Geophys. Res.*, *116*, A06205. doi: 10.1029/2010JA016312

Liang, J., Shen, Y., Knudsen, D., Spanswick, E., Burchill, J., & Donovan, E. (2019, June). e-POP and Red Line Optical Observations of Alfvénic Auroras. *Journal of Geophysical Research (Space Physics)*, *124*(6), 4672-4696. doi: 10.1029/2019JA026679

Lyons, L. R. (1974, December). Pitch angle and energy diffusion coefficients from resonant interactions with ion-cyclotron and whistler waves. *Journal of Plasma Physics*, *12*, 417-432. doi: 10.1017/S002237780002537X

Lysak, R. L. (1991, February). Feedback instability of the ionospheric resonant cav-

ity. *J. Geophys. Res.*, *96*(A2), 1553-1568. doi: 10.1029/90JA02154

Ma, Q., Connor, H. K., Zhang, X. J., Li, W., Shen, X. C., Gillespie, D., ... Spence, H. E. (2020, August). Global Survey of Plasma Sheet Electron Precipitation due to Whistler Mode Chorus Waves in Earth's Magnetosphere. *Geophys. Res. Lett.*, *47*(15), e88798. doi: 10.1029/2020GL088798

Makarevich, R. A., Crowley, G., Azeem, I., Ngwira, C., & Forsythe, V. V. (2021, May). Auroral E Region as a Source Region for Ionospheric Scintillation. *Journal of Geophysical Research (Space Physics)*, *126*(5), e29212. doi: 10.1029/2021JA029212

Mannucci, A. J., Wilson, B. D., Yuan, D. N., Ho, C. H., Lindqwister, U. J., & Runge, T. F. (1998, May). A global mapping technique for GPS-derived ionospheric total electron content measurements. *Radio Science*, *33*(3), 565-582. doi: 10.1029/97RS02707

McCaffrey, A. M., & Jayachandran, P. T. (2017, June). Observation of subsecond variations in auroral region total electron content using 100 Hz sampling of GPS observables. *Journal of Geophysical Research (Space Physics)*, *122*(6), 6892-6900. doi: 10.1002/2017JA024255

McCaffrey, A. M., & Jayachandran, P. T. (2019, February). Determination of the Refractive Contribution to GPS Phase "Scintillation". *Journal of Geophysical Research (Space Physics)*, *124*(2), 1454-1469. doi: 10.1029/2018JA025759

McFadden, J. P., Carlson, C. W., Larson, D., Ludlam, M., Abiad, R., Elliott, B., ... Angelopoulos, V. (2008, December). The THEMIS ESA Plasma Instrument and In-flight Calibration. *Space Sci. Rev.*, *141*, 277-302. doi: 10.1007/s11214-008-9440-2

McPherron, R. L. (1972, September). Substorm related changes in the geomagnetic tail: The growth phase. *Planetary Space Science*, *20*, 1521-1539. doi: 10.1016/0032-0633(72)90054-2

Meng, X., Mannucci, A. J., Verkhoglyadova, O. P., & Tsurutani, B. T. (2016, April). On forecasting ionospheric total electron content responses to high-speed solar wind streams. *Journal of Space Weather and Space Climate*, *6*, A19. doi: 10.1051/swsc/2016014

Meng, X., Mannucci, A. J., Verkhoglyadova, O. P., Tsurutani, B. T., Ridley, A. J., & Shim, J.-S. (2020, February). Thermosphere-Ionosphere Modeling With

Forecastable Inputs: Case Study of the June 2012 High-Speed Stream Geomagnetic Storm. *Space Weather*, *18*(2), e02352. doi: 10.1029/2019SW002352

Meng, X., Ozturk, D. S., Verkhoglyadova, O. P., Varney, R. H., Reimer, A. S., Semeter, J. L., ... Zhan, W. (2022, December). Energy Deposition by Mesoscale High-Latitude Electric Fields Into the Thermosphere During the 26 October 2019 Geomagnetic Storm. *Journal of Geophysical Research (Space Physics)*, *127*(12), e2022JA030716. doi: 10.1029/2022JA030716

Meredith, N. P., Bortnik, J., Horne, R. B., Li, W., & Shen, X.-C. (2021). Statistical investigation of the frequency dependence of the chorus source mechanism of plasmaspheric hiss. *Geophysical Research Letters*, *48*(6), e2021GL092725. Retrieved from `https://agupubs.onlinelibrary.wiley.com/doi/abs/10.1029/2021GL092725` (e2021GL092725 2021GL092725) doi: https://doi.org/10.1029/2021GL092725

Miyoshi, Y., Katoh, Y., Nishiyama, T., Sakanoi, T., Asamura, K., & Hirahara, M. (2010, October). Time of flight analysis of pulsating aurora electrons, considering wave-particle interactions with propagating whistler mode waves. *J. Geophys. Res.*, *115*, A10312. doi: 10.1029/2009JA015127

Moen, J., Oksavik, K., Alfonsi, L., Daabakk, Y., Romano, V., & Spogli, L. (2013, January). Space weather challenges of the polar cap ionosphere. *Journal of Space Weather and Space Climate*, *3*, A02. doi: 10.1051/swsc/2013025

Newell, P. T., Sotirelis, T., & Wing, S. (2009, September). Diffuse, monoenergetic, and broadband aurora: The global precipitation budget. *J. Geophys. Res.*, *114*, A09207. doi: 10.1029/2009JA014326

Nishimura, Y., Bortnik, J., Li, W., Thorne, R. M., Lyons, L. R., Angelopoulos, V., ... Auster, U. (2010, October). Identifying the Driver of Pulsating Aurora. *Science*, *330*, 81-84. doi: 10.1126/science.1193186

Nishimura, Y., Bortnik, J., Li, W., Thorne, R. M., Ni, B., Lyons, L. R., ... Auster, U. (2013, Feb). Structures of dayside whistler-mode waves deduced from conjugate diffuse aurora. *Journal of Geophysical Research (Space Physics)*, *118*(2), 664-673. doi: 10.1029/2012JA018242

Nishimura, Y., Kelly, T., Jayachandran, P. T., Mrak, S., Semeter, J. L., Donovan, E. F., ... Nishitani, N. (2023, August). Nightside High-Latitude Phase and Amplitude Scintillation During a Substorm Using 1-Second Scin-

tillation Indices. *Journal of Geophysical Research (Space Physics)*, *128*(8), e2023JA031402. doi: 10.1029/2023JA031402

Okuzawa, T., & Davies, K. (1981, March). Pulsations in total columnar electron content. *J. Geophys. Res.*, *86*(A3), 1355-1364. doi: 10.1029/JA086iA03p01355

Pi, X., Mannucci, A. J., Lindqwister, U. J., & Ho, C. M. (1997, September). Monitoring of global ionospheric irregularities using the Worldwide GPS Network. *Geophys. Res. Lett.*, *24*(18), 2283-2286. doi: 10.1029/97GL02273

Picone, J. M., Hedin, A. E., Drob, D. P., & Aikin, A. C. (2002, December). NRLMSISE-00 empirical model of the atmosphere: Statistical comparisons and scientific issues. *Journal of Geophysical Research (Space Physics)*, *107*(A12), 1468. doi: 10.1029/2002JA009430

Pilipenko, V., Belakhovsky, V., Murr, D., Fedorov, E., & Engebretson, M. (2014, June). Modulation of total electron content by ULF Pc5 waves. *Journal of Geophysical Research (Space Physics)*, *119*(6), 4358-4369. doi: 10.1002/2013JA019594

Prikryl, P., Jayachandran, P. T., Chadwick, R., & Kelly, T. D. (2015, May). Climatology of GPS phase scintillation at northern high latitudes for the period from 2008 to 2013. *Annales Geophysicae*, *33*(5), 531-545. doi: 10.5194/angeo-33-531-2015

Ridley, A. J., Deng, Y., & Tóth, G. (2006, May). The global ionosphere thermosphere model. *Journal of Atmospheric and Solar-Terrestrial Physics*, *68*(8), 839-864. doi: 10.1016/j.jastp.2006.01.008

Sandhu, J. K., Rae, I. J., Staples, F. A., Hartley, D. P., Walach, M. T., Elsden, T., & Murphy, K. R. (2021, July). The Roles of the Magnetopause and Plasmapause in Storm-Time ULF Wave Power Enhancements. *Journal of Geophysical Research (Space Physics)*, *126*(7), e29337. doi: 10.1029/2021JA029337

SantolíK, O., Gurnett, D. A., Pickett, J. S., Parrot, M., & Cornilleau-Wehrlin, N. (2003, July). Spatio-temporal structure of storm-time chorus. *J. Geophys. Res.*, *108*, 1278. doi: 10.1029/2002JA009791

Semeter, J., Mrak, S., Hirsch, M., Swoboda, J., Akbari, H., Starr, G., … Pankratius, V. (2017, October). GPS Signal Corruption by the Discrete Aurora: Precise Measurements From the Mahali Experiment. *Geophys. Res.*

Lett., *44* (19), 9539-9546. doi: 10.1002/2017GL073570

Shen, Y., Artemyev, A., Zhang, X.-J., Vasko, I. Y., Runov, A., Angelopoulos, V., & Knudsen, D. (2020, August). Potential Evidence of Low-Energy Electron Scattering and Ionospheric Precipitation by Time Domain Structures. *Geophys. Res. Lett.*, *47* (16), e89138. doi: 10.1029/2020GL089138

Sheng, C., Deng, Y., Zhang, S.-R., Nishimura, Y., & Lyons, L. R. (2020, February). Relative Contributions of Ion Convection and Particle Precipitation to Exciting Large-Scale Traveling Atmospheric and Ionospheric Disturbances. *Journal of Geophysical Research (Space Physics)*, *125* (2), e27342. doi: 10.1029/2019JA027342

Shi, R., Li, W., Ma, Q., Green, A., Kletzing, C. A., Kurth, W. S., ... Reeves, G. D. (2019, February). Properties of Whistler Mode Waves in Earth's Plasmasphere and Plumes. *Journal of Geophysical Research (Space Physics)*, *124* (2), 1035-1051. doi: 10.1029/2018JA026041

Shi, X., Baker, J. B. H., Ruohoniemi, J. M., Hartinger, M. D., Murphy, K. R., Rodriguez, J. V., ... Angelopoulos, V. (2018, October). Long-Lasting Poloidal ULF Waves Observed by Multiple Satellites and High-Latitude SuperDARN Radars. *Journal of Geophysical Research (Space Physics)*, *123* (10), 8422-8438. doi: 10.1029/2018JA026003

Shi, X., Zhang, X.-J., Artemyev, A., Angelopoulos, V., Hartinger, M. D., Tsai, E., & Wilkins, C. (2022, December). On the Role of ULF Waves in the Spatial and Temporal Periodicity of Energetic Electron Precipitation. *Journal of Geophysical Research (Space Physics)*, *127* (12), e2022JA030932. doi: 10.1029/2022JA030932

Skone, S. (2009, February). Using GPS TEC measurements to detect geomagnetic Pc 3 pulsations. *Radio Science*, *44* (19), RS0A27. doi: 10.1029/2008RS004106

Spicher, A., Clausen, L. B. N., Miloch, W. J., Lofstad, V., Jin, Y., & Moen, J. I. (2017, March). Interhemispheric study of polar cap patch occurrence based on Swarm in situ data. *Journal of Geophysical Research (Space Physics)*, *122* (3), 3837-3851. doi: 10.1002/2016JA023750

Spogli, L., Alfonsi, L., de Franceschi, G., Romano, V., Aquino, M. H. O., & Dodson, A. (2009, September). Climatology of GPS ionospheric scintillations over high and mid-latitude European regions. *Annales Geophysicae*, *27* (9), 3429-3437.

doi: 10.5194/angeo-27-3429-2009

Streltsov, A. V., & Lotko, W. (2008, May). Coupling between density structures, electromagnetic waves and ionospheric feedback in the auroral zone. *Journal of Geophysical Research (Space Physics)*, *113*(A5), A05212. doi: 10.1029/2007JA012594

Takahashi, K., & Anderson, B. J. (1992, July). Distribution of ULF energy (f<=80 mHz) in the inner magnetosphere: A statistical analysis of AMPTE CCE magnetic field data. *J. Geophys. Res.*, *97*(A7), 10751-10773. doi: 10.1029/92JA00328

Tsunoda, R. T. (1988, November). High-latitude F-region irregularities: A review and synthesis. *Reviews of Geophysics*, *26*(4), 719-760. doi: 10.1029/RG026i004p00719

Tsurutani, B. T., & Smith, E. J. (1974, January). Postmidnight chorus: A substorm phenomenon. *J. Geophys. Res.*, *79*, 118-127. doi: 10.1029/JA079i001p00118

Tsyganenko, N. A. (1995, April). Modeling the Earth's magnetospheric magnetic field confined within a realistic magnetopause. *J. Geophys. Res.*, *100*, 5599-5612. doi: 10.1029/94JA03193

Tyler, E., Breneman, A., Cattell, C., Wygant, J., Thaller, S., & Malaspina, D. (2019, Mar). Statistical Occurrence and Distribution of High-Amplitude Whistler Mode Waves in the Outer Radiation Belt. *Geophys. Res. Lett.*, *46*(5), 2328-2336. doi: 10.1029/2019GL082292

Verkhoglyadova, O., Meng, X., Mannucci, A. J., Shim, J. S., & McGranaghan, R. (2020, September). Evaluation of Total Electron Content Prediction Using Three Ionosphere-Thermosphere Models. *Space Weather*, *18*(9), e2020SW002452. doi: 10.1029/2020SW002452

Wang, B., Nishimura, Y., Hartinger, M., Sivadas, N., Lyons, L. L., Varney, R. H., & Angelopoulos, V. (2020, August). Ionospheric Modulation by Storm Time Pc5 ULF Pulsations and the Structure Detected by PFISR-THEMIS Conjunction. *Geophys. Res. Lett.*, *47*(16), e89060. doi: 10.1029/2020GL089060

Watson, C., Jayachandran, P. T., & MacDougall, J. W. (2016, May). Characteristics of GPS TEC variations in the polar cap ionosphere. *Journal of Geophysical Research (Space Physics)*, *121*(5), 4748-4768. doi: 10.1002/2015JA022275

Watson, C., Jayachandran, P. T., Singer, H. J., Redmon, R. J., & Danskin, D.

(2015, September). Large-amplitude GPS TEC variations associated with Pc5-6 magnetic field variations observed on the ground and at geosynchronous orbit. *Journal of Geophysical Research (Space Physics)*, *120*(9), 7798-7821. doi: 10.1002/2015JA021517

Watson, C., Jayachandran, P. T., Singer, H. J., Redmon, R. J., & Danskin, D. (2016, February). GPS TEC response to Pc4 "giant pulsations". *Journal of Geophysical Research (Space Physics)*, *121*(2), 1722-1735. doi: 10.1002/2015JA022253

Watson, C., Jayachandran, P. T., Spanswick, E., Donovan, E. F., & Danskin, D. W. (2011, October). GPS TEC technique for observation of the evolution of substorm particle precipitation. *Journal of Geophysical Research (Space Physics)*, *116*(A10), A00I90. doi: 10.1029/2010JA015732

Watt, C. E. J., Degeling, A. W., Rankin, R., Murphy, K. R., Rae, I. J., & Singer, H. J. (2011, October). Ultralow-frequency modulation of whistler-mode wave growth. *Journal of Geophysical Research (Space Physics)*, *116*(A10), A10209. doi: 10.1029/2011JA016730

Xia, Z., Chen, L., Dai, L., Claudepierre, S. G., Chan, A. A., Soto-Chavez, A. R., & Reeves, G. D. (2016, September). Modulation of chorus intensity by ULF waves deep in the inner magnetosphere. *Geophys. Res. Lett.*, *43*, 9444-9452. doi: 10.1002/2016GL070280

Xia, Z., Chen, L., & Li, W. (2020, November). Statistical Study of Chorus Modulations by Background Magnetic Field and Plasma Density. *Geophys. Res. Lett.*, *47*(22), e89344. doi: 10.1029/2020GL089344

Xiong, C., Park, J., Lühr, H., Stolle, C., & Ma, S. Y. (2010, September). Comparing plasma bubble occurrence rates at CHAMP and GRACE altitudes during high and low solar activity. *Annales Geophysicae*, *28*(9), 1647-1658. doi: 10.5194/angeo-28-1647-2010

Yeh, K. C., & Liu, C. H. (1982, April). Radio wave scintillations in the ionosphere. *IEEE Proceedings*, *70*, 324-360.

Yeoman, T. K., James, M., Mager, P. N., & Klimushkin, D. Y. (2012, June). SuperDARN observations of high-m ULF waves with curved phase fronts and their interpretation in terms of transverse resonator theory. *Journal of Geophysical Research (Space Physics)*, *117*(A6), A06231. doi: 10.1029/2012JA017668

Zettergren, M. D., & Snively, J. B. (2015, September). Ionospheric response to infrasonic-acoustic waves generated by natural hazard events. *Journal of Geophysical Research (Space Physics)*, *120*(9), 8002-8024. doi: 10.1002/2015JA021116

Zhai, C., Shi, X., Wang, W., Hartinger, M. D., Yao, Y., Peng, W., ... Baker, J. B. H. (2021, July). Characterization of High-m ULF Wave Signatures in GPS TEC Data. *Geophys. Res. Lett.*, *48*(14), e94282. doi: 10.1029/2021GL094282

Zhang, X. J., Angelopoulos, V., Artemyev, A. V., Hartinger, M. D., & Bortnik, J. (2020, October). Modulation of Whistler Waves by Ultra-Low-Frequency Perturbations: The Importance of Magnetopause Location. *Journal of Geophysical Research (Space Physics)*, *125*(10), e28334. doi: 10.1029/2020JA028334

Zhang, X.-J., Chen, L., Artemyev, A. V., Angelopoulos, V., & Liu, X. (2019, November). Periodic Excitation of Chorus and ECH Waves Modulated by Ultralow Frequency Compressions. *Journal of Geophysical Research (Space Physics)*, *124*(11), 8535-8550. doi: 10.1029/2019JA027201

Zong, Q., Rankin, R., & Zhou, X. (2017, Dec). The interaction of ultra-low-frequency pc3-5 waves with charged particles in Earth's magnetosphere. *Reviews of Modern Plasma Physics*, *1*(1), 10. doi: 10.1007/s41614-017-0011-4
