# Supplementary material for: Magnetospheric Control of Ionospheric TEC Perturbations via Whistler‐Mode and ULF Waves
Source: AGU Adv. 2024 Nov 25;5(6):e2024AV001302. doi: 10.1029/2024AV001302 (PMC11600791; doi:10.1029/2024AV001302)
Supplement: Supplementary file 6 — First Revision of Manuscript [file AGA2-5-0-s002.pdf]

Magnetospheric control of ionospheric TEC perturbations via whistler-mode and ULF waves

Yangyang Shen[1], Olga P. Verkhoglyadova[2], Anton Artemyev[1], Michael D. Hartinger[3,1], Vassilis Angelopoulos[1], Xueling Shi[4], Ying Zou[5]

[1]Department of Earth, Planetary, and Space Sciences, University of California, Los Angeles, CA, USA
[2]Jet Propulsion Laboratory, California Institute of Technology, Pasadena, CA, USA
[3]Space Science Institute, Center for Space Plasma Physics, Boulder, CO, USA
[4]Department of Electrical and Computer Engineering, Virginia Tech, Blacksburg, VA, USA
[5]Johns Hopkins University Applied Physics Laboratory, Laurel, MD, USA

Key Points:

- Space-ground conjugate observations point to magnetospheric whistler-mode waves as the driver of ionospheric TEC perturbations (dTEC)
- The amplitude spectra of dTEC and whistlers are consistent and the cross-correlation between modeled and observed dTEC reaches 0.8
- Whistler-mode wave amplitudes and dTEC are modulated by ULF waves, which exhibit concurrent compressional and poloidal mode variations

Corresponding author: Yangyang Shen, `yshen@epss.ucla.edu`

Abstract

The weakly ionized plasma in the Earth's ionosphere is controlled by a complex interplay between solar and magnetospheric inputs from above, atmospheric processes from below, and plasma electrodynamics from within. This interaction results in ionosphere structuring and variability that pose major challenges for accurate ionosphere prediction for global navigation satellite system (GNSS) related applications and space weather research. The ionospheric structuring and variability are often probed using the total electron content (TEC) and its relative perturbations (dTEC). Among dTEC variations observed at high latitudes, a unique modulation pattern has been linked to magnetospheric ultra-low-frequency (ULF) waves, yet its underlying mechanisms remain unclear. Here using magnetically-conjugate observations from the THEMIS spacecraft and a ground-based GPS receiver at Fairbanks, Alaska, we provide direct evidence that these dTEC modulations are driven by magnetospheric electron precipitation induced by ULF-modulated whistler-mode waves. We observed peak-to-peak dTEC amplitudes reaching ∼0.5 TECU (1 TECU is equal to 10^6 electrons/m^2) with modulations spanning scales of ∼5–80 km. The cross-correlation between our modeled and observed dTEC reached ∼0.8 during the conjugacy period but decreased outside of it. The spectra of whistler-mode waves and dTEC also matched closely at ULF frequencies during the conjugacy period but diverged outside of it. Our findings elucidate the high-latitude dTEC generation from magnetospheric wave-induced precipitation, addressing a significant gap in current physics-based TEC modeling. Theses results thus improve ionospheric TEC prediction and enhance our understanding of magnetosphere-ionosphere coupling via ULF waves.

Plain Language Summary

Radio signals are refracted or diffracted as they traverse the ionosphere filled with free electrons. The ionosphere total electron content (TEC), which is the total number of electrons along the raypath from the satellite to a receiver, helps to correct refractive errors in the signal, while its relative perturbations (dTEC) can be used to probe diffractive fluctuations known as ionosphere scintillation. Refractive error degrades global navigation satellite system (GNSS) positioning service accuracy while scintillation leads to signal reception failures and disrupts navigation and communication. Thus, an accurate understanding and modeling of TEC and dTEC is vital for space weather monitoring and GNSS-related applications. This study analyzes fortuitous observations of ionospheric

dTEC from a ground-based GPS receiver and magnetospheric whistler-mode waves (a distinct type of very-low-frequency electromagnetic waves) from the THEMIS spacecraft, which were well-aligned both in time and space. We find a good cross-correlation (∼0.8) between observed and modeled dTEC, driven by whistler-induced magnetospheric electron precipitation. These results point to whistler-mode waves as the driver of the observed dTEC. Both dTEC and whistler-mode wave amplitudes were modulated by ultra-low-frequency (ULF) waves. These findings enhance physics-based ionospheric TEC prediction and our understanding of magnetosphere-ionosphere coupling.

1 Introduction

The Earth's ionosphere contains weakly ionized plasma in the atmosphere between approximately 80 km and 1000 km altitude. The state of ionospheric plasma is controlled by a complex interplay between solar and magnetospheric inputs from above, neutral atmospheric processes from below, and plasma electrodynamics from within. The resulting structuring and variability of ionospheric plasma have a major, adverse impact on the global navigation satellite system (GNSS) radio signals as they propagate through the ionosphere and experience varying degrees of refraction and diffraction (Morton et al., 2020). Refraction causes signal group delay and phase advance, leading to dominant errors in GNSS position, velocity, and time solutions, while diffraction causes stochastic intensity and phase fluctuations at the receiver, commonly known as ionospheric scintillation (Yeh & Liu, 1982; Rino, 2011). Scintillation leads to increased GNSS receiver measurement noise and errors and, in extreme cases, phase-tracking loss of lock or signal reception failures (Kintner et al., 2007). Thus, these ionospheric effects pose real threats to the reliability, continuity, and accuracy of GNSS operations and applications (Morton et al., 2020; Coster & Yizengaw, 2021). Understanding the causes for ionospheric structuring and variability is critical for forecasting their impacts on GNSS applications—a long-standing challenge for space weather research (Hey et al., 1946; Jakowski et al., 2011; Morton et al., 2020). The importance of this ionosphere forecasting has recently gained increased attention as the solar maximum unfolds and concerns over space weather events such as geomagnetic storms loom large (e.g., Kintner et al., 2007; Pulkkinen et al., 2017; Hapgood et al., 2022).

Ionospheric refraction is typically quantified by the total electron content (TEC), which is the total number of electrons within a unit cross section along the raypath ex-

tending from the receiver to the satellite. For dual-frequency GNSS or Global Positioning System (GPS) receivers, the TEC is estimated from differential group delays and carrier-phase advances (Mannucci et al., 1998; Ciraolo et al., 2007; McCaffrey & Jayachandran, 2017). Global TEC maps, constructed from networks of GNSS receivers on the ground and in orbit, can be used not only to correct ionospheric effects in GNSS-related applications but also to monitor large- and meso-scale traveling ionospheric disturbances (TIDs), typically exceeding 100 km in horizontal wavelength (Hunsucker, 1982; Themens et al., 2022; S.-R. Zhang et al., 2022). TIDs may result from internal ionospheric dynamics or from atmospheric effects from below linked to natural hazards, such as tsunamis, earthquakes, explosions, and volcanic eruptions (Komjathy et al., 2016; Astafyeva, 2019). High-resolution TEC from individual receivers and its relative perturbations (dTEC) and rate of changes (ROTI) are often used for detecting small-scale ionospheric irregularities and scintillation events (Pi et al., 1997; Cherniak et al., 2014; McCaffrey & Jayachandran, 2019; Makarevich et al., 2021; Nishimura et al., 2023).

One of the main challenges in ionospheric TEC modeling and space weather prediction is the complex structuring and variability of ionosphere plasma. Rapid (<a few minutes) and small-scale (<∼100 km) TEC perturbations are observed at both low and high latitudes but generated by distinct mechanisms and drivers (Pi et al., 1997; Basu et al., 2002; Kintner et al., 2007; Spogli et al., 2009; Moen et al., 2013; Pilipenko et al., 2014; Jin et al., 2015; Prikryl et al., 2015; Watson, Jayachandran, Singer, et al., 2016; Fæhn Follestad et al., 2020). Near equatorial latitudes, these small-scale TEC perturbations result from plasma bubbles or density depletions formed around post-sunset, primarily driven by the Rayleigh-Taylor instability associated with lower atmosphere-ionosphere coupling processes (C.-S. Huang & Kelley, 1996; Kelley, 2009; Xiong et al., 2010; Aa et al., 2020; Jin et al., 2020). At high latitudes, TEC perturbations are associated with plasma irregularities in the auroral, cusp, and polar cap regions, spanning a few meters to hundreds of kilometers in spatial scale (e.g., Basu et al., 1990; Moen et al., 2013; Spicher et al., 2017). These irregularities are primarily driven by solar-magnetosphere-ionosphere coupling, which involves a complex interplay and synergy among solar extreme-ultraviolet (EUV) radiation, plasma $\mathbf{E} \times \mathbf{B}$ drifts, charged-particle precipitation into the atmosphere, magnetic field-aligned currents, and various ionospheric plasma instabilities (Kelley, 2009; Moen et al., 2013; Spicher et al., 2015; Fæhn Follestad et al., 2020).

Among dTEC variations observed near the auroral latitudes, a unique modulation pattern has been linked to magnetospheric ultra-low-frequency (ULF) waves (Davies & Hartmann, 1976; Okuzawa & Davies, 1981; Skone, 2009; Pilipenko et al., 2014; Watson et al., 2015; Watson, Jayachandran, Singer, et al., 2016; Zhai et al., 2021). These ULF waves feature broadband or quasi-monochromatic geomagnetic pulsations with periods from about 0.2 to 600 s (Jacobs et al., 1964) and are considered to be crucial for energy and plasma transport throughout the solar-magnetosphere-ionosphere-thermosphere system (e.g., Southwood & Kivelson, 1981; M. K. Hudson et al., 2000, 2008; Hartinger et al., 2015, 2022; Zong et al., 2017). Skone (2009) noted that average power of ground-based ULF waves and TEC perturbations exhibited similar temporal variations in the Pc3 band ($\sim$22–100 mHz). Pilipenko et al. (2014) observed a high coherence ($\sim$0.9) between TEC perturbations and global Pc5 pulsations in a few mHz during a geomagnetic storm. Watson, Jayachandran, Singer, et al. (2016) also reported a high coherence and common power between TEC perturbations and ULF radial magnetic field variations in the Pc4 band (6.7–22 mHz). Fully understanding ULF-induced ionospheric TEC perturbations not only enhances the ionosphere forecasting during space weather events but also elucidates the critical pathways of geospace energy coupling and dissipation via ULF waves.

To date, despite numerous proposals for direct TEC modulation mechanisms by ULF waves (Pilipenko et al., 2014), no mechanism has yet been conclusively established. Recently, Wang et al. (2020) have reported a storm-time event where duskside ionospheric density was modulated by ULF waves in the Pc5 range. Density inversion from radar data suggests that Pc5 pulsations modulated precipitating electrons over an energy range of $\sim$1–500 keV and an altitude range of $\sim$80–200 km. Higher-energy precipitating electrons deposit their energy and induce impact ionization at lower altitudes, whereas lower-energy electrons do so at higher altitudes. The authors postulated that the precipitation and density perturbations are likely due to electron pitch-angle scattered into the loss cone by ULF-modulated very-low-frequency (VLF) whistler-mode waves.

This postulation is supported by extensive observations and models that demonstrate that ULF waves often coexist with and modulate whistler-mode waves (Coroniti & Kennel, 1970; W. Li, Thorne, et al., 2011; W. Li, Bortnik, Thorne, Nishimura, et al., 2011; Watt et al., 2011; Jaynes et al., 2015; Xia et al., 2016, 2020; X.-J. Zhang et al., 2019; X. J. Zhang et al., 2020; X. Shi et al., 2022; L. Li et al., 2022, 2023). The modulation of the whistler-mode wave growth is potentially attributed to compression-induced am-

bient thermal or resonant hot electron density variations (W. Li, Bortnik, Thorne, Nishimura, et al., 2011; Xia et al., 2016, 2020; X.-J. Zhang et al., 2019; X. J. Zhang et al., 2020), resonant electron anisotropy variations (W. Li, Thorne, et al., 2011; Watt et al., 2011), and nonlinear resonant effects from periodic magnetic field configuration variations (L. Li et al., 2022, 2023). The periodic excitation of whistler-mode waves at ULF wave frequencies leads to periodic electron precipitation, which drives pulsating auroras (e.g., Miyoshi et al., 2010; Nishimura et al., 2010; Jaynes et al., 2015) and potentially explains many previously reported TEC modulations at ULF frequencies (Pilipenko et al., 2014; Watson, Jayachandran, Singer, et al., 2016; Zhai et al., 2021).

However, it is challenging to establish a direct link between magnetospheric drivers and ionospheric TEC perturbations during ULF modulation events due to several complicating factors: (1) the path-integrated nature of TEC perturbations, which strongly depend on the satellite-to-receiver raypath elevation (e.g., Jakowski et al., 1996; Komjathy, 1997), (2) inherent phase shifts due to coexisting propagation and modulation effects (Watson et al., 2015), particularly when conjugate observations are misaligned or not synchronized, and (3) the dynamic and turbulent nature of the auroral ionosphere (Kelley, 2009). Direct evidence linking TEC perturbations to magnetospheric drivers is yet to be identified.

In this study, fortuitously conjugate observations from the THEMIS spacecraft and the GPS receiver at Fairbanks, Alaska (FAIR) allow us to identify the driver of GPS TEC perturbations as magnetospheric electron precipitation induced by ULF-modulated whistler-mode waves. Figure 1 illustrates the physical picture emerging from these magnetically-conjugate magnetospheric and ionospheric observations of ULF waves, modulated whistler-mode waves, electron precipitation, and TEC perturbations.

In what follows, Section 2 describes datasets and models employed to estimate whistler-driven precipitation and resulting TEC perturbations. Section 3 presents a detailed analysis and cross-correlation between observed and modeled TEC perturbations. Section 4 discusses the geophysical implications and applications of our results, which are followed by the main conclusions.

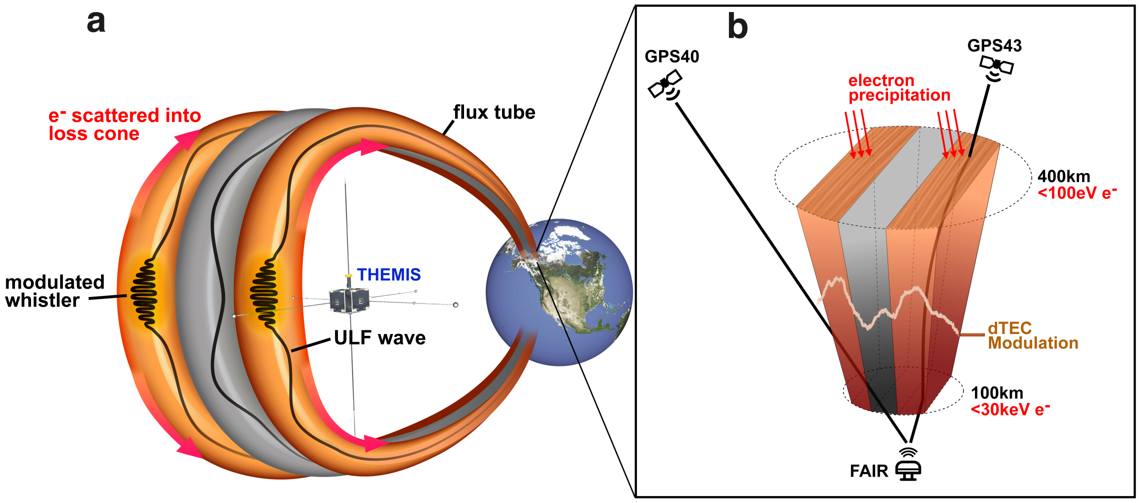

Figure 1. Schematic diagram showing coordinated observations from THEMIS and FAIR of (a) modulation of very-low-frequency (VLF) whistler-mode waves near the magnetic equator by ultra-low-frequency (ULF) waves, electron pitch-angle scattering into the loss cone, and precipitation into the ionosphere (red arrows) induced by modulated whistler-mode waves; and (b) the modulated electron precipitation with energies of ∼0.1–30 keV deposits their energies at altitudes between ∼100–400 km and induces modulated impact ionization and TEC perturbations (dTEC) having amplitudes as large as ∼0.5 TECU and spanning scales of ∼5–80 km. This dTEC modulation was captured by the signal from GPS43, which has a high elevation, but was overlooked by the signal from GPS40, which has a relatively lower elevation.

2 Data and Methodology

We derive 1-s vertical TEC (VTEC) measurements from phase and pseudorange data collected by the GPS receiver at Fairbanks, Alaska (FAIR) during 15:06–16:36 UT on July 3, 2013, processed at Jet Propulsion Laboratory using the GipsyX and Global Ionospheric Mapping (GIM) software (Komjathy et al., 2005; Bertiger et al., 2020). Phase-based TEC measurements are leveled using pseudorange delays for each phase-connected data collection. We focus on links between FAIR and GPS satellites with pseudo random noise (PRN) numbers 40, 43, and 60, referred to as GPS40, GPS43, and GPS60, whose ionospheric pierce points (IPP) at 450 km altitude are within 300 km proximity to FAIR, or IPPs at 150 km are within 100 km proximity to FAIR. The obtained TEC is expressed in TEC units (TECU), i.e., 10^{16} electrons/m^2. The slant TEC is converted to VTEC using the standard mapping function (e.g., Mannucci et al., 1998). Measurements with elevation angles less than 30° are not considered here. The VTEC data are then detrended to get TEC perturbations (dTEC) using a fourth-order Butterworth low-pass filter. We focus on dTEC with wave periods smaller than 25 min.

We use the following datasets from THEMIS-E (Angelopoulos, 2008): electron energy and pitch-angle distributions measured by the Electrostatic Analyzers (ESA) instrument in the energy range of several eV up to 30 keV (McFadden et al., 2008), DC vector magnetic field at spin resolution ($\sim$3 s) measured by the Fluxgate Magnetometers (FGM) (Auster et al., 2008), electric and magnetic field wave spectra within 1 Hz–4 kHz, measured every $\sim$8 s by the Digital Fields Board (DFB), the Electric Field Instrument (EFI), and the search coil magnetometer (SCM) (Le Contel et al., 2008; Bonnell et al., 2008; Cully, Ergun, et al., 2008). Background electron densities are inferred from spacecraft potentials (Bonnell et al., 2008; Nishimura et al., 2013). We also use ground-based magnetometer measurements every 1 s from the College (CMO) site operated by the United States Geological Survey Geomagnetism Program and from the Fort Yukon (FYKN) site operated by the Geophysical Institute at the University of Alaska.

THEMIS observations of electron distributions and wave spectra allow us to calculate the precipitating flux of electrons scattered into the loss cone by whistler-mode waves using quasilinear diffusion theory (Kennel & Engelmann, 1966; Lyons, 1974). For whistler-mode wave normals $\theta < 45°$, we use a validated analytical formula of bounce-averaged electron diffusion coefficients from Artemyev et al. (2013). For small pitch an-

gle α_{eq} approaching the loss cone α_{LC}, the first-order cyclotron resonance provides the main contribution to the bounce-averaged diffusion rate:

$$\langle D_{\alpha_{eq}\alpha_{eq}} \rangle \simeq \frac{\pi B_w^2 \Omega_{ceq} \omega_m}{4\gamma B_{eq}^2 \Delta\omega (p\epsilon_{meq})^{13/9} T(\alpha_{LC}) \cos^2_{\alpha_{LC}}} \times \frac{\Delta\lambda_{R,N}(1 + 3\sin^2\lambda_R)^{7/12}(1-\bar{\omega})}{|\gamma\bar{\omega} - 2\gamma\bar{\omega}^2 + 1||1-\gamma\bar{\omega}|^{4/9}}, \quad (1)$$

with B_w indicating the wave amplitude, ω_m the mean wave frequency, $\Delta\omega$ the frequency width, $\bar{\omega} = \omega_m/\Omega_{ce}$ the normalized frequency, Ω_{ce} and Ω_{ceq} the local and equatorial electron cyclotron frequency, γ the relativistic factor, p the electron momentum, $\epsilon_{meq} = \Omega_{pe}/\Omega_{ceq}\sqrt{\omega_m/\Omega_{ceq}}$ where Ω_{pe} is the plasma frequency, $T(\alpha_{eq})$ the bounce period, λ_R the latitude of resonance, and $\Delta\lambda_{R,N}$ the latitudinal range of resonance (see details in Artemyev et al. (2013)). The precipitating differential energy flux within the loss cone can be estimated as $x(E)J(E, \alpha_{LC})$, where

$$x(E) = 2\int_0^1 I_0(Z_0\tau)\tau d\tau / I_0(Z_0), \quad (2)$$

being the index of loss cone filling, $J(E, \alpha_{LC})$ is the electron differential energy flux near the loss cone, I_0 is the modified Bessel function with an argument $Z_0 \simeq \alpha_{LC}/\sqrt{\langle D_{\alpha_{eq}\alpha_{eq}}\rangle \cdot \tau_{loss}}$ (Kennel & Petschek, 1966), and τ_{loss} is assumed to be half of the bounce period.

With an energy distribution of precipitating electrons within 0.1–30 keV, we estimate the impact ionization rate altitude profile using the parameterization model developed by Fang et al. (2010), covering isotropic electron precipitation from 100 eV up to 1 MeV. This model, derived through fits to first-principle model results, allows efficient ionization computation for arbitrary energy spectra. Atmospheric density and scale height data were obtained from the NRLMSISE-00 model (Picone et al., 2002). We model TEC perturbations resulting from whistler-induced electron precipitation by integrating ionization rates over altitude and time, adopting an 8-s integration period to align with the temporal resolution of THEMIS wave spectra data. Although our analysis does not concern equilibrium densities and omits recombination and convective effects, this neglect has little impact because we focus on relative TEC perturbations due to short-time precipitation. It takes nearly 60 s for the background ionosphere to relax to an equilibrium density solution for 10-keV precipitation and longer for lower energies (e.g., Kaeppler et al., 2022). Our estimated dTEC also closely match observed dTEC values, underscoring the effectiveness of our modeling approach despite its approximation.

3 Results

On July 3, 2013, from 15:06 to 16:36 UT, the THEMIS-E spacecraft flew westward over the FAIR GPS receiver station, coming within ~20 km relative to FAIR when mapped to 450 km altitude. This optimally timed and positioned space-ground conjunction offers a unique opportunity to link between magnetospheric and ionospheric processes along the field line. The event occurred at L ~7, outside the plasmapause of L_{pp} ~5.4 (based on THEMIS-E densities near 17:00 UT), near the magnetic local time (MLT) of 4.5 hr, and during a geomagnetic quiet time with Kp ~1 and AE ~200 nT. Figure 2a illustrates the trajectories of THEMIS-E and the ionosphere pierce points (IPPs) of GPS40, GPS43, and GPS60 near FAIR, mapped to 450 km altitude. The footprints of THEMIS-E are field-line traced using the Tsyganenko T96 model (Tsyganenko, 1995) but the GPS satellites are mapped using line of sight. Of these GPS satellites, the GPS43 IPPs, moving eastward, were nearest to both the FAIR and THEMIS-E footprints, exhibiting close longitudinal alignment. A notable conjugacy, marked by the bright red segment from 15:37 to 16:11 UT, occurred when the footprints of THEMIS-E and GPS43 IPPs were within ~100 km to each other and FAIR (Figure 2j). In Supporting Information, we also present the configuration when the satellites and their IPPs are mapped to an altitude of 150 km. This adjustment does not significantly alter the geometry of our conjunction event, but it does slightly reduce the scale of the satellite footpaths near FAIR.

Figures 2b–2d present THEMIS observations of whistler-mode waves. The observed wave frequencies were in the whistler lower band, spanning ~0.2–0.5Ω_{ce}, with a mean frequency ω_m ~0.35Ω_{ce}, and $\Delta\omega$ ~0.15Ω_{ce}, where the electron cyclotron frequency f_{ce} ~ $\Omega_{ce}/2\pi$ ~2.15 kHz. Figure 2d shows that whistler-mode wave amplitudes B_w range from several pT to over 100 pT, measured at 8-s cadence (black curve) and smoothed with 2-min moving averages (red curve). Short-term oscillations in B_w on the order of tens of seconds were observed atop more gradual variations of several minutes. We mainly use smoothed or average B_w to estimate electron precipitation in the following. Absent direct waveform data on whistler-mode wave normals for our event, we infer, based on the measured E/cB spectra ($E/cB \ll 1$, see Supporting Information) and statistical nightside equatorial plasma sheet observations (W. Li, Bortnik, Thorne, & Angelopoulos, 2011; Agapitov et al., 2013; Meredith et al., 2021), the presence of quasi-parallel whistlers with an assumed Gaussian wave normal width of $\Delta\theta$ ~30° confined within ±30° in latitude.

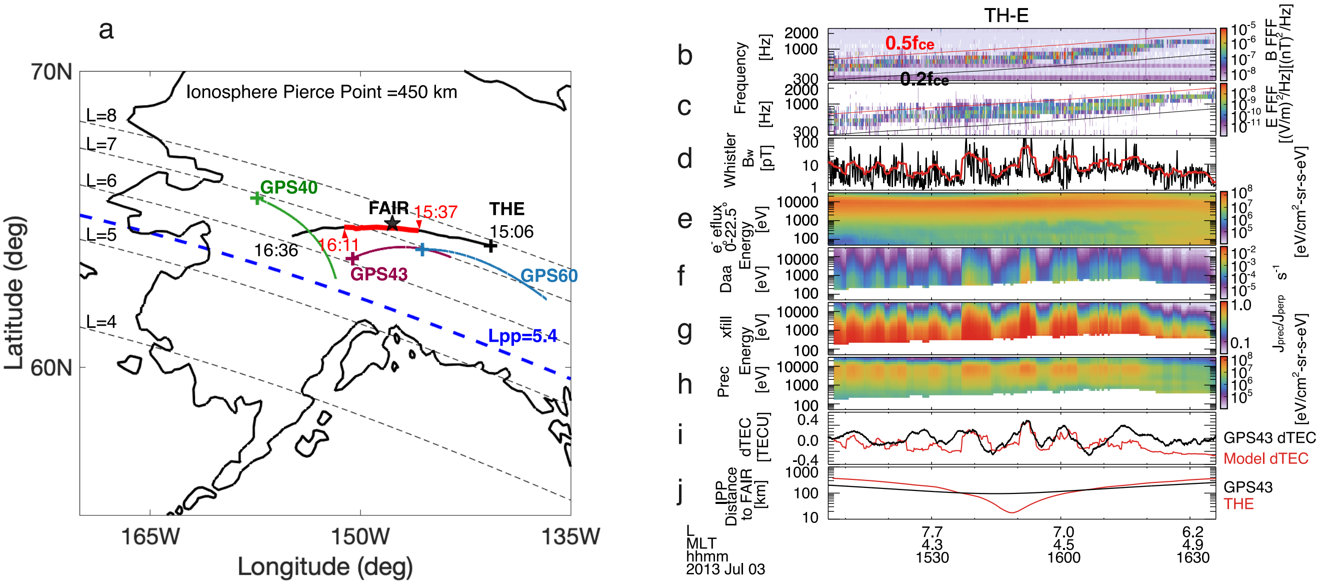

Figure 2. (a) Configuration of THEMIS-E (black curve), GPS40, GPS43, and GPS60 satellites (green, purple, and blue curves), and the FAIR receiver (black star) in geographic coordinates, with THEMIS and GPS mapped onto 450 km altitude using T96 field tracing (THEMIS) or line of sight (GPS). The plus symbol indicates the start of the footpath. (b–e) THEMIS-E magnetic field spectrogram, electric field spectrogram, whistler-mode wave amplitudes, and field-aligned (0°–22.5°) electron energy spectrogram. (f) Bounce-averaged electron diffusion rates. (g) Index of loss cone filling. (h) Whistler-driven precipitating electron energy spectrogram. (i) Comparison of whistler-driven model dTEC (red curve) and GPS43-observed dTEC (black curve). (j) Great-circle distances between THEMIS-E footpath (red curve) and GPS43 raypath (black curve) at IPP of 450 km relative to the FAIR station.

Figures 2e–2h display the measured plasma sheet field-aligned ($\alpha \sim$[0°, 22.5°]) electrons from 50 eV up to 25 keV, calculated diffusion rates $\langle D_{\alpha_{eq}\alpha_{eq}} \rangle$, estimated loss cone filling $x(E)$, and precipitating electron energy fluxes. Although $\langle D_{\alpha_{eq}\alpha_{eq}} \rangle$ and $x(E)$ increase at lower energies, the precipitating energy fluxes peak between 1-10 keV, exhibiting similar modulations as seen in the smoothed whistler-mode wave amplitude B_w. Electron precipitation fluxes below ∼200 eV are absent due to an energy threshold for electron cyclotron resonance interaction, with the lower limit primarily determined by the ratio Ω_{pe}/Ω_{ce} (∼3 in our case).

Figure 2i compares modeled (red) and directly measured dTEC (black) from the GPS43 signal, revealing a nearly one-to-one phase correlation from 15:37 to 16:11 UT. This period of close correlation coincides with the conjunction of THEMIS-E, GPS43, and FAIR, where their relative distances were within ∼100 km (Figure 2j). Outside this conjugacy period and further away from the FAIR station, the correlation decreases. Observed peak-to-peak amplitudes of dTEC reached ∼0.5 TECU. Note that this particular event occurred during quiet conditions; other events during storms may have much larger TEC modulation amplitudes (e.g., Watson et al., 2015), though more challenging to have such reliable conjunction, especially given uncertainties in magnetic field mapping during storms (e.g., C.-L. Huang et al., 2008).

Figure 3 underscores the critical role of observation geometry and timing in detecting phase correlations between modeled and measured dTEC across three GPS satellites. Despite all three satellites having raypath elevation angles >40°—reducing the likelihood of multi-path effects (e.g., Kintner et al., 2007)—only the GPS43 elevation reached 80° above the FAIR station zenith (Figure 3a). During the conjugacy period, the IPPs of GPS40 and GPS60 were distanced from FAIR by more than 200 km, while GPS43's IPPs remained within 100 km, coming within 20 km at its closest point (Figure 3b). Figures 3c and 3d reveal that the modeled dTEC (red curve) aligns poorly with GPS40 and GPS60 dTEC (blue and magenta curves), but a significant cross-correlation (∼0.8) emerges with GPS43 dTEC (black) during the conjugacy period. Before and after the conjunction, TEC phase shifts reduce the cross-correlation to -0.15 and 0.68, respectively. Given the near-parallel longitudinal alignment of GPS43 IPPs and THEMIS-E footprints (Figure 2a), the measured dTEC (black) potentially reflects both temporal and spatial/longitudinal modulations. These findings suggest that to reliably identify the electron precipitation

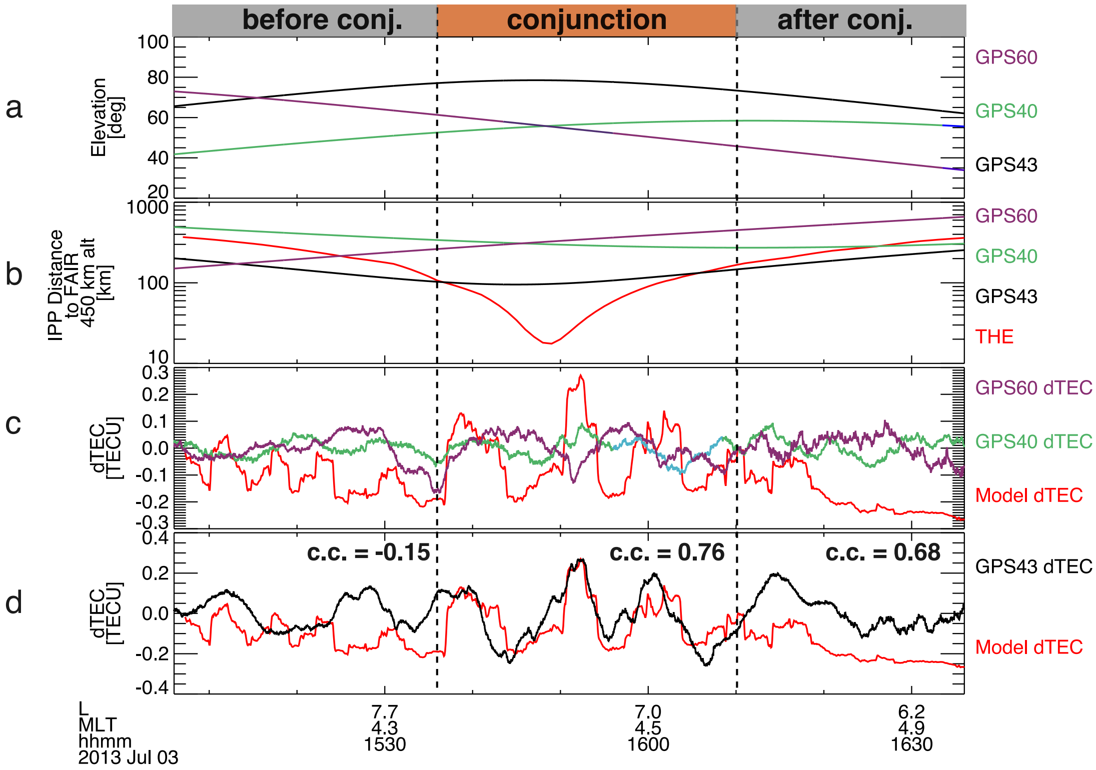

Figure 3. (a) Raypath elevation angles of GPS40 (green curve), GPS43 (black curve), and GPS60 (magenta curve). (b) Distances between THEMIS-E footpath and GPS satellite IPPs relative to FAIR, displayed in the same format as Figure 1j. (c) Comparison between whistler-driven model dTEC and observed dTEC from GPS40 and GPS60, which were not in good conjunction with THEMIS or FAIR. (d) Comparison between whistler-driven model dTEC and GPS43-observed dTEC. The cross-correlation coefficients are -0.15, 0.76, and 0.68 during intervals before, during, and after conjunction, respectively.

responsible for TEC perturbations requires precise spacecraft spatial alignment, optimal timing, and high raypath elevations.

The modulation of TEC perturbations, electron precipitation, and whistler-mode wave amplitudes was linked to ULF wave activities in the Pc3-5 band (1.7 mHz to 100 mHz). Figure 4a display the magnetic field perturbations measured by THEMIS-E in the mean field-aligned (MFA) coordinates, in which the parallel direction ($||$, the compressional component) is determined by 15-minute sliding averages of the magnetic field, the azimuthal direction (ϕ, the toroidal component) is along the cross product of z and the spacecraft geocentric position vector, and the radial direction (r, the poloidal component) completes the triad. Magnetic perturbations are obtained by subtracting the 15-minute mean field. During the conjunction, THEMIS-E detected both compressional Pc5 waves (red curve) and poloidal Pc3-4 waves (blue curve). Figure 4b indicates that peaks in whistler-mode wave amplitudes approximately align with troughs of compressional ULF waves, with fine-scale whistler amplitudes primarily modulated by poloidal Pc3-4 waves (See Supporting Information). Strong Pc5 ULF waves were also recorded in the H-component magnetic field perturbations from magnetometers located at CMO and FYKN (Figures 4g–4h), displaying a similar pattern but with greater amplitudes at FYKN, located slightly north of FAIR. The discrepancy between ground- and space-measured Pc5 waves potentially results from the localized nature of THEMIS-E observations (X. Shi et al., 2022) and the screening/modification effects of ULF waves traversing the ionosphere (Hughes & Southwood, 1976; Lysak, 1991; Lessard & Knudsen, 2001; X. Shi et al., 2018). These observations imply that the ionospheric TEC perturbations were linked to ULF-modulated whistler-mode waves and the associated electron precipitation (e.g., Coroniti & Kennel, 1970; W. Li, Thorne, et al., 2011; Xia et al., 2016; X. J. Zhang et al., 2020; L. Li et al., 2023).

Figures 4b–4c compare small-scale/high-frequency fluctuations of whistler-mode wave amplitudes B_w and dTEC, which was bandpass-filtered within the frequency range of 5–200 mHz. The small-scale dTEC fluctuations exhibit similar wave periods to B_w fluctuations, evidently intensifying during the conjugacy period, yet lacking a clear phase correlation seen with larger scale perturbations in Figure 3d. Assumed purely spatial, small-scale dTEC variations have wavelengths of $\sim$15–30 km at IPPs of 450 km altitude (or $\sim$5–10 km at IPPs of 150 km altitude), compared with the larger-scale dTEC wavelengths of $\sim$80 km (or $\sim$25 km at IPPs of 150 km altitude). When mapped to the mag-

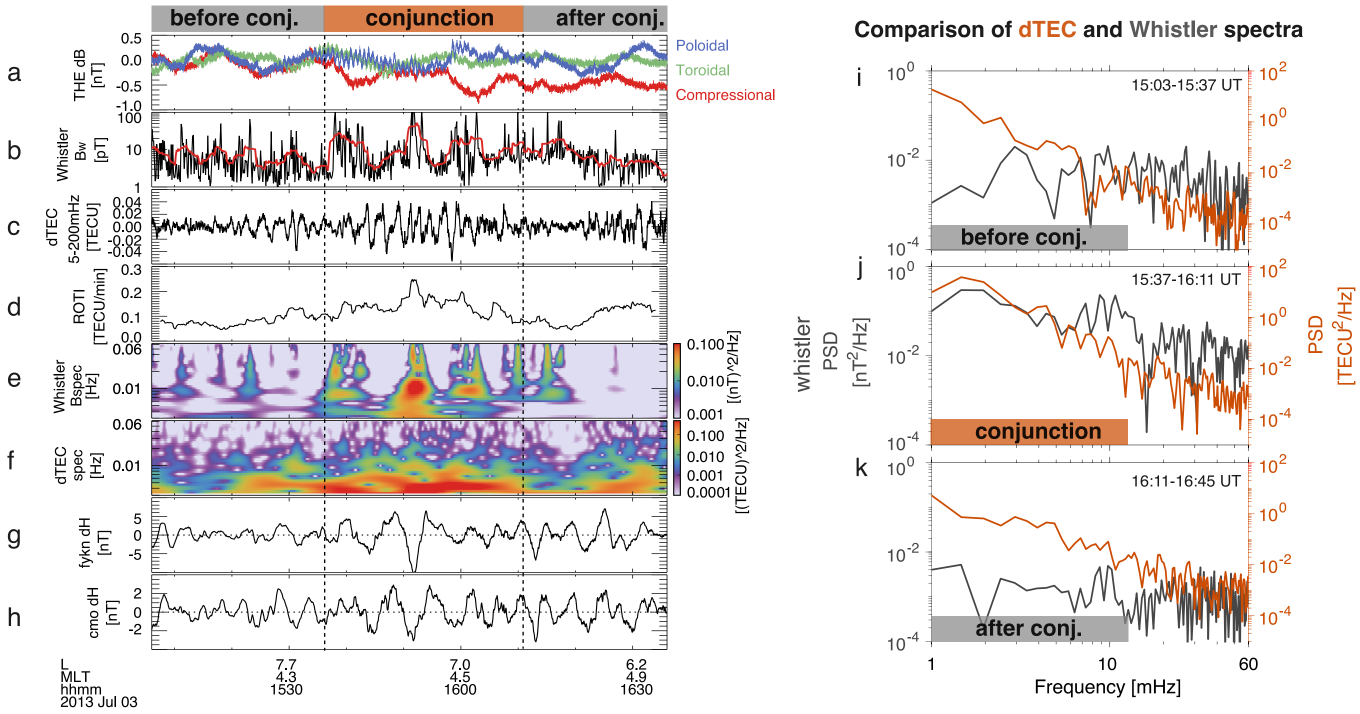

Figure 4. (a) THEMIS-E magnetic field perturbations in the mean-field-aligned (MFA) coordinates, exhibiting primarily compressional- (red) and poloidal-mode (blue) variations. (b) THEMIS-E whistler-mode wave amplitudes. The measured amplitudes are shown in black and smoothed in red. (c) dTEC bandpass filtered within 5–200 mHz. (d) The rate of dTEC changes ($ROTI$) from 200-s sliding window ensemble averaging. (e) Wavelet spectrogram of whistler-mode waves. (f) Wavelet spectrogram of GPS43 dTEC. (g) Ground-based magnetic field H component perturbations in 1.7–100 mHz from the Fort Yukon station (FYKN). (h) Ground-based magnetic H component perturbations in 1.7–100 mHz from the College station (CMO). (i–k) Comparisons of dTEC (orange curves) and whistler-mode wave amplitude fluctuation spectra (gray curves) in 1–60 mHz measured before (k), during (j), and after (k) the conjugacy period.

netosphere, these small-scale dTEC modulations correspond to a magnetospheric source region of ∼100–700 km, while large-scale dTEC modulations suggest a source region of ∼500–2000 km. These scales align with prior observations of the transverse scale sizes of chorus elements and their source regions (SantolíK et al., 2003; Agapitov et al., 2017, 2018), and with the azimuthal wavelengths of high-m poloidal ULF waves (Yeoman et al., 2012; X. Shi et al., 2018; Zong et al., 2017). Figure 4d shows the rate of TEC index ($ROTI$), i.e., the standard deviation of the rate of TEC (ROT) (Pi et al., 1997), where $ROT = (dTEC(t+\tau) - dTEC(t))/\tau$ with τ =10 s, $ROTI = \sqrt{\langle ROT^2\rangle - \langle ROT\rangle^2}$ using 200-s sliding averages. Significant increases in $ROTI$ were observed within the region of whistler-driven TEC perturbations. However, in our case the GPS signal fluctuations were predominantly refractive, as negligible fluctuations were detected at frequencies above 0.1 Hz (McCaffrey & Jayachandran, 2017, 2019; Nishimura et al., 2023).

Figures 4e–4f compare the wavelet spectrograms of whistler-mode wave B_w and dTEC, displaying concurrent increases in wave power for both in the frequency range of ∼3 mHz up to tens of mHz. Figures 4i–4k present a more detailed amplitude spectra comparison before, during, and after conjunction. Notably, only during the conjunction, whistler-mode wave amplitudes and dTEC share similar power spectral density (PSD) distributions in the 1–∼30 mHz range. The peaks in whistler spectra were slightly and consistently larger than those in dTEC spectra within 3–20 mHz by factors of 1.05–1.2, aligning with expected Doppler shift effects on ionospheric TEC measurements. The Doppler shift results from relative motion of GPS raypath (or IPPs with velocities of ∼160 m/s in our case) and propagating TEC structures (typically with velocities of several hundred m/s) (Watson, Jayachandran, & MacDougall, 2016): $f_{cor} = f_{obs}(1+\frac{\mathbf{v_{ipp}}\cdot\mathbf{v_{struct}}}{|\mathbf{v_{struct}}|^2})$, where f_{cor} is the frequency corrected for relative motion. Watson, Jayachandran, and MacDougall (2016) found that 89% of their statistical events required a correction factor of 1.2 or less for the Doppler shift, consistent with our observations. The agreement between dTEC and whistler amplitude spectra supports that the observed dTEC resulted from electron precipitation induced by whistler-mode waves.

Figure 5 indicates that the electron precipitation, induced by ULF-modulated whistler-mode waves, can cause significant increases in ionospheric ionization rate or column density, leading to TEC perturbations of ∼0.36 TECU with a moderate whistler amplitude of B_w ∼25 pT. Given that large-amplitude whistler-mode waves exceeding several hundred pT frequently occur in the inner magnetosphere (Cattell et al., 2008; Cully, Bon-

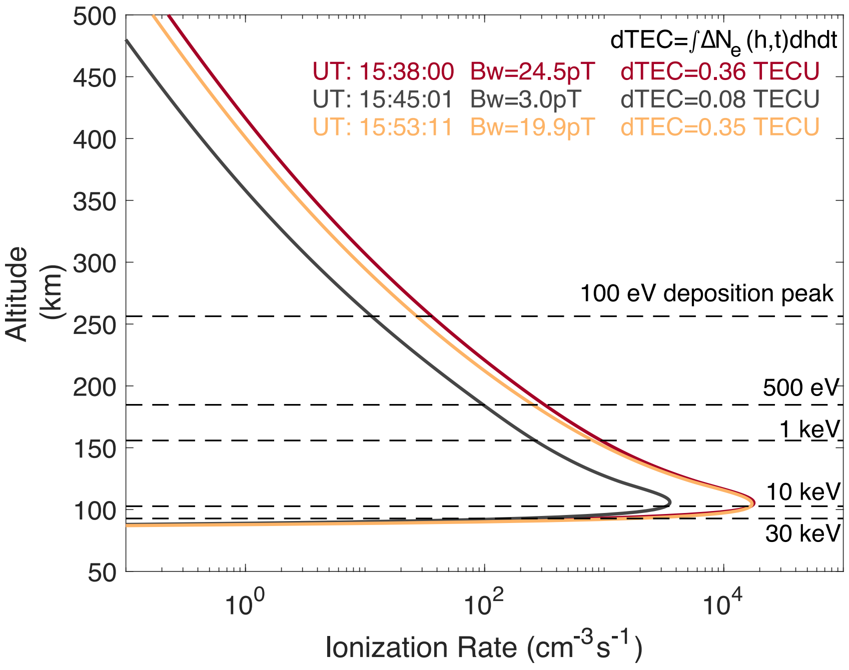

Figure 5. Ionization rate altitude profiles calculated at three time stamps of 15:38:00, 15:45:01, and 15:53:11 UT, corresponding to whistler-mode wave amplitudes of B_w= 24.5 pT (red curve), 3.0 pT (gray curve), and 19.9 pT (orange curve). The dTEC were calculated by integrating ionization rates over altitude and time (8s). The dashed lines mark the peak deposition altitudes of 100 eV, 500 eV, 1 keV, 10 keV, and 30 keV precipitating monoenergetic electrons.

nell, & Ergun, 2008; Agapitov et al., 2014; Hartley et al., 2016; R. Shi et al., 2019), we anticipate even larger TEC perturbations from such whistler activities. We defer a statistical study including storm time events and the potential connection with scintillation (e.g., McCaffrey & Jayachandran, 2019; Nishimura et al., 2023) for the future. In addition, the primary energy range of precipitation spans from ∼100 eV to ∼30 keV, contributing to density variations between ∼90–∼400 km (Fang et al., 2010; Katoh et al., 2023; Berland et al., 2023).

4 Discussion

Various mechanisms have been proposed that link ULF waves to TEC perturbations and ionospheric disturbances in general (Pilipenko et al., 2014). Although TEC perturbations might arise from direct ULF wave effects through convective and divergent flows, MHD Alfvén-mode waves do not directly alter plasma density. Furthermore, mode-converted compressional waves, if present due to Hall currents, are evanescent in the ionosphere (Lessard & Knudsen, 2001), resulting in negligible TEC perturbations (Pilipenko et al., 2014), especially when the ground-based magnetic perturbations were only several nT in our case (Figure 4g). A non-linear "feedback instability" mechanism may modify ULF wave dynamics, causing field-aligned current striations and significant bottom-side ionospheric density cavities and gradients (Lysak, 1991; Streltsov & Lotko, 2008). Furthermore, ULF-induced plasma flows may result in gradient drift instabilities and density striations and irregularities with scale sizes less than ∼10 km, in the presence of pre-existing larger-scale density gradients (Keskinen & Ossakow, 1983; Basu et al., 1990; Gondarenko & Guzdar, 2004; Kelley, 2009; Spicher et al., 2015; Nishimura et al., 2021). Additionally, electron precipitation and Joule heating are important factors to consider in the auroral region (e.g., Deng & Ridley, 2007; Sheng et al., 2020; Meng et al., 2022).

Detecting one-to-one phase correlation between ground-based ULF waves and TEC perturbations may be challenging, largely due to ionospheric screening effects on ULF waves (Hughes & Southwood, 1976), with only a few exceptions noted during storm times (Pilipenko et al., 2014; Wang et al., 2020). However, this correlation has been frequently observed with spacecraft measurements of ULF waves (Watson et al., 2015; Watson, Jayachandran, Singer, et al., 2016; Zhai et al., 2021), indicating that magnetospheric processes may play an important role in driving ionospheric TEC perturbations. Our findings support that magnetospheric whistler-mode waves, modulated by ULF waves in the

Pc3–5 band, are responsible for these periodic TEC perturbations through associated electron precipitation.

These results enhance our understanding of TEC modulation by ULF waves, a topic widely discussed in the literature (Skone, 2009; Pilipenko et al., 2014; Watson et al., 2015; Watson, Jayachandran, Singer, et al., 2016; Wang et al., 2020; Zhai et al., 2021), and facilitates the integration of effects of magnetospheric whistler-mode waves into auroral TEC models. Statistical modeling of whistler-mode and ULF waves has been improving for several decades (e.g., Tsurutani & Smith, 1974; McPherron, 1972; Southwood & Hughes, 1983; Takahashi & Anderson, 1992; M. Hudson et al., 2004; Claudepierre et al., 2010; W. Li, Bortnik, Thorne, & Angelopoulos, 2011; Agapitov et al., 2013; Artemyev et al., 2016; Tyler et al., 2019; Zong et al., 2017; Ma et al., 2020; X. J. Zhang et al., 2020; Sandhu et al., 2021; Hartinger et al., 2015, 2022, 2023). Leveraging these wave effects and the associated electron precipitation can enhance physics-based modeling of ionospheric TEC by providing better specifications of high-latitude drivers (Schunk et al., 2004; Ridley et al., 2006; Zettergren & Snively, 2015; Meng et al., 2016, 2020; Sheng et al., 2020; Verkhoglyadova et al., 2020; Huba & Drob, 2017). This wave-driven precipitation provides the dominant energy input to the ionosphere among all types of auroral precipitation (e.g., Newell et al., 2009), thus critically contributing to TEC perturbations at high latitudes. As such, incorporating these magnetospheric phenomena is vital for improving the accuracy of ionospheric TEC models. This incorporation potentially benefits both GNSS-based applications and magnetosphere and ionosphere coupling science.

5 Conclusions

We present a detailed case study of ionospheric TEC perturbations (dTEC), using magnetically-conjugate observations from the THEMIS spacecraft and the GPS receiver at Fairbanks, Alaska (FAIR). This conjunction setup allows us to identify the magnetospheric driver of the observed dTEC. Our key findings are summarized below:

- Combining in-situ wave and electron observations and quasilinear theory, we have modeled the electron precipitation induced by observed whistler-mode waves and deduced ionospheric dTEC based on impact ionization prediction. The cross-correlation

between our modeled and observed dTEC reached ∼0.8 during the conjugacy period of ∼30 min but decreased outside of it.

- Observed peak-to-peak dTEC amplitudes reached ∼0.5 TECU, exhibiting modulations spanning scales of ∼5–80 km. Within the modulated dTEC, enhancements in the rate of TEC index (ROTI) were measured to be ∼0.2 TECU/min.
- The whistler-mode waves and dTEC modulations were linked to ULF waves in the Pc3-5 band, featuring concurrent compressional and poloidal mode fluctuations. The amplitude spectra of whistler-mode waves and dTEC also agreed from 1 mHz to tens of mHz during the conjugacy period but diverged outside of it.

Thus, our results provide direct evidence that ULF-modulated whistler-mode waves in the magnetosphere drive electron precipitation leading to ionospheric dTEC modulations. Our observations also indicate that to reliably identify the electron precipitation responsible for TEC perturbations requires precise spacecraft spatial alignment, optimal timing, and high raypath elevations. Our findings elucidate the high-latitude dTEC generation from magnetospheric wave-induced precipitation, which has not been adequately addressed in physics-based TEC models. Consequently, theses results improve ionospheric TEC prediction and enhance our understanding of magnetosphere-ionosphere coupling via ULF waves.

Acknowledgments

This work has been supported by NASA projects 80NSSC23K0413, 80NSSC24K0138, and 80NSSC23K1038. Portions of the research were carried out at the Jet Propulsion Laboratory, California Institute of Technology, under a contract with NASA. O.P.V., M.D.H., and X.S. were supported by NASA project 80NSSC21K1683. X.S. is supported by NASA award 80NSSC21K1677. O.P.V would like to thank A.W. Moore (JPL) for data processing discussions. We are indebted to Emmanuel Masongsong for help with the schematic figure.

Open Research

THEMIS data are available at `http://themis.ssl.berkeley.edu/data/themis/the/l2/`. GPS RINEX data are publicly available from the NASA CDDIS archive of space geodesy data (`https://cddis.nasa.gov/Data_and_Derived_Products/GNSS/high-rate`

_data.html). TEC data derived for this study is available at https://doi.org/10.48577/jpl.LGI5JS (Verkhoglyadova, 2024). The access and processing of THEMIS and ground-based magnetic field data from CMO and FYKN was done using SPEDAS V4.1, see Angelopoulos et al. (2019). The original CMO data are provided by the USGS Geomagnetism Program (http://geomag.usgs.gov) but can be accessed through http://themis.ssl.berkeley.edu/data/themis/thg/l2/mag/cmo/2013/. FYKN data are part of the Geophysical Institute Magnetometer Array operated by the Geophysical Institute, University of Alaska (https://www.gi.alaska.edu/monitors/magnetometer/archive).

References

Aa, E., Zou, S., Eastes, R., Karan, D. K., Zhang, S.-R., Erickson, P. J., & Coster, A. J. (2020, January). Coordinated Ground-Based and Space-Based Observations of Equatorial Plasma Bubbles. *Journal of Geophysical Research (Space Physics)*, *125*(1), e27569. doi: 10.1029/2019JA027569

Agapitov, O. V., Artemyev, A., Krasnoselskikh, V., Khotyaintsev, Y. V., Mourenas, D., Breuillard, H., ... Rolland, G. (2013, June). Statistics of whistler mode waves in the outer radiation belt: Cluster STAFF-SA measurements. *J. Geophys. Res.*, *118*, 3407-3420. doi: 10.1002/jgra.50312

Agapitov, O. V., Artemyev, A., Mourenas, D., Krasnoselskikh, V., Bonnell, J., Le Contel, O., ... Angelopoulos, V. (2014). The quasi-electrostatic mode of chorus waves and electron nonlinear acceleration. *J. Geophys. Res.*, *119*, 1606–1626. doi: 10.1002/2013JA019223

Agapitov, O. V., Blum, L. W., Mozer, F. S., Bonnell, J. W., & Wygant, J. (2017, March). Chorus whistler wave source scales as determined from multipoint Van Allen Probe measurements. *Geophys. Res. Lett.*, *44*, 2634-2642. doi: 10.1002/2017GL072701

Agapitov, O. V., Mourenas, D., Artemyev, A., Mozer, F. S., Bonnell, J. W., Angelopoulos, V., ... Krasnoselskikh, V. (2018, October). Spatial Extent and Temporal Correlation of Chorus and Hiss: Statistical Results From Multipoint THEMIS Observations. *Journal of Geophysical Research (Space Physics)*, *123*(10), 8317-8330. doi: 10.1029/2018JA025725

Angelopoulos, V. (2008, December). The THEMIS Mission. *Space Sci. Rev.*, *141*, 5-34. doi: 10.1007/s11214-008-9336-1

Angelopoulos, V., Cruce, P., Drozdov, A., Grimes, E. W., Hatzigeorgiu, N., King, D. A., ... Schroeder, P. (2019, January). The Space Physics Environment Data Analysis System (SPEDAS). *Space Sci. Rev.*, *215*, 9. doi: 10.1007/s11214-018-0576-4

Artemyev, A. V., Agapitov, O., Mourenas, D., Krasnoselskikh, V., Shastun, V., & Mozer, F. (2016, April). Oblique Whistler-Mode Waves in the Earth's Inner Magnetosphere: Energy Distribution, Origins, and Role in Radiation Belt Dynamics. *Space Sci. Rev.*, *200*(1-4), 261-355. doi: 10.1007/s11214-016-0252-5

Artemyev, A. V., Mourenas, D., Agapitov, O. V., & Krasnoselskikh, V. V. (2013, April). Parametric validations of analytical lifetime estimates for radiation belt electron diffusion by whistler waves. *Annales Geophysicae*, *31*, 599-624. doi: 10.5194/angeo-31-599-2013

Astafyeva, E. (2019, December). Ionospheric Detection of Natural Hazards. *Reviews of Geophysics*, *57*(4), 1265-1288. doi: 10.1029/2019RG000668

Auster, H. U., Glassmeier, K. H., Magnes, W., Aydogar, O., Baumjohann, W., Constantinescu, D., ... Wiedemann, M. (2008, December). The THEMIS Fluxgate Magnetometer. *Space Sci. Rev.*, *141*, 235-264. doi: 10.1007/s11214-008-9365-9

Basu, S., Groves, K. M., Basu, S., & Sultan, P. J. (2002, November). Specification and forecasting of scintillations in communication/navigation links: current status and future plans. *Journal of Atmospheric and Solar-Terrestrial Physics*, *64*(16), 1745-1754. doi: 10.1016/S1364-6826(02)00124-4

Basu, S., MacKenzie, E., Basu, S., Coley, W. R., Sharber, J. R., & Hoegy, W. R. (1990, June). Plasma structuring by the gradient drift instability at high latitudes and comparison with velocity shear driven processes. *J. Geophys. Res.*, *95*(A6), 7799-7818. doi: 10.1029/JA095iA06p07799

Berland, G. D., Marshall, R. A., Capannolo, L., McCarthy, M. P., & Zheng, L. (2023, November). Kinetic Modeling of Radiation Belt Electrons With Geant4 to Study Energetic Particle Precipitation in Earth's Atmosphere. *Earth and Space Science*, *10*(11), e2023EA002987. doi: 10.1029/2023EA002987

Bertiger, W., Bar-Sever, Y., Dorsey, A., Haines, B., Harvey, N., Hemberger, D., ... Willis, P. (2020, August). GipsyX/RTGx, a new tool set for space geodetic operations and research. *Advances in Space Research*, *66*(3), 469-489. doi:

10.1016/j.asr.2020.04.015

Bonnell, J. W., Mozer, F. S., Delory, G. T., Hull, A. J., Ergun, R. E., Cully, C. M., ... Harvey, P. R. (2008, December). The Electric Field Instrument (EFI) for THEMIS. *Space Sci. Rev.*, *141*, 303-341. doi: 10.1007/s11214-008-9469-2

Cattell, C., Wygant, J. R., Goetz, K., Kersten, K., Kellogg, P. J., von Rosenvinge, T., ... Russell, C. T. (2008, January). Discovery of very large amplitude whistler-mode waves in Earth's radiation belts. *Geophys. Res. Lett.*, *35*, 1105. doi: 10.1029/2007GL032009

Cherniak, I., Krankowski, A., & Zakharenkova, I. (2014, August). Observation of the ionospheric irregularities over the Northern Hemisphere: Methodology and service. *Radio Science*, *49*(8), 653-662. doi: 10.1002/2014RS005433

Ciraolo, L., Azpilicueta, F., Brunini, C., Meza, A., & Radicella, S. M. (2007). Calibration errors on experimental slant total electron content (TEC) determined with GPS. *Journal of Geodesy*, *81*, 111–120.

Claudepierre, S. G., Hudson, M. K., Lotko, W., Lyon, J. G., & Denton, R. E. (2010, November). Solar wind driving of magnetospheric ULF waves: Field line resonances driven by dynamic pressure fluctuations. *Journal of Geophysical Research (Space Physics)*, *115*(A11), A11202. doi: 10.1029/2010JA015399

Coroniti, F. V., & Kennel, C. F. (1970, March). Electron precipitation pulsations. *J. Geophys. Res.*, *75*(7), 1279-1289. doi: 10.1029/JA075i007p01279

Coster, A. J., & Yizengaw, E. (2021). Gnss/gps degradation from space weather. In *Space weather effects and applications* (p. 165-181). American Geophysical Union (AGU). doi: https://doi.org/10.1002/9781119815570.ch8

Cully, C. M., Bonnell, J. W., & Ergun, R. E. (2008, June). THEMIS observations of long-lived regions of large-amplitude whistler waves in the inner magnetosphere. *Geophys. Res. Lett.*, *35*, 17. doi: 10.1029/2008GL033643

Cully, C. M., Ergun, R. E., Stevens, K., Nammari, A., & Westfall, J. (2008, December). The THEMIS Digital Fields Board. *Space Sci. Rev.*, *141*, 343-355. doi: 10.1007/s11214-008-9417-1

Davies, K., & Hartmann, G. K. (1976, July). Short-period fluctuations in total columnar electron content. *J. Geophys. Res.*, *81*(19), 3431. doi: 10.1029/JA081i019p03431

Deng, Y., & Ridley, A. J. (2007, September). Possible reasons for underestimat-

ing Joule heating in global models: E field variability, spatial resolution, and vertical velocity. *Journal of Geophysical Research (Space Physics)*, *112*(A9), A09308. doi: 10.1029/2006JA012006

Fæhn Follestad, A., Herlingshaw, K., Ghadjari, H., Knudsen, D. J., McWilliams, K. A., Moen, J. I., ... Oksavik, K. (2020, June). Dayside Field-Aligned Current Impacts on Ionospheric Irregularities. *Geophys. Res. Lett.*, *47*(11), e86722. doi: 10.1029/2019GL086722

Fang, X., Randall, C. E., Lummerzheim, D., Wang, W., Lu, G., Solomon, S. C., & Frahm, R. A. (2010, November). Parameterization of monoenergetic electron impact ionization. *Geophys. Res. Lett.*, *37*(22), L22106. doi: 10.1029/2010GL045406

Gondarenko, N. A., & Guzdar, P. N. (2004, September). Plasma patch structuring by the nonlinear evolution of the gradient drift instability in the high-latitude ionosphere. *Journal of Geophysical Research (Space Physics)*, *109*(A9), A09301. doi: 10.1029/2004JA010504

Hapgood, M., Liu, H., & Lugaz, N. (2022, March). SpaceX—Sailing Close to the Space Weather? *Space Weather*, *20*(3), e2022SW003074. doi: 10.1029/2022SW00307410.1002/essoar.10510636.1

Hartinger, M. D., Elsden, T., Archer, M. O., Takahashi, K., Wright, A. N., Artemyev, A., ... Angelopoulos, V. (2023, December). Properties of Magnetohydrodynamic Normal Modes in the Earth's Magnetosphere. *Journal of Geophysical Research (Space Physics)*, *128*(12), e2023JA031987. doi: 10.1029/2023JA031987

Hartinger, M. D., Moldwin, M. B., Zou, S., Bonnell, J. W., & Angelopoulos, V. (2015, January). ULF wave electromagnetic energy flux into the ionosphere: Joule heating implications. *Journal of Geophysical Research (Space Physics)*, *120*(1), 494-510. doi: 10.1002/2014JA020129

Hartinger, M. D., Takahashi, K., Drozdov, A. Y., Shi, X., Usanova, M. E., & Kress, B. (2022, April). ULF Wave Modeling, Effects, and Applications: Accomplishments, Recent Advances, and Future. *Frontiers in Astronomy and Space Sciences*, *9*, 867394. doi: 10.3389/fspas.2022.867394

Hartley, D. P., Kletzing, C. A., Kurth, W. S., Bounds, S. R., Averkamp, T. F., Hospodarsky, G. B., ... Watt, C. E. J. (2016, May). Using the cold plasma

dispersion relation and whistler mode waves to quantify the antenna sheath impedance of the Van Allen Probes EFW instrument. *Journal of Geophysical Research (Space Physics)*, *121*(5), 4590-4606. doi: 10.1002/2016JA022501

Hey, J. S., Parsons, S. J., & Phillips, J. W. (1946, August). Fluctuations in Cosmic Radiation at Radio-Frequencies. *Nature*, *158*(4007), 234. doi: 10.1038/158234a0

Huang, C.-L., Spence, H. E., Singer, H. J., & Tsyganenko, N. A. (2008, April). A quantitative assessment of empirical magnetic field models at geosynchronous orbit during magnetic storms. *Journal of Geophysical Research (Space Physics)*, *113*(A4), A04208. doi: 10.1029/2007JA012623

Huang, C.-S., & Kelley, M. C. (1996, January). Nonlinear evolution of equatorial spread F. 2. Gravity wave seeding of Rayleigh-Taylor instability. *J. Geophys. Res.*, *101*(A1), 293-302. doi: 10.1029/95JA02210

Huba, J. D., & Drob, D. (2017, June). SAMI3 prediction of the impact of the 21 August 2017 total solar eclipse on the ionosphere/plasmasphere system. *Geophys. Res. Lett.*, *44*(12), 5928-5935. doi: 10.1002/2017GL073549

Hudson, M., Denton, R., Lessard, M., Miftakhova, E., & Anderson, R. (2004, January). A study of Pc-5 ULF oscillations. *Annales Geophysicae*, *22*(1), 289-302. doi: 10.5194/angeo-22-289-2004

Hudson, M. K., Elkington, S. R., Lyon, J. G., & Goodrich, C. C. (2000, January). Increase in Relativistic Electron Flux in the Inner Magnetosphere: ULF Wave Mode Structure. *Advances in Space Research*, *25*(12), 2327-2337. doi: 10.1016/S0273-1177(99)00518-9

Hudson, M. K., Kress, B. T., Mueller, H.-R., Zastrow, J. A., & Bernard Blake, J. (2008, March). Relationship of the Van Allen radiation belts to solar wind drivers. *Journal of Atmospheric and Solar-Terrestrial Physics*, *70*, 708-729. doi: 10.1016/j.jastp.2007.11.003

Hughes, W. J., & Southwood, D. J. (1976, July). The screening of micropulsation signals by the atmosphere and ionosphere. *J. Geophys. Res.*, *81*(19), 3234. doi: 10.1029/JA081i019p03234

Hunsucker, R. D. (1982, May). Atmospheric Gravity Waves Generated in the High-Latitude Ionosphere: A Review (Paper 1R1822). *Reviews of Geophysics and Space Physics*, *20*, 293. doi: 10.1029/RG020i002p00293

Jacobs, J. A., Kato, Y., Matsushita, S., & Troitskaya, V. A. (1964, January). Classification of Geomagnetic Micropulsations. *J. Geophys. Res.*, *69*, 180-181. doi: 10.1029/JZ069i001p00180

Jakowski, N., Mayer, C., Hoque, M., & Wilken, V. (2011). Total electron content models and their use in ionosphere monitoring. *Radio Science*, *46*(06), 1–11.

Jakowski, N., Sardon, E., Engler, E., Jungstand, A., & Klähn, D. (1996, December). Relationships between GPS-signal propagation errors and EISCAT observations. *Annales Geophysicae*, *14*(12), 1429-1436. doi: 10.1007/s00585-996-1429-0

Jaynes, A. N., Lessard, M. R., Takahashi, K., Ali, A. F., Malaspina, D. M., Michell, R. G., … Wygant, J. R. (2015, October). Correlated Pc4-5 ULF waves, whistler-mode chorus, and pulsating aurora observed by the Van Allen Probes and ground-based systems. *J. Geophys. Res.*, *120*, 8749-8761. doi: 10.1002/2015JA021380

Jin, Y., Moen, J. I., & Miloch, W. J. (2015). On the collocation of the cusp aurora and the GPS phase scintillation: A statistical study. *Journal of Geophysical Research: Space Physics*, *120*(10), 9176–9191.

Jin, Y., Xiong, C., Clausen, L., Spicher, A., Kotova, D., Brask, S., … Miloch, W. (2020, July). Ionospheric Plasma Irregularities Based on In Situ Measurements From the Swarm Satellites. *Journal of Geophysical Research (Space Physics)*, *125*(7), e28103. doi: 10.1029/2020JA028103

Kaeppler, S. R., Marshall, R., Sanchez, E. R., Juarez Madera, D. H., Troyer, R., & Jaynes, A. N. (2022, December). pyGPI5: A python D- and E-region chemistry and ionization model. *Frontiers in Astronomy and Space Sciences*, *9*, 338. doi: 10.3389/fspas.2022.1028042

Katoh, Y., Rosendahl, P. S., Ogawa, Y., Hiraki, Y., & Tadokoro, H. (2023, December). Effect of the mirror force on the collision rate due to energetic electron precipitation: Monte Carlo simulations. *Earth, Planets and Space*, *75*(1), 117. doi: 10.1186/s40623-023-01871-y

Kelley, M. C. (2009). *The Earth's ionosphere: Plasma physics and electrodynamics.* Academic press.

Kennel, C. F., & Engelmann, F. (1966, November). Velocity Space Diffusion from Weak Plasma Turbulence in a Magnetic Field. *Physics of Fluids*, *9*, 2377-2388.

doi: 10.1063/1.1761629

Kennel, C. F., & Petschek, H. E. (1966, January). Limit on Stably Trapped Particle Fluxes. *J. Geophys. Res.*, *71*, 1-28.

Keskinen, M. J., & Ossakow, S. L. (1983, January). Nonlinear evolution of convecting plasma enhancements in the auroral ionosphere, 2. Small scale irregularities. *J. Geophys. Res.*, *88*(A1), 474-482. doi: 10.1029/JA088iA01p00474

Kintner, P. M., Ledvina, B. M., & de Paula, E. R. (2007, September). GPS and ionospheric scintillations. *Space Weather*, *5*(9), 09003. doi: 10.1029/2006SW000260

Komjathy, A. (1997). *Global ionospheric total electron content mapping using the global positioning system* (Unpublished doctoral dissertation). University of New Brunswick, Canada.

Komjathy, A., Sparks, L., Wilson, B. D., & Mannucci, A. J. (2005, December). Automated daily processing of more than 1000 ground-based GPS receivers for studying intense ionospheric storms. *Radio Science*, *40*(6), RS6006. doi: 10.1029/2005RS003279

Komjathy, A., Yang, Y.-M., Meng, X., Verkhoglyadova, O., Mannucci, A. J., & Langley, R. B. (2016, July). Review and perspectives: Understanding natural-hazards-generated ionospheric perturbations using GPS measurements and coupled modeling. *Radio Science*, *51*(7), 951-961. doi: 10.1002/2015RS005910

Le Contel, O., Roux, A., Robert, P., Coillot, C., Bouabdellah, A., de La Porte, B., … Larson, D. (2008, December). First Results of the THEMIS Search Coil Magnetometers. *Space Sci. Rev.*, *141*, 509-534. doi: 10.1007/s11214-008-9371-y

Lessard, M. R., & Knudsen, D. J. (2001, October). Ionospheric reflection of small-scale Alfvén waves. *Geophys. Res. Lett.*, *28*(18), 3573-3576. doi: 10.1029/2000GL012529

Li, L., Omura, Y., Zhou, X.-Z., Zong, Q.-G., Rankin, R., Yue, C., & Fu, S.-Y. (2022, May). Nonlinear Wave Growth Analysis of Chorus Emissions Modulated by ULF Waves. *Geophys. Res. Lett.*, *49*(10), e97978. doi: 10.1029/2022GL097978

Li, L., Omura, Y., Zhou, X.-Z., Zong, Q.-G., Rankin, R., Yue, C., … Ren, J. (2023,

February). Chorus Wave Generation Modulated by Field Line Resonance and Mirror-Mode ULF Waves. *Journal of Geophysical Research (Space Physics)*, *128*(2), e2022JA031127. doi: 10.1029/2022JA031127

Li, W., Bortnik, J., Thorne, R. M., & Angelopoulos, V. (2011, December). Global distribution of wave amplitudes and wave normal angles of chorus waves using THEMIS wave observations. *J. Geophys. Res.*, *116*, 12205. doi: 10.1029/2011JA017035

Li, W., Bortnik, J., Thorne, R. M., Nishimura, Y., Angelopoulos, V., & Chen, L. (2011, June). Modulation of whistler mode chorus waves: 2. Role of density variations. *J. Geophys. Res.*, *116*, A06206. doi: 10.1029/2010JA016313

Li, W., Thorne, R. M., Bortnik, J., Nishimura, Y., & Angelopoulos, V. (2011, June). Modulation of whistler mode chorus waves: 1. Role of compressional Pc4-5 pulsations. *J. Geophys. Res.*, *116*, A06205. doi: 10.1029/2010JA016312

Lyons, L. R. (1974, December). Pitch angle and energy diffusion coefficients from resonant interactions with ion-cyclotron and whistler waves. *Journal of Plasma Physics*, *12*, 417-432. doi: 10.1017/S002237780002537X

Lysak, R. L. (1991, February). Feedback instability of the ionospheric resonant cavity. *J. Geophys. Res.*, *96*(A2), 1553-1568. doi: 10.1029/90JA02154

Ma, Q., Connor, H. K., Zhang, X. J., Li, W., Shen, X. C., Gillespie, D., ... Spence, H. E. (2020, August). Global Survey of Plasma Sheet Electron Precipitation due to Whistler Mode Chorus Waves in Earth's Magnetosphere. *Geophys. Res. Lett.*, *47*(15), e88798. doi: 10.1029/2020GL088798

Makarevich, R. A., Crowley, G., Azeem, I., Ngwira, C., & Forsythe, V. V. (2021, May). Auroral E Region as a Source Region for Ionospheric Scintillation. *Journal of Geophysical Research (Space Physics)*, *126*(5), e29212. doi: 10.1029/2021JA029212

Mannucci, A. J., Wilson, B. D., Yuan, D. N., Ho, C. H., Lindqwister, U. J., & Runge, T. F. (1998, May). A global mapping technique for GPS-derived ionospheric total electron content measurements. *Radio Science*, *33*(3), 565-582. doi: 10.1029/97RS02707

McCaffrey, A. M., & Jayachandran, P. T. (2017, June). Observation of subsecond variations in auroral region total electron content using 100 Hz sampling of GPS observables. *Journal of Geophysical Research (Space Physics)*, *122*(6),

6892-6900. doi: 10.1002/2017JA024255

McCaffrey, A. M., & Jayachandran, P. T. (2019, February). Determination of the Refractive Contribution to GPS Phase "Scintillation". *Journal of Geophysical Research (Space Physics)*, *124*(2), 1454-1469. doi: 10.1029/2018JA025759

McFadden, J. P., Carlson, C. W., Larson, D., Ludlam, M., Abiad, R., Elliott, B., ... Angelopoulos, V. (2008, December). The THEMIS ESA Plasma Instrument and In-flight Calibration. *Space Sci. Rev.*, *141*, 277-302. doi: 10.1007/s11214-008-9440-2

McPherron, R. L. (1972, September). Substorm related changes in the geomagnetic tail: The growth phase. *Planetary Space Science*, *20*, 1521-1539. doi: 10.1016/0032-0633(72)90054-2

Meng, X., Mannucci, A. J., Verkhoglyadova, O. P., & Tsurutani, B. T. (2016, April). On forecasting ionospheric total electron content responses to high-speed solar wind streams. *Journal of Space Weather and Space Climate*, *6*, A19. doi: 10.1051/swsc/2016014

Meng, X., Mannucci, A. J., Verkhoglyadova, O. P., Tsurutani, B. T., Ridley, A. J., & Shim, J.-S. (2020, February). Thermosphere-Ionosphere Modeling With Forecastable Inputs: Case Study of the June 2012 High-Speed Stream Geomagnetic Storm. *Space Weather*, *18*(2), e02352. doi: 10.1029/2019SW002352

Meng, X., Ozturk, D. S., Verkhoglyadova, O. P., Varney, R. H., Reimer, A. S., Semeter, J. L., ... Zhan, W. (2022, December). Energy Deposition by Mesoscale High-Latitude Electric Fields Into the Thermosphere During the 26 October 2019 Geomagnetic Storm. *Journal of Geophysical Research (Space Physics)*, *127*(12), e2022JA030716. doi: 10.1029/2022JA030716

Meredith, N. P., Bortnik, J., Horne, R. B., Li, W., & Shen, X.-C. (2021). Statistical investigation of the frequency dependence of the chorus source mechanism of plasmaspheric hiss. *Geophysical Research Letters*, *48*(6), e2021GL092725. Retrieved from `https://agupubs.onlinelibrary.wiley.com/doi/abs/10.1029/2021GL092725` (e2021GL092725 2021GL092725) doi: https://doi.org/10.1029/2021GL092725

Miyoshi, Y., Katoh, Y., Nishiyama, T., Sakanoi, T., Asamura, K., & Hirahara, M. (2010, October). Time of flight analysis of pulsating aurora electrons, considering wave-particle interactions with propagating whistler mode waves. *J.*

Geophys. Res., *115*, A10312. doi: 10.1029/2009JA015127

Moen, J., Oksavik, K., Alfonsi, L., Daabakk, Y., Romano, V., & Spogli, L. (2013, January). Space weather challenges of the polar cap ionosphere. *Journal of Space Weather and Space Climate*, *3*, A02. doi: 10.1051/swsc/2013025

Morton, Y. J., Yang, Z., Breitsch, B., Bourne, H., & Rino, C. (2020). Ionospheric effects, monitoring, and mitigation techniques. In *Position, navigation, and timing technologies in the 21st century* (p. 879-937). John Wiley Sons, Ltd. doi: https://doi.org/10.1002/9781119458449.ch31

Newell, P. T., Sotirelis, T., & Wing, S. (2009, September). Diffuse, monoenergetic, and broadband aurora: The global precipitation budget. *J. Geophys. Res.*, *114*, A09207. doi: 10.1029/2009JA014326

Nishimura, Y., Bortnik, J., Li, W., Thorne, R. M., Lyons, L. R., Angelopoulos, V., … Auster, U. (2010, October). Identifying the Driver of Pulsating Aurora. *Science*, *330*, 81-84. doi: 10.1126/science.1193186

Nishimura, Y., Bortnik, J., Li, W., Thorne, R. M., Ni, B., Lyons, L. R., … Auster, U. (2013, Feb). Structures of dayside whistler-mode waves deduced from conjugate diffuse aurora. *Journal of Geophysical Research (Space Physics)*, *118*(2), 664-673. doi: 10.1029/2012JA018242

Nishimura, Y., Kelly, T., Jayachandran, P. T., Mrak, S., Semeter, J. L., Donovan, E. F., … Nishitani, N. (2023, August). Nightside High-Latitude Phase and Amplitude Scintillation During a Substorm Using 1-Second Scintillation Indices. *Journal of Geophysical Research (Space Physics)*, *128*(8), e2023JA031402. doi: 10.1029/2023JA031402

Nishimura, Y., Mrak, S., Semeter, J. L., Coster, A. J., Jayachandran, P. T., Groves, K. M., … Ruohoniemi, J. M. (2021, June). Evolution of Mid-latitude Density Irregularities and Scintillation in North America During the 7-8 September 2017 Storm. *Journal of Geophysical Research (Space Physics)*, *126*(6), e29192. doi: 10.1029/2021JA029192

Okuzawa, T., & Davies, K. (1981, March). Pulsations in total columnar electron content. *J. Geophys. Res.*, *86*(A3), 1355-1364. doi: 10.1029/JA086iA03p01355

Pi, X., Mannucci, A. J., Lindqwister, U. J., & Ho, C. M. (1997, September). Monitoring of global ionospheric irregularities using the Worldwide GPS Network.

Geophys. Res. Lett., *24*(18), 2283-2286. doi: 10.1029/97GL02273

Picone, J. M., Hedin, A. E., Drob, D. P., & Aikin, A. C. (2002, December). NRLMSISE-00 empirical model of the atmosphere: Statistical comparisons and scientific issues. *Journal of Geophysical Research (Space Physics)*, *107*(A12), 1468. doi: 10.1029/2002JA009430

Pilipenko, V., Belakhovsky, V., Murr, D., Fedorov, E., & Engebretson, M. (2014, June). Modulation of total electron content by ULF Pc5 waves. *Journal of Geophysical Research (Space Physics)*, *119*(6), 4358-4369. doi: 10.1002/2013JA019594

Prikryl, P., Jayachandran, P. T., Chadwick, R., & Kelly, T. D. (2015, May). Climatology of GPS phase scintillation at northern high latitudes for the period from 2008 to 2013. *Annales Geophysicae*, *33*(5), 531-545. doi: 10.5194/angeo-33-531-2015

Pulkkinen, A., Bernabeu, E., Thomson, A., Viljanen, A., Pirjola, R., Boteler, D., … MacAlester, M. (2017, Jul). Geomagnetically induced currents: Science, engineering, and applications readiness. *Space Weather*, *15*(7), 828-856. doi: 10.1002/2016SW001501

Ridley, A. J., Deng, Y., & Tóth, G. (2006, May). The global ionosphere thermosphere model. *Journal of Atmospheric and Solar-Terrestrial Physics*, *68*(8), 839-864. doi: 10.1016/j.jastp.2006.01.008

Rino, C. (2011). *The theory of scintillation with applications in remote sensing.* John Wiley & Sons.

Sandhu, J. K., Rae, I. J., Staples, F. A., Hartley, D. P., Walach, M. T., Elsden, T., & Murphy, K. R. (2021, July). The Roles of the Magnetopause and Plasmapause in Storm-Time ULF Wave Power Enhancements. *Journal of Geophysical Research (Space Physics)*, *126*(7), e29337. doi: 10.1029/2021JA029337

SantolíK, O., Gurnett, D. A., Pickett, J. S., Parrot, M., & Cornilleau-Wehrlin, N. (2003, July). Spatio-temporal structure of storm-time chorus. *J. Geophys. Res.*, *108*, 1278. doi: 10.1029/2002JA009791

Schunk, R. W., Scherliess, L., Sojka, J. J., Thompson, D. C., Anderson, D. N., Codrescu, M., … Howe, B. M. (2004, February). Global Assimilation of Ionospheric Measurements (GAIM). *Radio Science*, *39*(1), RS1S02. doi: 10.1029/2002RS002794

Sheng, C., Deng, Y., Zhang, S.-R., Nishimura, Y., & Lyons, L. R. (2020, February). Relative Contributions of Ion Convection and Particle Precipitation to Exciting Large-Scale Traveling Atmospheric and Ionospheric Disturbances. *Journal of Geophysical Research (Space Physics)*, *125*(2), e27342. doi: 10.1029/2019JA027342

Shi, R., Li, W., Ma, Q., Green, A., Kletzing, C. A., Kurth, W. S., ... Reeves, G. D. (2019, February). Properties of Whistler Mode Waves in Earth's Plasmasphere and Plumes. *Journal of Geophysical Research (Space Physics)*, *124*(2), 1035-1051. doi: 10.1029/2018JA026041

Shi, X., Baker, J. B. H., Ruohoniemi, J. M., Hartinger, M. D., Murphy, K. R., Rodriguez, J. V., ... Angelopoulos, V. (2018, October). Long-Lasting Poloidal ULF Waves Observed by Multiple Satellites and High-Latitude SuperDARN Radars. *Journal of Geophysical Research (Space Physics)*, *123*(10), 8422-8438. doi: 10.1029/2018JA026003

Shi, X., Zhang, X.-J., Artemyev, A., Angelopoulos, V., Hartinger, M. D., Tsai, E., & Wilkins, C. (2022, December). On the Role of ULF Waves in the Spatial and Temporal Periodicity of Energetic Electron Precipitation. *Journal of Geophysical Research (Space Physics)*, *127*(12), e2022JA030932. doi: 10.1029/2022JA030932

Skone, S. (2009, February). Using GPS TEC measurements to detect geomagnetic Pc 3 pulsations. *Radio Science*, *44*(19), RS0A27. doi: 10.1029/2008RS004106

Southwood, D., & Hughes, W. (1983). Theory of hydromagnetic waves in the magnetosphere. *Space Science Reviews*, *35*(4), 301–366.

Southwood, D. J., & Kivelson, M. G. (1981, Jul). Charged particle behavior in low-frequency geomagnetic pulsations 1. Transverse waves. *J. Geophys. Res.*, *86*(A7), 5643-5655. doi: 10.1029/JA086iA07p05643

Spicher, A., Cameron, T., Grono, E. M., Yakymenko, K. N., Buchert, S. C., Clausen, L. B. N., ... Moen, J. I. (2015, January). Observation of polar cap patches and calculation of gradient drift instability growth times: A Swarm case study. *Geophys. Res. Lett.*, *42*(2), 201-206. doi: 10.1002/2014GL062590

Spicher, A., Clausen, L. B. N., Miloch, W. J., Lofstad, V., Jin, Y., & Moen, J. I. (2017, March). Interhemispheric study of polar cap patch occurrence based on Swarm in situ data. *Journal of Geophysical Research (Space Physics)*, *122*(3),

3837-3851. doi: 10.1002/2016JA023750

Spogli, L., Alfonsi, L., de Franceschi, G., Romano, V., Aquino, M. H. O., & Dodson, A. (2009, September). Climatology of GPS ionospheric scintillations over high and mid-latitude European regions. *Annales Geophysicae*, *27*(9), 3429-3437. doi: 10.5194/angeo-27-3429-2009

Streltsov, A. V., & Lotko, W. (2008, May). Coupling between density structures, electromagnetic waves and ionospheric feedback in the auroral zone. *Journal of Geophysical Research (Space Physics)*, *113*(A5), A05212. doi: 10.1029/2007JA012594

Takahashi, K., & Anderson, B. J. (1992, July). Distribution of ULF energy (f<=80 mHz) in the inner magnetosphere: A statistical analysis of AMPTE CCE magnetic field data. *J. Geophys. Res.*, *97*(A7), 10751-10773. doi: 10.1029/92JA00328

Themens, D. R., Watson, C., Žagar, N., Vasylkevych, S., Elvidge, S., McCaffrey, A., ... Jayachandran, P. T. (2022, April). Global Propagation of Ionospheric Disturbances Associated With the 2022 Tonga Volcanic Eruption. *Geophys. Res. Lett.*, *49*(7), e98158. doi: 10.1029/2022GL09815810.1002/essoar.10510350.1

Tsurutani, B. T., & Smith, E. J. (1974, January). Postmidnight chorus: A substorm phenomenon. *J. Geophys. Res.*, *79*, 118-127. doi: 10.1029/JA079i001p00118

Tsyganenko, N. A. (1995, April). Modeling the Earth's magnetospheric magnetic field confined within a realistic magnetopause. *J. Geophys. Res.*, *100*, 5599-5612. doi: 10.1029/94JA03193

Tyler, E., Breneman, A., Cattell, C., Wygant, J., Thaller, S., & Malaspina, D. (2019, Mar). Statistical Occurrence and Distribution of High-Amplitude Whistler Mode Waves in the Outer Radiation Belt. *Geophys. Res. Lett.*, *46*(5), 2328-2336. doi: 10.1029/2019GL082292

Verkhoglyadova, O. (2024). *Dataset for the paper "Magnetospheric control of ionospheric TEC perturbations via whistler-mode and ULF waves" paper to be submitted to AGU Advances.* JPL Open Repository. Retrieved from `https://doi.org/10.48577/jpl.LGI5JS` doi: 10.48577/jpl.LGI5JS

Verkhoglyadova, O., Meng, X., Mannucci, A. J., Shim, J. S., & McGranaghan, R. (2020, September). Evaluation of Total Electron Content Prediction Using Three Ionosphere-Thermosphere Models. *Space Weather*, *18*(9),

e2020SW002452. doi: 10.1029/2020SW002452

Wang, B., Nishimura, Y., Hartinger, M., Sivadas, N., Lyons, L. L., Varney, R. H., & Angelopoulos, V. (2020, August). Ionospheric Modulation by Storm Time Pc5 ULF Pulsations and the Structure Detected by PFISR-THEMIS Conjunction. *Geophys. Res. Lett.*, *47*(16), e89060. doi: 10.1029/2020GL089060

Watson, C., Jayachandran, P. T., & MacDougall, J. W. (2016, May). Characteristics of GPS TEC variations in the polar cap ionosphere. *Journal of Geophysical Research (Space Physics)*, *121*(5), 4748-4768. doi: 10.1002/2015JA022275

Watson, C., Jayachandran, P. T., Singer, H. J., Redmon, R. J., & Danskin, D. (2015, September). Large-amplitude GPS TEC variations associated with Pc5-6 magnetic field variations observed on the ground and at geosynchronous orbit. *Journal of Geophysical Research (Space Physics)*, *120*(9), 7798-7821. doi: 10.1002/2015JA021517

Watson, C., Jayachandran, P. T., Singer, H. J., Redmon, R. J., & Danskin, D. (2016, February). GPS TEC response to Pc4 "giant pulsations". *Journal of Geophysical Research (Space Physics)*, *121*(2), 1722-1735. doi: 10.1002/2015JA022253

Watt, C. E. J., Degeling, A. W., Rankin, R., Murphy, K. R., Rae, I. J., & Singer, H. J. (2011, October). Ultralow-frequency modulation of whistler-mode wave growth. *Journal of Geophysical Research (Space Physics)*, *116*(A10), A10209. doi: 10.1029/2011JA016730

Xia, Z., Chen, L., Dai, L., Claudepierre, S. G., Chan, A. A., Soto-Chavez, A. R., & Reeves, G. D. (2016, September). Modulation of chorus intensity by ULF waves deep in the inner magnetosphere. *Geophys. Res. Lett.*, *43*, 9444-9452. doi: 10.1002/2016GL070280

Xia, Z., Chen, L., & Li, W. (2020, November). Statistical Study of Chorus Modulations by Background Magnetic Field and Plasma Density. *Geophys. Res. Lett.*, *47*(22), e89344. doi: 10.1029/2020GL089344

Xiong, C., Park, J., Lühr, H., Stolle, C., & Ma, S. Y. (2010, September). Comparing plasma bubble occurrence rates at CHAMP and GRACE altitudes during high and low solar activity. *Annales Geophysicae*, *28*(9), 1647-1658. doi: 10.5194/angeo-28-1647-2010

Yeh, K. C., & Liu, C. H. (1982, April). Radio wave scintillations in the ionosphere.

IEEE Proceedings, *70*, 324-360.

Yeoman, T. K., James, M., Mager, P. N., & Klimushkin, D. Y. (2012, June). SuperDARN observations of high-m ULF waves with curved phase fronts and their interpretation in terms of transverse resonator theory. *Journal of Geophysical Research (Space Physics)*, *117*(A6), A06231. doi: 10.1029/2012JA017668

Zettergren, M. D., & Snively, J. B. (2015, September). Ionospheric response to infrasonic-acoustic waves generated by natural hazard events. *Journal of Geophysical Research (Space Physics)*, *120*(9), 8002-8024. doi: 10.1002/2015JA021116

Zhai, C., Shi, X., Wang, W., Hartinger, M. D., Yao, Y., Peng, W., ... Baker, J. B. H. (2021, July). Characterization of High-m ULF Wave Signatures in GPS TEC Data. *Geophys. Res. Lett.*, *48*(14), e94282. doi: 10.1029/2021GL094282

Zhang, S.-R., Vierinen, J., Aa, E., Goncharenko, L. P., Erickson, P. J., Rideout, W., ... Spicher, A. (2022, March). 2022 Tonga Volcanic Eruption Induced Global Propagation of Ionospheric Disturbances via Lamb Waves. *Frontiers in Astronomy and Space Sciences*, *9*, 871275. doi: 10.3389/fspas.2022.871275

Zhang, X. J., Angelopoulos, V., Artemyev, A. V., Hartinger, M. D., & Bortnik, J. (2020, October). Modulation of Whistler Waves by Ultra-Low-Frequency Perturbations: The Importance of Magnetopause Location. *Journal of Geophysical Research (Space Physics)*, *125*(10), e28334. doi: 10.1029/2020JA028334

Zhang, X.-J., Chen, L., Artemyev, A. V., Angelopoulos, V., & Liu, X. (2019, November). Periodic Excitation of Chorus and ECH Waves Modulated by Ultralow Frequency Compressions. *Journal of Geophysical Research (Space Physics)*, *124*(11), 8535-8550. doi: 10.1029/2019JA027201

Zong, Q., Rankin, R., & Zhou, X. (2017, Dec). The interaction of ultra-low-frequency pc3-5 waves with charged particles in Earth's magnetosphere. *Reviews of Modern Plasma Physics*, *1*(1), 10. doi: 10.1007/s41614-017-0011-4

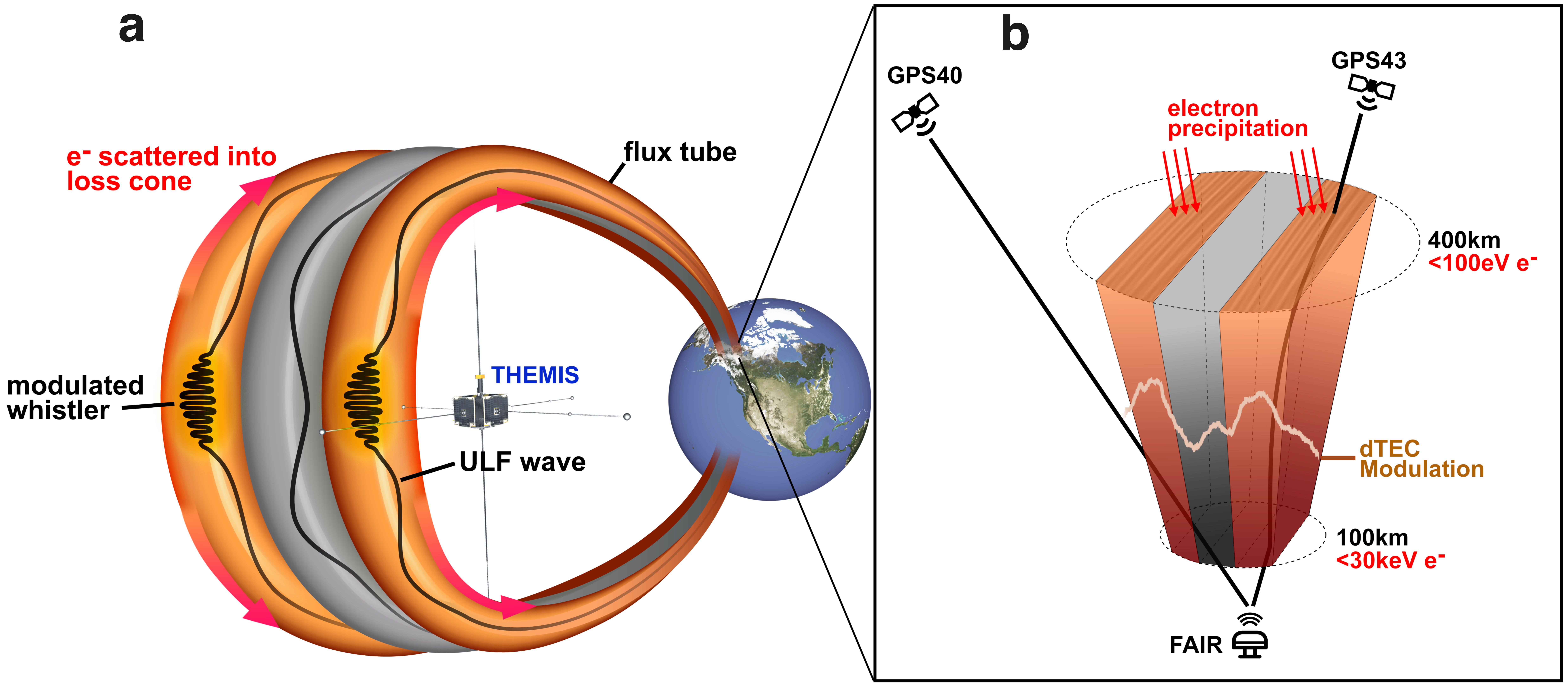

a
e- scattered into
loss cone
flux tube
modulated
whistler
THEMIS
ULF wave
b
GPS40
GPS43
electron
precipitation
400km
<100eV e-
dTEC
Modulation
100km
<30keV e-
FAIR

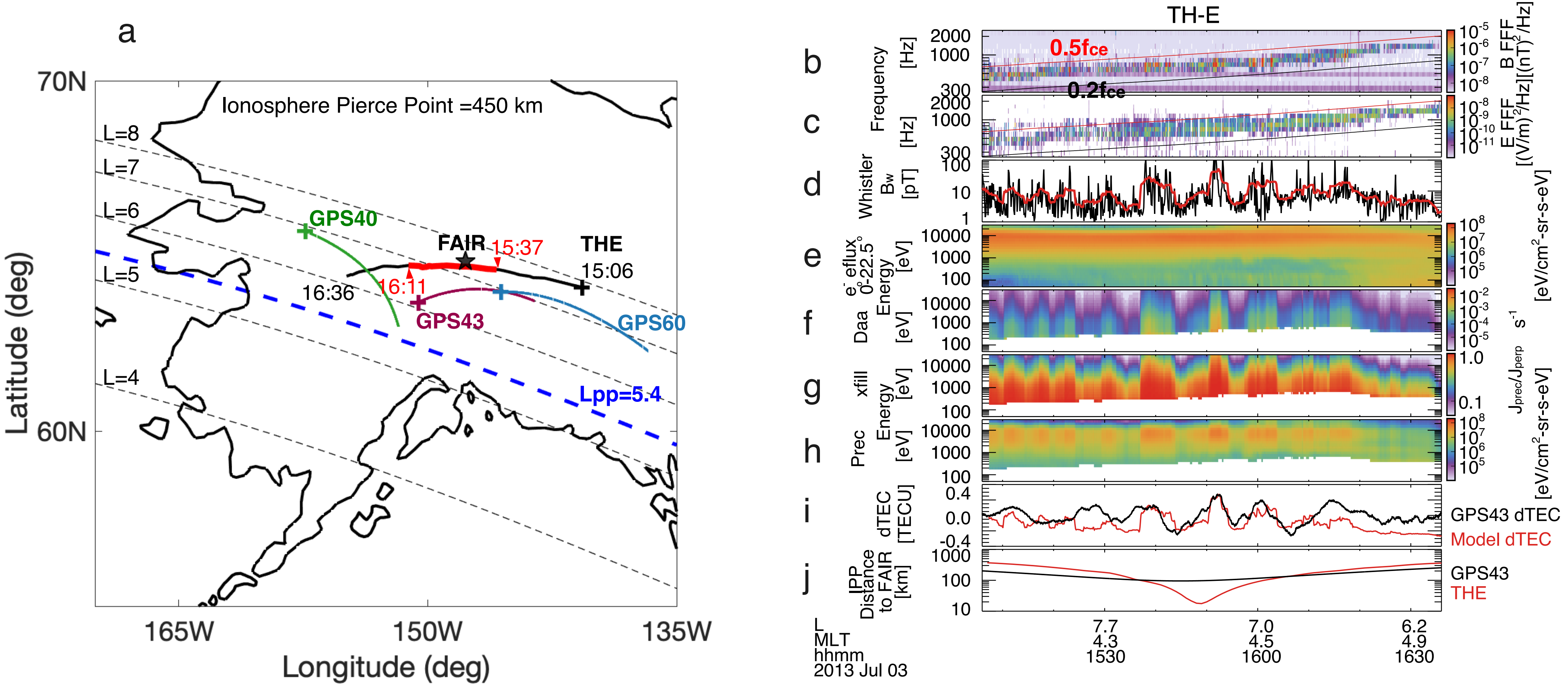
a
Ionosphere Pierce Point =450 km
L=8
L=7
L=6
L=5
L=4
GPS40
FAIR
15:37
THE
15:06
16:11
16:36
GPS43
GPS60
Lpp=5.4
70N
60N
165W
150W
135W
Latitude (deg)
Longitude (deg)
TH-E
b
c
d
e
f
g
h
i
j
0.5fce
0.2fce
Frequency [Hz]
B FFF [(nT)²/Hz]
E FFF [(V/m)²/Hz]
Whistler Bw [pT]
e⁻ eflux 0°-22.5° Energy [eV]
[eV/cm²-sr-s-eV]
Daa Energy [eV]
s⁻¹
xfill Energy [eV]
Jprec/Jperp
Prec Energy [eV]
dTEC [TECU]
GPS43 dTEC
Model dTEC
IPP Distance to FAIR [km]
GPS43
THE
L
MLT
hhmm
2013 Jul 03
7.7
4.3
1530
7.0
4.5
1600
6.2
4.9
1630

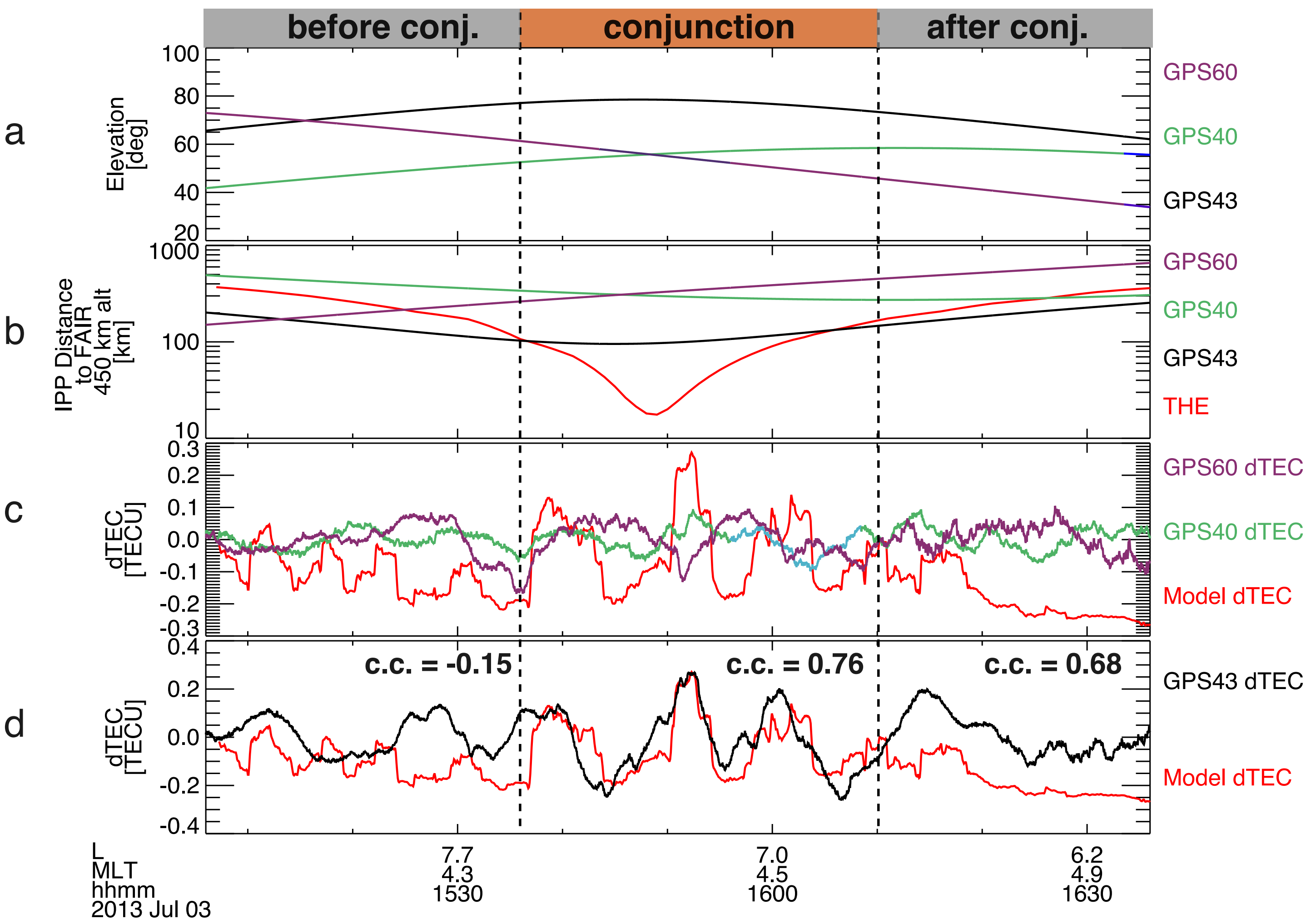

before conj.
conjunction
after conj.
a
Elevation [deg]
100
80
60
40
20
GPS60
GPS40
GPS43
b
IPP Distance to FAIR 450 km alt [km]
1000
100
10
GPS60
GPS40
GPS43
THE
c
dTEC [TECU]
0.3
0.2
0.1
0.0
-0.1
-0.2
-0.3
GPS60 dTEC
GPS40 dTEC
Model dTEC
d
dTEC [TECU]
0.4
0.2
0.0
-0.2
-0.4
c.c. = -0.15
c.c. = 0.76
c.c. = 0.68
GPS43 dTEC
Model dTEC
L
MLT
hhmm
2013 Jul 03
7.7
4.3
1530
7.0
4.5
1600
6.2
4.9
1630

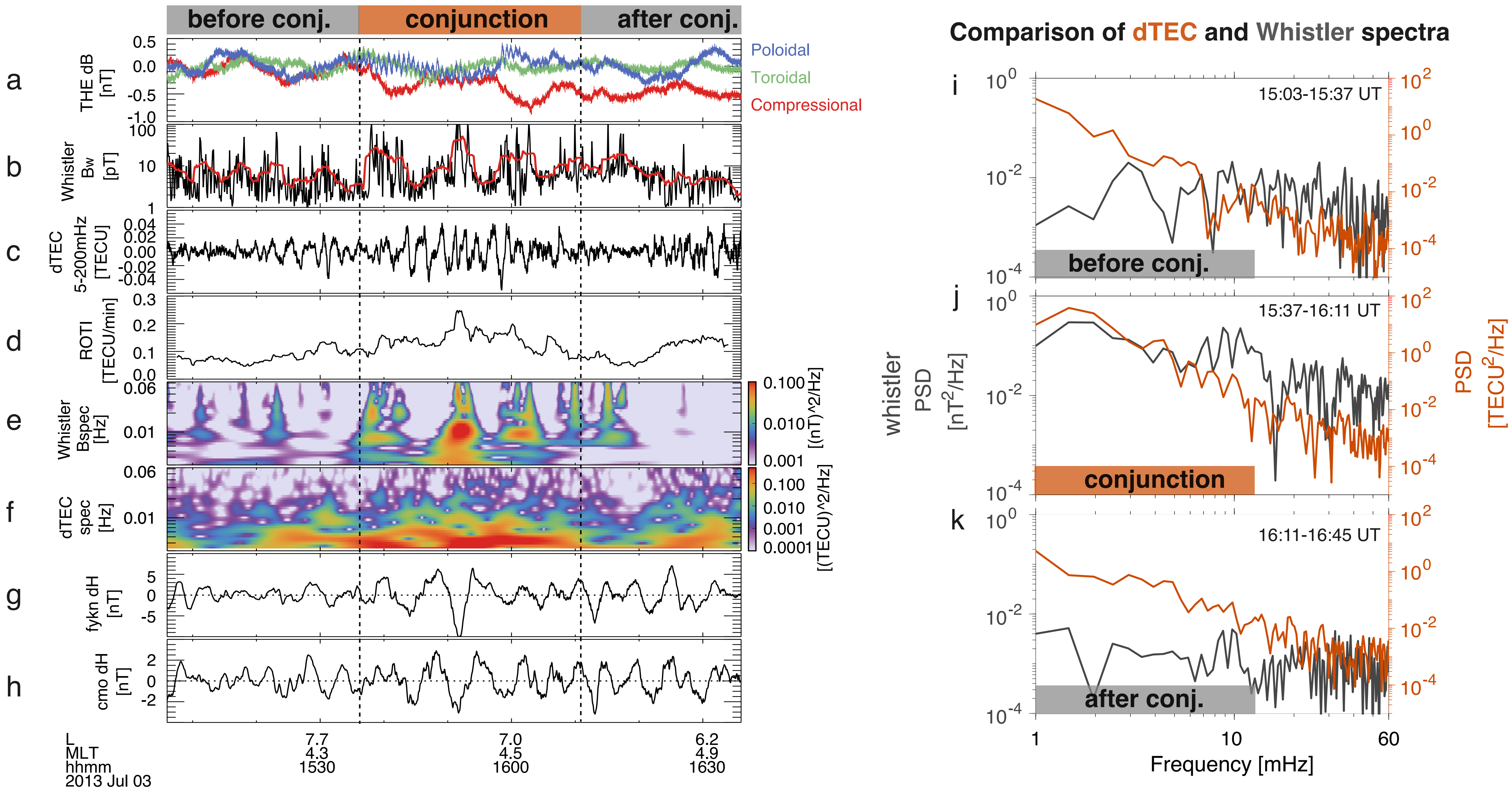

before conj.
conjunction
after conj.
Poloidal
Toroidal
Compressional
THE dB [nT]
Whistler Bw [pT]
dTEC 5-200mHz [TECU]
ROTI [TECU/min]
Whistler Bspec [Hz]
[(nT)^2/Hz]
dTEC spec [Hz]
[(TECU)^2/Hz]
fykn dH [nT]
cmo dH [nT]
L
MLT
hhmm
2013 Jul 03
7.7
4.3
1530
7.0
4.5
1600
6.2
4.9
1630
Comparison of dTEC and Whistler spectra
15:03-15:37 UT
15:37-16:11 UT
16:11-16:45 UT
whistler PSD [nT^2/Hz]
PSD [TECU^2/Hz]
Frequency [mHz]

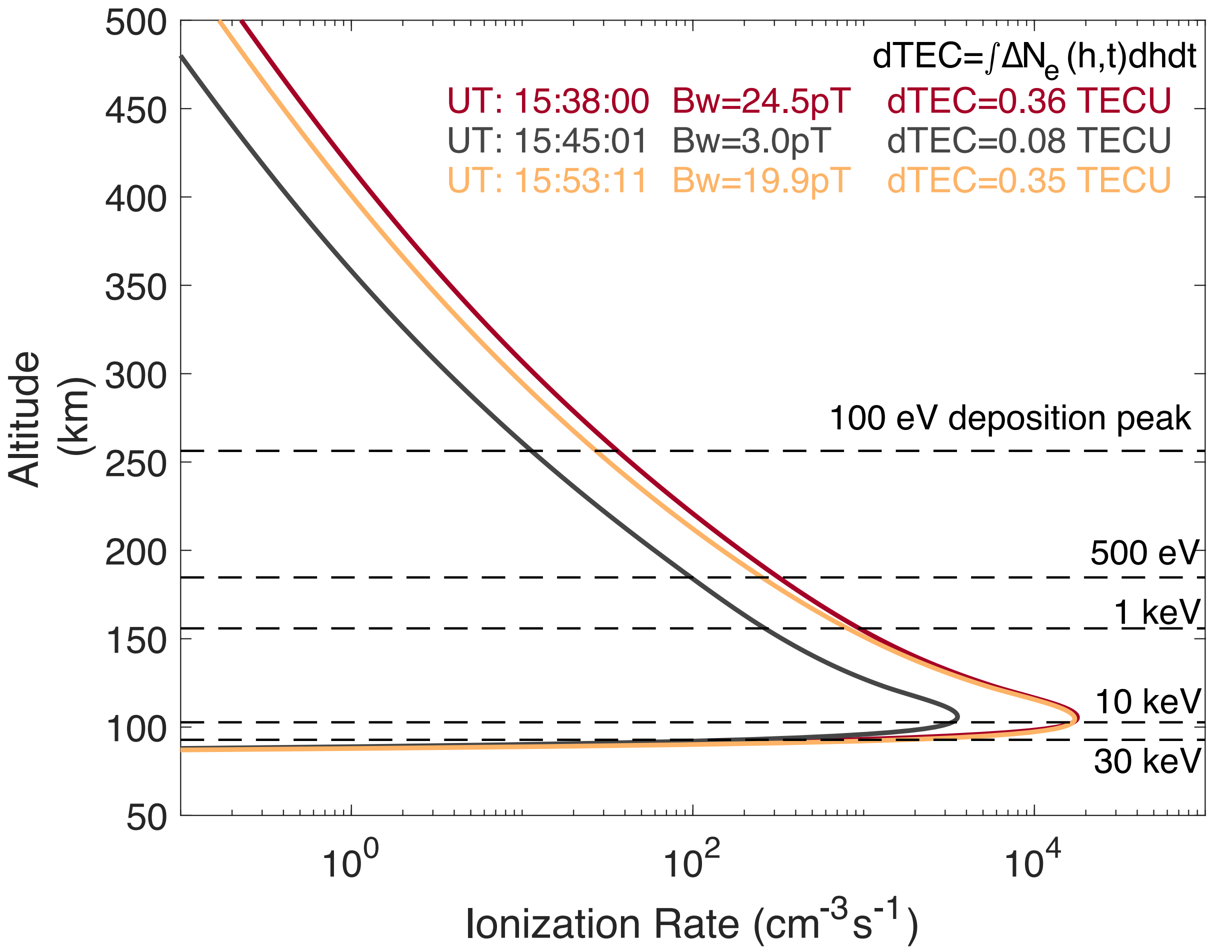

dTEC=∫ΔNe (h,t)dhdt
UT: 15:38:00 Bw=24.5pT dTEC=0.36 TECU
UT: 15:45:01 Bw=3.0pT dTEC=0.08 TECU
UT: 15:53:11 Bw=19.9pT dTEC=0.35 TECU
100 eV deposition peak
500 eV
1 keV
10 keV
30 keV
Altitude (km)
Ionization Rate (cm-3s-1)
500
450
400
350
300
250
200
150
100
50
10^0
10^2
10^4
