# Supplementary material for: Magnetospheric Control of Ionospheric TEC Perturbations via Whistler‐Mode and ULF Waves
Source: AGU Adv. 2024 Nov 25;5(6):e2024AV001302. doi: 10.1029/2024AV001302 (PMC11600791; doi:10.1029/2024AV001302)
Supplement: Supplementary file 4 — Author Response to Peer Review Comments [file AGA2-5-0-s006.pdf]

<u>Response to Reviewer/Editor Comments</u>

A reviewer noted the following which should be addressed along with the formal review recommendations:

Dear Editor,

While I cannot be 100% certain, I believe that some parts of this manuscript were written using AI. I feel that this can be seen more strongly for the first part of the manuscript, perhaps particularly for what is written before the "Results" section.

I based this on the following points:
- the hyphenation of terms that are very basic and would not be hyphenated by someone in this field (very-low-frequency, ultra-low-frequency)
- the use of strange sentence structures in some cases (footprints of THEMIS-E are field-line traced)
- the use of overly meaningless but descriptive words or expressions ("physics-based", fortuitously, well aligned in time and space, this optimally timed and positioned space-ground conjunction offers a unique opportunity... etc)
- the constant re-writing of acronyms definitions throughout the text

I am not sure what the guidelines for AGU Advances are, and I might be wrong, but I just wanted to bring this to your attention.

--

Authors who use AI tools in the writing of a manuscript, production of images or graphical elements of the paper, or in the collection and analysis of data, must be transparent by disclosing details of use, including which AI tool was used and how it was used, in the Materials and Methods (or similar section) of the paper. Authors are fully responsible for the content of their manuscript.

Response:

For specific comments, please refer to our detailed responses to reviewer. The first author of the paper has used ChatGPT for text editing of some sentences in the Introduction, and some part of the Results, for example, when discussing conjunction geometry (e.g., optimally timed and positioned...). Some of these sentences have been modified after reviewer's comments.

We have declared the use of ChatGPT for text editing in the section of "Materials and Methods" following "Open Research".

Response to Reviewers

Reviewer #1 (Comments to Author (shown to authors):

The authors conducted an event analysis of ULF-modulated whistler-mode waves in the magnetosphere, which drive electron precipitation, leading to ionization and Total Electron Content (TEC) changes. The in-situ satellite data and wave analysis presented in the manuscript are well executed and described. However, there is a lack of other supporting ionospheric observations, and potential competing processes in the ionosphere are not discussed.

Response:

We appreciate the reviewer's efforts in evaluating our manuscript and providing helpful comments. Below we address the comments the reviewer has brought up. Please note that the original review comments are presented in black and our responses are shown in blue.

The paper lacks supporting evidence from nearby ionosondes or radar, which could provide information about the ionospheric E and F region conditions during this event. Such information is crucial for selecting the appropriate Global Navigation Satellite System (GNSS) Ionospheric Pierce Point (IPP) height. Based on the ionization profile calculation in the paper, an IPP at 150 km or even lower seems more appropriate. This adjustment would reduce the scale of the satellite footpaths near FAIR but is more logical. If there is a clear F layer present during this event, the hmF2 information would be helpful in determining the IPP choice. The 450 km IPP selection is higher than the typical 350 km used in the Madrigal TEC database.

Response:

We thank the reviewer for the great suggestion. We have checked the nearby ionosonde measurements and found that the data from the Eielson station (64.66ºN, 212.03ºE, https://giro.uml.edu/ionoweb/) were available every 15 min during our event. The Poker Flat incoherent scatter radar was not collecting data during our event. Now we have used the measured hmF2 height of ~300 km from the ionogram shown below as the IPP altitude in the manuscript.

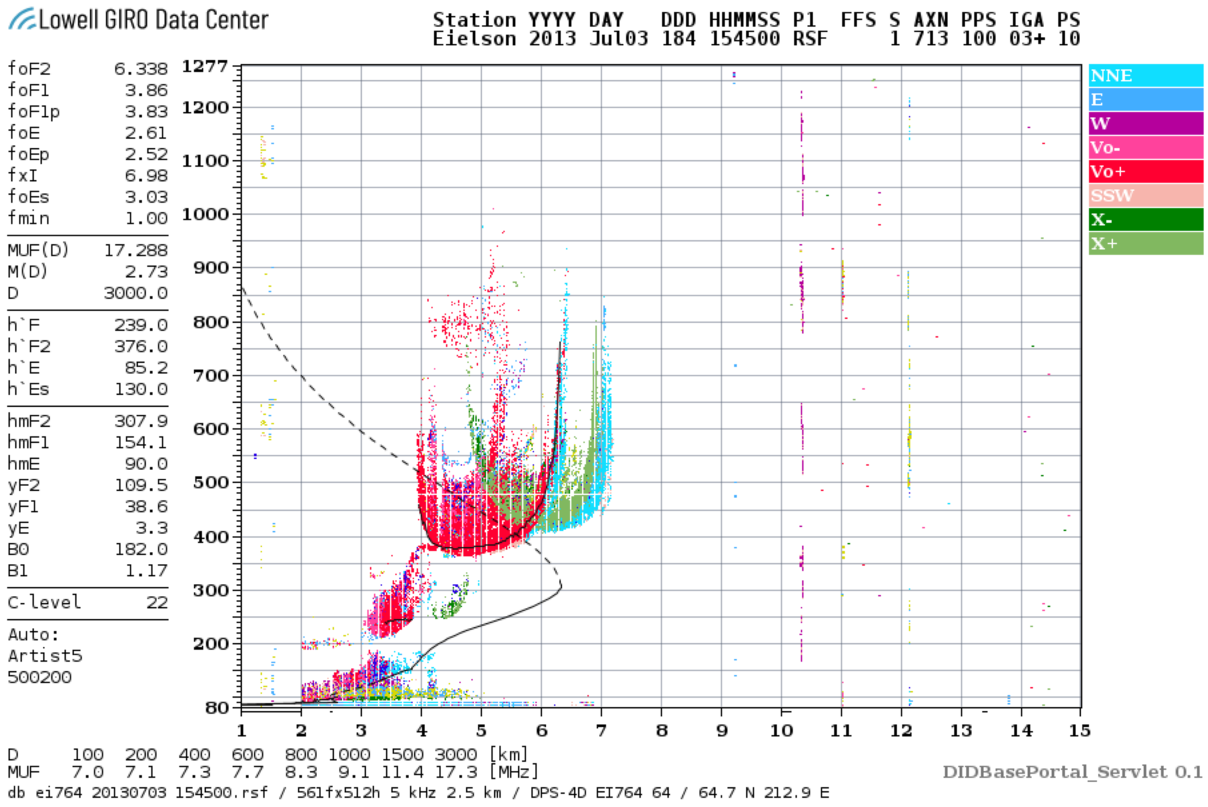

We have included the ionogram figure in Supporting Information. Based on the new IPP altitude of ~300 km, we have also recalculated the amplitude and spatial scales of the measured dTEC. We have also changed projected footprints and distances in Figure 2 and Figure 3.

To estimate dTEC spatial scales, we do not take any assumptions about spatial variations as we previously did. Instead, we have estimated the plasma flow velocity from the average Doppler shift factor of ~1.15 (measured in Figure 4j) and the pierce point velocity. This enables us to estimate the scale sizes based on $ds = \left(|\vec{v}_{struct}| - |\vec{v}_{ipp}|\right) \cdot dt$. Please refer to the revision-highlighted manuscript for these changes. The updated scale sizes of 5--100 km do not differ much from the previously estimated values.

We also agree that a lower IPP altitude at ~150 km could be more reasonable for the altitudes of dTEC, compared with 300-km used for the background TEC. That is, modulations of dTEC do not necessarily occur at the same altitudes as the altitude of the background density peak. In the revised manuscript, we have decided to keep the projection figure and scale estimates using 150km IPP in Supporting Information as we have done before.

In fact, the change of the IPP altitude from 450 km to 300 km has little effect on the estimated vertical dTEC, because of the high elevation angles (>60º) in our case (Rama Rao et al., 2006, doi: 10.5194/angeo-24-2159-2006). Thus, this IPP change does not affect our main conclusions about whistler-driven electron precipitation and subsequent generation of ionospheric dTEC. These conclusions are based on quantitative physical modeling of dTEC as well as on spectra and time-series correlation analyses, independent of our choice of IPP altitudes.

If there is an ionospheric F layer present during this event, the transport process of F-layer plasma should be considered and discussed to determine whether the TEC modulation is due to the transport process.

Response:

As the reviewer has pointed out, and verified by the ionosonde measurements, there exists an ionosphere F2 layer during this event at ~300 km. This F2 layer were there for a long time, varying from 309.7 km at 15:30 UT to 307.9 km at 15:45 UT and 281.1 km at 16:00 UT. We have now included discussions on the effects of the potential F-layer plasma transport on dTEC modulation in the event. It goes as follows:

"*The vertical component of the $\vec{E} \times \vec{B}$ drift associated with ULF waves can induce vertical bulk motion of ionospheric plasma with a drift velocity $V_z = E_y \cos I / B_0$, where I is the local magnetic inclination. This vertical transport can alter the altitude-dependent recombination rate, thereby contributing to electron density or TEC modulations (Poole and Sutcliffe, 1987, doi: 10.1016/0021-9169(87)90058-4; Pilipenko et al., 2014, doi: 10.1002/2013JA019594). These effects are potentially important in midlatitude and equatorial regions (Yizengaw et al., 2018, doi: 10.1029/2018GL078163; Zou et el. 2017, doi: 10.1002/2016JA023697) but are expected to be less significant at high latitudes where the magnetic inclination is large. In our case, the magnetic inclination angle is such that cosI~0.2, and the magnetic perturbations are only a few nT, resulting in electric field perturbations Ey<1 mV/m (Yizengaw et al., 2018). Based on similar estimations from Pilipenko et al., (2014), the resulting changes in dNe/Ne or dTEC/TEC are only 0.04%, corresponding to dTEC of < 0.01 TECU given a background TEC of ~20 TECU. This level of dTEC is insignificant compared with the observed 0.5 TECU. Moreover, the timescales of TEC changes due to recombination rate changes associated with vertical plasma motion are typically longer than 1 hour (Yizengaw et al., 2006, doi: 10.1029/2005JA011433; Maruyama et al., 2004, doi: 10.1029/2004JA010451; Heelis et al., 2009, doi: 10.1029/2008JA013690], which are much larger than the ULF modulation timescales of several minutes observed in our case. Therefore, the observed ULF-modulated high-latitude dTEC are unlikely to be explained by vertical plasma transport and recombination rate changes in the F region.*"

On the other hand, our paper combines conjugate observations and physical modeling of dTEC, strongly supporting the scenario of precipitation induced dTEC due to modulation of ULF and whistler-mode

waves.

The conclusion that these phenomena are "..critically contributing to TEC perturbations at high latitudes. As such, incorporating these magnetospheric phenomena is vital for improving the accuracy of ionospheric TEC models." may be overstated, given that the TEC modulation shown in this event is only ~0.5 TECU, which is smaller than the typical TEC data uncertainty of ~1-2 TECU.

Response:

The sentence has been changed to "*critically contributing to dTEC at high latitudes. As such, incorporating these magnetospheric phenomena is important for improving the accuracy of ionospheric dTEC models.*"

The level of dTEC~0.5 TECU is very significant and representative in the nightside auroral region. We assume the reviewer was referring to the uncertainty of absolute TEC measurements reaching ~1-2 TECU. For our study, we use relative TEC perturbations that typically have much better accuracy derived from differential GPS phase measurements (e.g., <0.02 TECU, Coster et al., 2013, doi: 10.1002/rds.20011). Uncertainties in the absolute TEC baseline have little influence on the relative TEC perturbation measurements. Using these TEC perturbations, one can resolve the features of travelling ionospheric disturbances (TIDs), which are often reported in the literature to be <0.2 TECU (e.g., Zhang et al., 2022, doi: 10.3389/fspas.2022.871275).

Both the scale size and amplitude of TEC perturbations are important to studies of scintillations (e.g., Keskinen&Ossakow, 1983, doi: 10.1029/JA088iA01p00474; Kelley, 2009, The Earth's ionosphere: Plasma physics and electrodynamics). Previous studies show that the spectra of irregularities that cause scintillations have a power-law spectrum. dTEC or density fluctuations at smaller scales naturally have smaller amplitudes. As scintillations have been suggested to be produced by turbulence cascading processes, these meso-scale structures can be directly related to formation of smaller-scale scintillations, compared with larger-scale, larger-amplitude TEC structures (e.g., Tsunoda, 1988, doi:10.1029/RG026i004p00719). Thus, these km-scale TEC perturbations provided by auroral precipitation can provide the needed density gradients that directly drive scintillations. We are currently conducting a follow-up study on this topic; our initial results show that these whistler-induced precipitation and associated meso-scale density gradients can facilitate formation of scintillation-inducing irregularities. In this sense, we argue that incorporating these magnetospheric phenomena is indeed important for improving the accuracy of ionospheric TEC perturbation models.

Reviewer #2 (Comments to Author (shown to authors):

This is a review of "Magnetospheric control of ionospheric TEC perturbations via whistler-mode and ULF waves" by Shen et al.

General comments

Overall, the methods and data are relatively well described and the correlation between TEC changes, ULF waves, and whistler-mode waves is adequately presented. However, the manuscript is sometimes really hard to follow. While I think this study is sound, my main issue is the lack of clarity of some parts of the text and how the data/conclusions are presented to the reader.

Response:

We appreciate the reviewer's efforts in evaluating our manuscript and providing helpful comments. Below we address the comments the reviewer has brought up. Please note that the original review comments are presented in black and our responses are shown in blue.

There are too many acronyms one has to keep track of, I suggest reducing their number and focusing on those that are the most important. In addition, in the first half of the manuscript, the authors use many superfluous qualifiers that should be removed or given more specificity. For example, is it that important to emphasize the observations as "fortuitous"? Aren't a majority of events generally fortuitous unless you are running a specific campaign? What does this bring to the paper? If the authors want to say that these are rare events then they should mention it properly.

Response:

We have reduced the acronyms in the manuscript, especially for those not used in the following sections. We have also reduced or clarify the qualifiers in writing of the introduction. Please see our responses in the detailed comments below.

Throughout the text, very low frequency waves should not be hyphenated, same thing for ultra low frequency waves. I also think THEMIS E does not require hyphens but that might be just personal preference.

Response:

Changed accordingly.

Related to the above as well, the ULF definition is given too many times: in the abstract, plain language abstract, introduction, and multiple times later on in the text. Please try to reduce this. Maybe just abstract and introduction? Same for TEC, dTEC, GPS etc. Maybe describing these acronyms once (or twice) should be enough.

Response:

We have reduced the acronyms usage in the manuscript.

Several links provided are not working. See Open Research comments below.

Response:

Please refer to our response below in detailed comments.

Detailed comments

** Introduction

48. How is fortuitous relevant here?

Response:

"fortuitous" has been removed throughout the manuscript. One of the important findings in the paper is that it requires very precise spatial and temporal alignment between GPS, THEMIS, and ground-based receiver to detect the phase correlation between whistler-driven and observed dTEC. The event of the manuscript was found after a preliminary survey of several months of conjunction events between the THEMIS spacecraft and GPS FAIR receiver. Therefore, the conjunction is relatively rare yet essential for probing the magnetospheric driver of ionospheric dTEC.

55. What does "physics-based" mean here?

Response:

We have used "physics-based" here to distinguish our work from other empirical and climatological models of ionosphere TEC. Physics-based modeling means we provide a description of the physical processes leading to dTEC generation, based on combined observations and calculations from fundamental equations and theory, rather than from empirical/observational statistics and probability predictions.

We have added the following sentence at the beginning of the third paragraph to introduce the different TEC modeling approaches:
"*While empirical and climatological TEC models exist (e.g., Ridout&Coster, 2006, doi: 10.1007/s10291-006-0029-5; Jakowski et al., 2011, doi:10.1029/2010RS004620), physics-based modeling of TEC perturbations remains challenging. One of the main challenges in physical modeling of dTEC and space weather prediction*..."

82. If you will not use GPS receivers again, I would recommend not using the acronym. We have too many in the text. Same for TIDs if this is not going to be a big part of the manuscript.

Response:

We used GPS in the figures and text throughout the manuscript, so we decide to keep the acronym of GPS. We delete the acronym of TIDs in the revised manuscript.

110. Since you're using equations later, it might be best to use proper vectors and not just bold for E x B drift.

Response:

Changed accordingly.

133. What does density inversion mean?

Response:

The sentence has been changed to: "*Pc5-modulated density variations observed from radar data were used to infer modulated precipitating electrons over an energy range of ~1--500 keV and an altitude of ~80--200 km.* "

140. What postulation are you referring to?

Response:

The postulation is referring to the sentence just before: "*The authors postulated that the precipitation and density perturbations are likely due to electron pitch-angle scattered into the loss cone by ULF-modulated very low frequency whistler-mode waves*."

We have changed the sentence to "*This postulation of whistler-driven dTEC is*..."

164. Again, why is fortuitously used here? What do you want to say about the event?

Response:

Please refer to the response about "fortuitous" above. We deleted fortuitously.

** Figure 1. While I understand this is a representative diagram, I believe that putting your return field line from the poles to the equators might be misleading. Would it be possible to make it more accurate? Otherwise, this figure is quite nice.

Response:

Changed accordingly.

** Data and Methodology
Please reduce the use of useless acronyms. If we are not going to see GIM, PRN, or DFB later in the text there is no need for the acronym. We have already so many acronyms to keep track of, adding more just makes the text overly cumbersome. If necessary it can be limited to the figure captions.

Response:

Acronym deleted accordingly.

Even IPP while it comes back in the text might not be necessary, you can just reduce to "pierce points" later in the text should you want a shorter version.

Response:

The acronym of "IPP" has been deleted and changed to pierce points as suggested.

178. processed at the Jet Propulsion ...

Response:

Changed accordingly.

183. Is there a particular reason why you consider 450 km alt within 300 km of FAIR and 150 km alt within 100 km? Could you please explain your choices here or refer to papers should this be related to previous selection choices/studies?

Response:

We consider 450 km altitude within 300 km in proximity to FAIR to ensure high elevation angles and better positions to observe vertical dTEC. The altitude of 450 km was initially chosen as the default pierce point altitude for JPL-processed TEC measurements. However, we have updated the pierce point altitude to 300 km, within distances of 200 km, based on ground-based ionosonde measurements and in response to the other reviewer's suggestion. While the background density peaks around 300 km in the F2 region, the modulation of dTEC likely occurs at lower altitudes. Therefore, based on the ionization profile, we also provide the results using pierce points of 150 km. At these lower altitudes, the horizontal scales change accordingly, and a distance of 100 km is considered close when projected to 150 km altitude.

We have made some modifications in this paragraph to explain our choice of pierce points based on ionosonde measurements, and that the corresponding GPS satellites are chosen because of their

closeness to the FAIR receiver and thus high elevation angles, which are better suited to resolve vertical dTEC features.

187. Why not consider elevation angles less than 30 degrees, why are these choices made? Is it a limitation of the instrument or a limitation of the data?

Response:

We have changed the sentence to: "*Measurements with elevation angles less than 30º are excluded to reduce multipath effects (e.g., Jakowski et al., 1996, doi: 10.1007/s00585-996-1429-0)*."

189. Why are you focusing on wave periods smaller than 25 min?

Response:

The sentence has been modified to: "*The low pass filter has a cutoff period of 25 min, to focus on ULF-related perturbations and reduce contributions from medium- and large-scale travelling ionosphere disturbances (Hunsucker, 1982, doi: 10.1029/RG020i002p00293)*."

229. please remove neglect

Response:

Done.

** Results
237 - 239. This sentence is superfluous. Please be more precise if you want to emphasize why this is important and unique.

Response:

The sentence has changed to "*The space-ground observations have a close spatial and temporal alignment, allowing us to link between magnetospheric and ionospheric processes along the field line*."

242. IPPs have already been defined multiple times. I like to re-instate that this might work better without an acronym as we have difficulties following the sentences.

Response:

The acronym of IPP has been deleted and THEMIS-E has been changed to THEMIS E.

244. Field-line traced? Please rewrite this sentence.

Response:

We have changed the sentence to: "*The position of THEMIS E is mapped along the field line to the ionosphere using the Tsyganenko T96 model (Tsyganenko, 1995, doi:10.1029/94JA03193) but the GPS satellites are mapped using line of sight*."

259 - 265. Please reword this paragraph to make it clearer. As it stands it is difficult to understand. Maybe break it into multiple sentences?

Response:

The paragraph has been changed to:

"*We use smoothed or averaged Bw to estimate electron precipitation. Although direct waveform data for resolving whistler-mode wave normals were absent, we can infer wave normals based on the measured whistler spectra properties of E/cB<<1 (see Supporting Information) as well as from previous*

statistical whistler observations in the nightside equatorial plasma sheet (Li et al., 2011, doi: 10.1029/2011JA017035; Agapitov et al., 2013, doi: 10.1002/jgra.50312; Meredith et al., 2021, doi: 10.1029/2021GL092725). The whistlers propagate quasi-parallel to the magnetic field, with an assumed Gaussian wave normal width of $\Delta\theta \sim 30º$ and a latitudinal distribution within $\pm 30º$. "

279. Is 0.5 TECU significant? Why?

Response:

We have provided a similar response to a comment from the other reviewer (see above). We have visually inspected the statistical distribution (~400 events) of dTEC from FAIR associated with ULF waves, and found that the 0.5 TECU perturbations can be considered typical, medium-level disturbance in the nightside auroral region. Therefore, it is representative, albeit not extreme. A separate paper presenting the statistical analyses of dTEC associated with ULF waves is currently in preparation.

We have changed the sentence to: "*Observed peak-to-peak amplitudes of dTEC reached ~0.5 TECU, which is typical, though not extreme, for the nightside auroral region*."

In fact, this level of dTEC is larger than most travelling ionospheric disturbances (~0.2 TECU) reported in the literature (e.g., Zhang et al., 2022, doi: 10.3389/fspas.2022.871275), and is comparable with those dTEC associated with the large Tohoku earthquake event (e.g., Themens et al., 2022, doi: 10.1029/2022GL098158).

Another factor that determines the significance of dTEC is its spatial scales. As mentioned in the response to the other reviewer, the medium-scale/km-scale dTEC that we study here will provide needed density gradients for formation of scintillations.

303. Do we really need the MFA acronym if it's only mentioned once?

Response:

Deleted as suggested.

319. "These observations" refer to the results of this paper or the previously mentioned discrepancy or references. Maybe be more specific.

Response:

Changed to "*Our observations imply that*..."

** Figure 2
While it may be possible that this is due to the PDF format, this figure is too small and cramped. We can barely read and understand the panels. This showcases the most important result of this paper but we can barely make sense of the description. It's hard to understand what corresponds to the labels with energies and fluxes, etc. Please find a way for the information to be clearer and easy to identify.

Response:

We have modified Figure 2 to adjust the pierce point altitude change to 300 km and have also made the figure larger. The figure size will be finalized in AGU publication.

** Figure 4
This figure is also too cramped and crowded on the panels. Any chance you can make it bigger or simplify the panel description (or at least make it clear).

Response:

The figure has been made larger and labels simplified.

** Discussion
378. Please rewrite this sentence. I'm not entirely sure what you mean here.

Response:

We have deleted the part of small magnetic perturbations in this sentence. However, we have added another paragraph detailing about the vertical plasma transport effect and about how much density or TEC perturbations are expected from the observed ground-based magnetic perturbations. Please refer to the revision-highlighted manuscript for these changes.

** Conclusions
Please remove the acronym definitions we've already seen them multiple times in previous sections.

Response:

Done.

** Open Research
All THEMIS links are not accessible or do not exist.
The DOI link for Verkhoglyadova 2024 does not exist.

Response:

Please note that the links are separated in different lines. To access, please copy and paste the full link to a web browser. The links work when copy and paste. Also, please be aware if extra space is presented behind "/" in the copied links.

**References
OK.

Other notes:

In addition to the recommended changes by the reviewers, we have made a few other modifications. Importantly, to estimate dTEC spatial scales, we do not take any assumptions about spatial variations as we did in the previous version. Instead, we have estimated the plasma flow velocity from an average Doppler shift factor of ~1.15 measured in Figure 4j and pierce point velocity, using $\frac{\vec{v}_{ipp} \cdot \vec{v}_{struct}}{|\vec{v}_{struct}|^2} \sim 0.15$. This enables us to estimate the scale sizes based on and $ds = \left(|\vec{v}_{struct}| - |\vec{v}_{ipp}|\right) \cdot dt$. Please refer to the revision-highlighted manuscript for these changes.
