# Supplementary material for: Magnetospheric Control of Ionospheric TEC Perturbations via Whistler‐Mode and ULF Waves
Source: AGU Adv. 2024 Nov 25;5(6):e2024AV001302. doi: 10.1029/2024AV001302 (PMC11600791; doi:10.1029/2024AV001302)
Supplement: Supplementary file 5 — Author Response to Peer Review Comments [file AGA2-5-0-s007.pdf]

Response to Reviewers

We appreciate both reviewers' efforts in evaluating our manuscript and providing very helpful comments. Below we address the comments the reviewers have brought up. Please note that the original review comments are presented in black and our responses are shown in blue.

Reviewer #3:

The authors have done a very good job answering my previous questions and concerns. My only remaining question is about the possibility of the TEC variations due to horizontal transport. In the revised manuscript, vertical transport has been discussed but not horizontal transport. It would be useful to add a short paragraph discussing its possible role.

Response:

Thank you for the suggestion. We have added discussions on horizontal transport as the following:

"*The periodic horizontal drift of ULF waves could produce noticeable TEC modulation across a horizontal density gradient, via the advection term $\vec{v} \cdot \nabla n_e$ (Poole & Sutcliffe, 1987, doi: 10.1016/0021-9169(87)90058-4; Waters & Cox, 2009, doi: 10.5194/angeo-27-2779-2009; Pilipenko et al., 2014, doi: 10.1002/2013JA019594). This modulation may be enabled by a pre-existing east-west density gradient, which was suggested to produce TEC modulation of ~2% with 5 nT magnetic perturbations near the terminator (Waters & Cox, 2009). However, our event was on the nightside, away from the terminator. The advection may arise from a latitudinal density gradient coupled with ULF $\vec{E} \times \vec{B}$ drifts. Pilipenko et al. (2014) estimated that this latitudinal advection could contribute to $dTEC/TEC$ of ~0.1% at auroral latitudes, corresponding to dTEC~0.02 TECU in our case. In general, Poole&Sutcliffe (1987) theoretically derived the advection-induced TEC modulation as $dTEC/TEC \sim 2E_y/\omega B_0 L$, where L is the horizontal gradient scale. If we take E_y~ 1 mV/m, ω~ 10^{-2} s^{-1}, L~ 30 km, the resulting $dTEC/TEC$ is only 0.17%. Thus, ULF-induced horizontal transport also cannot explain our observed dTEC modulation of ~0.5 TECU.*"

Reviewer #4:

This is a review for Magnetospheric control of ionospheric TEC perturbations via whistler-mode and ULF waves by Shen et al.

I believe the authors have properly addressed the most important points raised by both reviewers. The supplemental information and additional explanations in the text significantly improve the paper. This manuscript is ready for publication after the following very minor corrections:

Lines 183 - 193: Please cut this sentence into two parts for clarity. Otherwise nice explanation.

Response:

Done as suggested.

Figure 2: While I note that the figure has been improved it is still significantly cramped. Would it be possible to remove some information that is not necessary, such as some of the scales (do we need every 10^n to understand the scale on the right-side axis? you can also better adapt the numbers on the left-hand axis). At minimum, consider a top-to-bottom presentation so the figures are more readable.

Response:

Thank you for the suggestion. We have changed the figure to make it more concise and better organized.

Line 386: Santolik not SantoliK

Response:

Changed accordingly.
